# Effectiveness of Virtual vs In-Person Inhaler Education for Hospitalized Patients With Obstructive Lung Disease: A Randomized Clinical Trial

**DOI:** 10.1001/jamanetworkopen.2019.18205

**Published:** 2020-01-03

**Authors:** Valerie G. Press, Vineet M. Arora, Colleen A. Kelly, Kyle A. Carey, Steve R. White, Wen Wan

**Affiliations:** 1 Section of General Internal Medicine, Department of Medicine, University of Chicago, Chicago, Illinois; 2 Pritzker School of Medicine, University of Chicago, Chicago, Illinois; 3 Section of Pulmonary and Critical Care Medicine, Department of Medicine, University of Chicago, Chicago, Illinois

## Abstract

**Importance:**

Many patients who are hospitalized cannot use inhalers correctly, yet education for their use is often not provided. To address the need for an effective intervention feasible for wide-scale implementation, a virtual teach-to-goal intervention was developed to provide tailored patient-directed education using adaptive learning technology.

**Objective:**

To assess whether the virtual teach-to-goal intervention is noninferior to an in-person teach-to-goal intervention for improving inhaler technique.

**Design, Setting, and Participants:**

An equivalence and noninferiority randomized clinical trial took place from January 13, 2016, through September 20, 2017, with analyses conducted between October 25, 2017, and September 23, 2019. Adult inpatients with asthma or chronic obstructive pulmonary disease (COPD) admitted to general inpatient wards were eligible. Enrolled participants were randomized to virtual (n = 61) or in-person (n = 60) educational interventions. Investigators and research assistants were masked to interventions. Initial enrollment, study assessments, and delivery of the educational intervention occurred in the hospital; participants returned at 30 days for a follow-up research visit.

**Interventions:**

Virtual education was a module delivered via handheld tablet with self-assessment questions before demonstration, narrated video demonstration of the correct technique, and self-assessment questions after demonstration; up to 3 rounds were repeated as needed. In-person education participants received iterative rounds of inhaler technique assessment and education by trained staff.

**Main Outcomes and Measures:**

Noninferiority testing of whether virtual vs in-person education achieved an equal percentage with correct inhaler technique after education (>9 of 12 steps correct) against an a priori noninferiority limit of –10%; logistic regression models were used to adjust for differences in baseline technique and health literacy.

**Results:**

Among 118 participants (59 in each group), most were black (114 [97%]) and female (76 [64%]), with a mean (SD) age of 54.5 (13.0) years. Correct technique increased similarly before vs after education in virtual (67%; range, 2%-69%) and in-person (66%; range, 17% to 83%) groups, although the difference after intervention exceeded the noninferiority limit (–14%; 95% CI lower bound, –26%). When adjusting for baseline inhaler technique, the difference was equivalent to the noninferiority limit (–10%; 95% CI lower bound, –22%).

**Conclusions and Relevance:**

The findings suggest that patient-directed virtual education similarly improved the percentage of participants with correct technique compared with in-person education. Future work should confirm whether virtual teach-to-goal education is noninferior to in-person education and whether it is associated with long-term skills retention, medication adherence, and improved health outcomes.

**Trial Registration:**

ClinicalTrials.gov identifier: NCT02611531

## Key Points

**Question:**

Among patients hospitalized with asthma or chronic obstructive pulmonary disease (COPD), is a patient-driven virtual educational intervention as effective as the criterion standard in-person teach-to-goal intervention for improving medication use skills?

**Findings:**

In this equivalence and noninferiority trial of 118 adult inpatients with asthma or COPD, the increase in the percentage of persons with improved medication skills was similar in the virtual teach-to-goal group (67%) and the in-person teach-to-goal group (66%), although the postintervention group comparison did not meet the a priori noninferiority significance level.

**Meaning:**

The findings suggest that, for adults hospitalized with asthma or COPD, the novel virtual intervention provides guideline-recommended care nearly as well as the time-intensive in-person strategy.

## Teaser

This equivalence and noninferiority randomized clinical trial assesses whether a virtual teach-to-goal intervention is noninferior to an in-person teach-to-goal intervention for improving inhaler technique among adult patients hospitalized with asthma or chronic obstructive pulmonary disease.

## Introduction

Among the 1 million hospitalizations for exacerbations of asthma or chronic obstructive pulmonary disease (COPD) annually, it is unknown how many could be avoided.^1,2^ Most hospitalizations are thought to be preventable^3,4^ because highly efficacious treatments exist to prevent and treat respiratory symptoms.^5,6^ However, these treatments are primarily delivered via respiratory inhaler devices, which can be difficult to use correctly.^7,8^ Guidelines recommend assessing and teaching inhaler technique at all health care encounters.^5,6^ Unfortunately, this guideline-recommended care is often not provided, especially in the hospital.^9,10^ Unsurprisingly, most patients misuse their devices.^11^

Inhaler misuse has not improved during the past 4 decades and is costly.^8,12,13,14,15,16,17,18,19,20^ Of the $25 billion spent annually on inhalers, $5 billion to $7 billion is lost.^13^ This amounts to as much as $900 per month per patient wasted on incorrectly used inhalers.^12,13,14,15,16,17^ Beyond the financial costs of inhaler misuse are costs to patient health outcomes, including worse symptom control, poorer quality of life, and increased acute care utilization.^18,19,21^ Despite these costs to patients and health care systems, no feasible educational interventions exist for widespread dissemination across diverse populations to date.^8^

In-person education, especially using teach-to-goal (TTG) methods with rounds of demonstration and the teach-back method, has been shown to be superior to brief verbal instructions for improving inhaler technique and decreasing acute care utilization.^22,23^ However, in-person education is expensive, difficult to implement, and of varying quality. Without a low-cost, easy-to-implement solution, millions of patients may continue to lack fundamental care and face preventable exacerbations.

A direct-to-patient, technology-based intervention designed to be a low-cost, feasible solution was developed by members of our team at the University of Chicago, some of whom are authors on this report; this intervention is housed and licensed on the Smart Sparrow platform.^24,25^ However, it is unknown whether this virtual educational intervention is as effective as in-person education. Our primary objective was to test whether this virtual intervention was as effective as in-person education for improving inhaler technique among patients hospitalized with COPD.

## Methods

### Design, Study Setting, and Participants

The Virtual vs In-Person TTG Respiratory Inhaler Technique Assessment and Instruction (V-TRaIN) study was a randomized, controlled, noninferiority trial to evaluate the comparative effectiveness of virtual TTG (V-TTG) vs in-person TTG educational interventions for instructing inpatients with asthma or COPD on correct metered-dose inhaler use (trial protocol in Supplement 1). Recruitment occurred from January 13, 2016, through August 30, 2017. Thirty-day follow-up assessments were completed by September 20, 2017. Analyses were completed between October 25, 2017, and September 23, 2019. The University of Chicago institutional review board approved the study. Written informed consent was obtained from all enrolled participants.^26,27,28^ This study followed the Consolidated Standards of Reporting Trials (CONSORT) reporting guideline.^29^

A noninferiority design was chosen because our objective was to assess whether V-TTG is as effective but not more effective^29,30^ than in-person TTG for initial education.^29,30,31,32,33^ Participants were recruited from an urban academic hospital and were similar to participants in previous studies evaluating efficacy of interventions tested in this study.^11,22,23,24^ Hospitalized adult patients aged 18 years or older with physician-diagnosed asthma or COPD were eligible if they were admitted to an inpatient ward (not intensive care unit), if they were discharged home with metered-dose inhalers for rescue and/or controller medications, if they passed vision screening, and if inpatient teams provided assent. Data were stored in the secure, web-based Research Electronic Data Capture tool.^34^

### Randomization

Participants were randomized to V-TTG or in-person TTG (eFigure 1 in Supplement 2) using a randomization schedule provided a priori by the study biostatistician (Stata, version 14; StataCorp LLC) that remained concealed in sealed envelopes until participants were randomized. A project manager assigned participants to educational interventions delivered by research educators using a block-randomized schema stratified by health literacy (Short Test of Functional Health Literacy in Adults).^27,28^ Research assistants completed baseline measures required for randomization and remained blinded to the educational intervention throughout the study, including baseline, posteducation, and follow-up visits. Except for staff turnover, the same research assistant conducted assessments across time points. Study investigators were blinded until all enrollment and data collection were complete.

### Intervention

#### V-TTG Intervention

The development and efficacy testing of V-TTG using patient focus groups, surveys, and iterative efficacy testing was published previously.^10,23^ Screenshots of the V-TTG intervention were also published previously (eFigure 2 in Supplement 2).^24,25^ Participants assigned to V-TTG were provided with a tablet device to complete the intervention, which consisted of self-assessment questions before demonstration, narrated video demonstration of correct technique, and self-assessment questions after demonstration; cycles of the narrated demonstration and self-assessment questions were repeated, as needed, until all questions were answered correctly or 3 rounds were completed.^10,23^

#### In-Person TTG Intervention

Participants randomized to the in-person TTG intervention received iterative (up to 3) rounds of inhaler technique education with evaluation provided by the research educator as published previously.^11,22,23^

### Measures

During the initial inpatient and 30-day follow-up study visits, the resident assistant collected data on demographic characteristics, medical history, and medication use. Lung function was assessed using a portable spirometry KoKo PFT System, version 4.3 (nSpire Health Inc) for participants meeting safety criteria (eg, safe blood pressure, no recent thoracic surgery) based on published guidelines.^35^ Inhaler technique was evaluated by a trained assessor using a 12-step checklist validated and used in previous intervention efficacy studies (*k* = 0.94) (eFigure 3 in Supplement 2).^11,22,23,24^

#### Primary Outcome

The primary outcome was correct inhaler technique (≥10 of 12 steps correct) after education. This cutoff of inhaler misuse was based on previously published research establishing intervention efficacy.^11,22,23^

#### Secondary Outcomes

Secondary outcomes included correct inhaler use before and after education within intervention groups, posthospital education vs 30-day correct inhaler use after discharge within and across intervention cohorts, and health care utilization for 30 days after discharge.

### Statistical Analysis

A conservative a priori noninferiority margin of –10% for the difference (V-TTG minus in-person TTG) in the percentage of participants with correct inhaler use at discharge was chosen^29,30^ because of concerns that clinicians would be skeptical of non–in-person approaches. On the basis of previous studies, we anticipated the percentage with correct use immediately after education to be approximately 88%; given this, a sample size of 59 participants per group would yield an approximate 95% CI 1-sided lower bound 10 percentage points less than the observed difference between the 2 groups.^11,22^ Although a standard sample size calculation for establishing noninferiority in this setting would result in a larger sample size, the available resources precluded performing a larger trial.

The a priori planned primary analysis was an unadjusted comparison of the percent of participants using the inhaler correctly (≥10 of 12 steps correct) at discharge between those assigned to V-TTG and those assigned to in-person TTG. All consenting participants who completed the primary outcome assessment before discharge were included in the analyses. Adjusted comparisons were performed using logistic regression (percentage with correct technique) and ordinal logistic regression (total number of steps performed correctly).^36^ A 95% CI 1-sided lower bound was computed using the standard normal approximation and was compared with the noninferiority margin of –10%.^37^ To facilitate comparison with the unadjusted results, we computed the marginal probabilities (presented as percentages for consistency) of obtaining a score at least 10 correct steps separately for each group, the difference between them, and a 95% CI lower bound (again using the normal approximation with SEs obtained with the method). The marginal probability for a given group was computed by calculating the predicted probability for each individual based on the model assuming that all individuals are in the given group (but leaving the value of the other covariates unchanged) and averaging the entire sample.^38^ Covariates included baseline correct use score and baseline health literacy (binary). Comparisons at 30 days after discharge were also performed.

To examine treatment effects over time, we fit a mixed-effects ordinal logit model to the observed scores at baseline, discharge, and 30 days after discharge (ie, 3 observations per participant)^39^:

logit[P(Y_ij_ > k)] = β_1_ × V_i_ + β_2_A_ij_ + β_3_D30_ij_ + β_4_V_i_ × A_ij_ + β_5_V_i_ × D30_ij_ + Z_i_γ + *θ_i_* – κ_k_

where Y_ij_ is the number of steps performed correctly by the *i*th participant at the *j*th time point (*j* = 1, 2, 3 and *k* = 1, 2, …, 11). The variable V_i_ = 1 for those assigned to V-TTG, 0 otherwise; A_ij_ = 1 for those time points after the educational intervention, 0 otherwise; D30_ij_ = 1 for the time point 30 days after discharge, 0 otherwise; and Z_i_ defined a vector of covariates (eg, an indicator of low health literacy). Thus, the coefficient β_4_ captured the differential effect of V-TTG vs in-person TTG on performance at discharge, whereas β_5_ captured the difference between V-TTG and in-person TTG in the change during the first month after discharge. Finally, *θ_i_* is a random intercept capturing stable differences between participants. The model was estimated using maximum likelihood assuming that the *θ_i_* is normally distributed.

For the secondary outcome, we dichotomized 30-day acute care events (both emergency department and hospitalization visits) and then conducted a noninferiority analysis using logistic regression to model the likelihood of having an acute care event (both emergency department and hospitalization visits) within 30 days after education. Consistent with the analysis of the primary outcome, we presented a 95% CI upper bound for the risk difference between V-TTG and in-person TTG using the same noninferiority margin of 10%. The number of acute care events at baseline (previous 12 months) and health literacy were included as covariates. Both SAS, version 9.4 (SAS Institute Inc) and Stata, version 16 (StataCorp) software were used for analyses. A 1-sided *P* < .05 was determined to be statistically significant.

## Results

**Figure 1.**
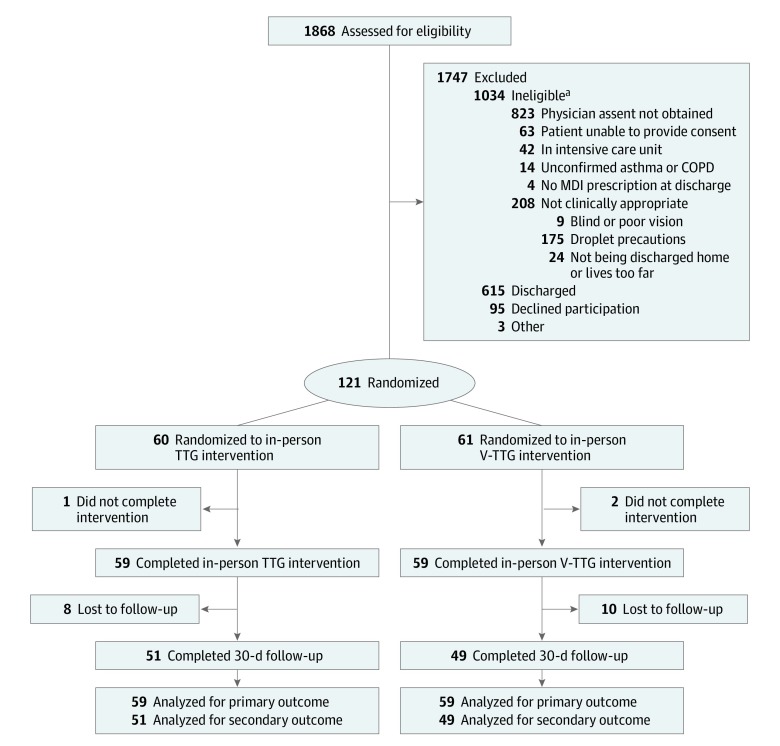
Flowchart of Patients Through the Trial COPD indicates chronic obstructive pulmonary disease; MDI, metered dose inhaler; TTG, teach-to-goal; V-TTG, virtual teach-to-goal. ^a^May add to more than 1034 persons because of multiple exclusion factors.

**Figure 2.**
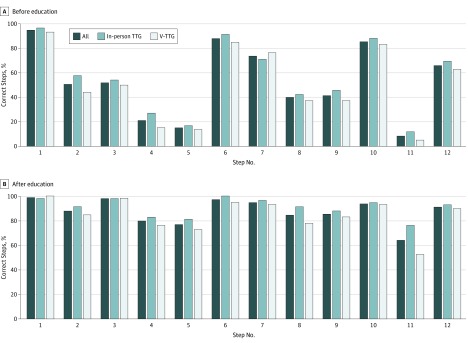
Metered Dose Inhaler Technique Steps Correct Before and After Education TTG indicates teach-to-goal; V-TTG, virtual teach-to-goal.

Of 1868 patients screened for eligibility, 121 were enrolled and randomized to V-TTG (n = 61) and in-person TTG (n = 60); 118 participants (59 per cohort) completed the inpatient study assessments (Figure 1). Most participants were black (114 [97%]) and female (76 [64%]), with a mean (SD) age of 54.5 (13.0) years. Most participants had COPD (69 [58%]) and adequate health literacy (87 [74%]). There were no major differences in baseline characteristics among the 2 cohorts except a smaller percentage of participants had correct baseline inhaler technique in the V-TTG cohort (1 [2%]) than in the in-person TTG cohort (10 [17%]) (*P* = .008) (Table 1). The most common missed steps were emptying lungs (step 4), emptying lungs away from the device (step 5), and breathing normally between puffs (step 11) (Figure 2A). The mean (SD) time to complete the intervention was 23.4 (9.7) minutes for V-TTG and 9.7 (4.4) minutes for in-person TTG (*P* < .001).

**Table 1.** Participant Characteristics^a^

Characteristic	Total Participants (N = 118)	In-Person TTG Group (n = 59)	Virtual TTG Group (n = 59)
Age, mean (SD), y	54.5 (13.0)	53.9 (12.2)	55.1 (13.7)
COPD, No. (%)	69 (59)	34 (58)	35 (59)
Female, No. (%)	76 (64)	41 (70)	35 (64)
Race/ethnicity, No. (%)			
Black	114 (97)	58 (98)	56 (95)
Non-Hispanic or Latino	103 (100)	52 (100)	51 (100)
At least high school graduate, No. (%)	82 (71)	45 (78)	37 (65)
Adequate health literacy, No. (%)	87 (74)	44 (76)	43 (73)
Visited a health care clinician for asthma or COPD care, No. (%)	74 (63)	33 (56)	41 (70)
Hospitalized in the previous 12 mo ≥1 time, No. (%)	73 (62)	35 (59)	38 (64)
Near-fatal respiratory event ≥1 time, No. (%)^b^	67 (57)	30 (50)	37 (63)
% FEV_1_ predicted, mean (SD)^c^	1.32 (0.6)	1.28 (0.7)	1.37 (0.5)
FVC, mean (SD), L/s^c^	1.95 (0.7)	1.91 (0.7)	2.0 (0.6)
FEV_1_:FVC, mean (SD), L/s^c^	0.67 (0.2)	0.66 (0.2)	0.68 (0.1)
Baseline level of use, No. (%)			
Correct use	11 (9)	10 (17)	1 (2)
Mastery	0	0	0

Abbreviations: COPD, chronic obstructive pulmonary disease; FEV_1_, forced expiratory volume in the first second of expiration; FVC, forced vital capacity; TTG, teach-to-goal.

^a^ The intervention cohort (virtual TTG) had a higher proportion of baseline misuse compared with the comparator cohort (in-person TTG): *P* = .008 by a Fisher exact test; all other characteristics by either Fisher exact test or Wilcoxon rank sum test had *P* ≥ .15.

^b^ Near-fatal events defined as ever having been intubated and/or admitted to an intensive care unit.^11^

^c^ Lung function data missing (available for 55 participants).

### V-TTG vs In-Person TTG Immediately After Education

Most participants (91 of 118 [77%]) completed the interventions on days 2 or 3 of hospitalization. Forty-one of the 59 participants (69%) randomized to V-TTG scored at least 10 when assessed at discharge compared with 49 of 59 participants (83%) in in-person TTG group, although the percentage of participants with correct technique increased similarly in both the V-TTG (from 2% to 69%; difference, 67%) and in-person TTG (from 17% to 83%; difference, 66%) groups (eTable in Supplement 2). The difference in the observed rate (V-TTG minus in-person TTG) was –14% (95% CI lower bound, –26%) (Table 2). A logistic regression model (model 1) adjusting for number of baseline correct steps yielded marginal probabilities of scoring at least 10 of 71% (V-TTG) and 82% (in-person TTG), for a difference of –10% (95% CI lower bound, –22%); adjusting for low health literacy (model 2) yielded the same estimated difference and confidence bound. Fitting an ordinal logit model with both covariates yielded a similar difference and greater precision (–11%; 95% CI lower bound, –19%). Similar improvements in the individual steps missed were seen across both groups for all inhaler technique steps except for step 11 (53% correct in the V-TTG group vs 76% correct in the in-person TTG group; *P* = .01) (Figure 2B); however, this difference was smaller when adjusting for baseline misuse (V-TTG: 49% vs 64%; *P* = .10).

**Table 2.** Estimated Percentage of Patients With Correct Inhaler Technique by Intervention Group^a^

Event	In-Person TTG Group	Virtual TTG Group	Difference (SE), %	95% CI Lower Bound, %
**Discharge**				
Observations, No.	59	59	NA	NA
Observed, %	83	69	−14 (8)	−26
Marginal, %^b^				
Model 1	82	71	−10 (7)	−22
Model 2	82	72	−10 (7)	−22
Model 3	81	70	−11 (5)	−19
**30 d After Discharge**				
Observations, No.	51	49	NA	NA
Observed, %	63	53	−10 (10)	−26
Marginal, %^b^				
Model 1	61	55	−6 (9)	−21
Model 2	60	56	−4 (9)	−18
Model 3	64	52	−12 (7)	−23

Abbreviations: NA, not applicable; TTG, teach-to-goal.

^a^ Correct inhaler score equals at least 10 of 12.

^b^ Estimated by setting all observations to in-person TTG (or virtual TTG) and averaging estimated percentages over the observed sample. Model 1 represents logistic regression adjusting for baseline score, model 2 represents logistic regression adjusting for baseline score and health literacy (binary indicator for low literacy), and model 3 represents ordinal logistic regression of score (range, 1-12) adjusting for baseline score and health literacy.

### V-TTG vs In-Person TTG at 30-Day Follow-up Visit

Of 118 participants, 100 (85%) completed the 30-day follow-up visit (49 of 59 in the V-TTG group and 51 of 59 in the in-person TTG group). Twenty-six of 49 (53%) in the V-TTG group scored at least 10 compared with 32 of 51 (63%) in the in-person TTG group, yielding an observed difference in the rates of 10% (95% CI lower bound, –26%) (Table 2). Adjusting for baseline score (model 1) yielded smaller differences of –6% (95% CI lower bound, –21%), and both baseline score and low health literacy (model 2) yielded smaller differences of –4% (95% CI lower bound, –18%). The estimated difference based on the ordinal logit model was larger than the unadjusted difference but with higher precision (–12%; 95% CI lower bound, –23%).

### V-TTG vs In-Person TTG at All 3 Time Points

Fitting a mixed-effects ordinal logit model to the data from all 3 time points yielded estimated increases in the logit (ie, log[p / (1 – p)]) of scoring at least 10 immediately after the intervention of 5.66 for in-person TTG and 5.66 – 0.65 = 5.01 for V-TTG (Table 3). Thus, the estimated immediate effect of V-TTG (on the logit scale) was 88% as large as that for in-person TTG (95% CI lower bound, 75%), consistent with the comparisons in Table 3. As noted earlier, performance declined in the in-person TTG group during the first month after discharge by an amount that was approximately one-third that of the initial increase (–2.19; *P* < .001). This decline was smaller in the V-TTG group (–2.19 – 0.27 = –1.92), although this difference was small compared with its SE (*P* = .61). The overall increase from baseline to 30 days after discharge in the V-TTG group (3.16) was 89% as large (95% CI lower bound, 67%) as that for the in-person TTG group (3.54). Those with low health literacy scored lower on average (odds ratio, 0.26; 95% CI, 0.11-0.59; *P* = .001); despite adjusting for this, there remained substantial between-individual differences assuming that the *θ_i_* is normally distributed with mean of 0 (SD of random intercept, 1.7). An interaction between the postintervention indicator and low health literacy yielded an estimated difference in differences value of –0.80 (95% CI, –1.80 to 0.19; *P* = .10), consistent with the possibility that the effect of an educational intervention may be lower among those with low health literacy.

**Table 3.** Estimated Coefficients From Mixed-Effects Ordinal Logit Model

Covariate	Estimate (95% CI)	*P* Value
Intervention, virtual vs in-person TTG	−0.66 (−1.56 to 0.24)	.15
After intervention, discharge or 30 d^a^	5.66 (4.69 to 6.63)	<.001
30 d, vs baseline or discharge^b^	−2.19 (−2.90 to −1.33)	<.001
Interaction between intervention and after intervention	−0.65 (−1.63 to 0.33)	.19
Intervention interaction at 30 d	0.27 (-0.76 to 1.30)	.61
Low health literacy	−1.36 (−2.21 to −0.52)	.001
SD, #, θ^c^	1.67 (1.31 to 2.13)	<.001^d^

Abbreviation: TTG, teach-to-goal.

^a^ Equal to 1 after intervention (ie, discharge and 30 days) and 0 at baseline.

^b^ Equal to 1 at 30 days and 0 otherwise.

^c^ Estimated SD of random intercept.

^d^ Likelihood ratio test that variance component is equal to 0, based on a 50:50 mixture of a χ^2^ distribution with 1 *df* and a point mass at 0.

### All-Cause Acute Care Utilization

At baseline, both V-TTG and in-person TTG groups reported similar rates of all-cause acute care utilization 1 year before study enrollment (Table 1). The V-TTG participants reported a mean (SD) of 2.1 (1.7) emergency department visits and 1.8 (1.6) hospitalizations compared with the in-person TTG participants, who reported a mean (SD) of 1.9 (1.6) emergency department visits and 1.4 (1.5) hospitalizations. After 30 days, the percentage of participants who had experienced acute care emergency department or hospital utilization was lower for V-TTG (24%) than for in-person TTG (29%) participants, yielding an unadjusted difference of –5% (95% CI upper bound, 9.6%). Adjusting for the baseline number of acute events and health literacy changed this difference to –8% (95% CI upper bound, 7%). Using the noninferior margin of 10%, V-TTG was noninferior to in-person TTG in probability of 30-day acute events.

## Discussion

We found that a virtual-delivered, patient-directed intervention for inhaler instruction increased inhaler technique proficiency by a similar amount as a well-validated in-person approach. Moreover, performance in the V-TTG group declined by the same amount as in the in-person TTG group in the month after discharge, implying that skills learned with V-TTG may be as durable as those learned with in-person TTG. Thus, it appears that learners are well served by V-TTG, especially because it may be repeated at home. This virtual approach has potentially important implications for increasing access to high-quality education because the virtual intervention likely has significantly lower costs and time constraints in real-world settings compared with the costs of training and delivering in-person education in hospital and at home.

The V-TTG nearly met our a priori noninferiority criteria compared with in-person TTG, especially when specifically evaluating the study population with baseline inhaler misuse. The estimated differences in the rates of inhaler proficiency at discharge and at 30 days were similar to our a priori noninferiority margin of 10%. This margin was conservative; with hindsight, a more liberal noninferiority margin could have been justified clinically given the higher feasibility of disseminating the virtual educational intervention with potentially broader reach in real-world practice and given our findings that decline of inhaler technique skills was similar with both virtual and in-person approaches. Future studies designed to demonstrate statistical noninferiority might consider a more liberal noninferiority margin because even if in-person TTG is somewhat more efficacious, V-TTG’s greater feasibility would make it an attractive option in real-world settings. More importantly, future studies should be designed to observe patients for a long time such that V-TTG can be repeated at any time, potentially increasing its value compared with in-person TTG. Because this was a single-site study in an urban setting with predominantly black individuals, future studies should also compare the efficacy of V-TTG vs in-person TTG in diverse settings and populations.

Innovation appears to be necessary to solve the fundamental problem of how to deliver guideline-recommended care in real-world settings because the practice of relying on health care clinicians has not proved to be effective.^8^ The novel approach of V-TTG is that it may not only be effective but also offer a solution to system barriers that prevent education from occurring in the first place. Of importance, in our study, similar improvements occurred in individual inhaler steps and overall technique among participants with baseline misuse in the V-TTG and in-person TTG groups.^5,6,40^ Because inhaler education is often substandard or lacking, patients commonly misuse their inhalers and thus do not receive the clinical benefits. Virtual TTG also allows for ongoing refresher or practice education because this teaching method could be done in homes or other non–health care settings. This aspect of V-TTG provides a system-based solution to the need for repeated education^23^ because our work and other studies have found that inhaler skills wane quickly after education.^23^ Effective patient-driven interventions that can work in any setting could be beneficial and improve long-term outcomes. Real-world studies are needed to test the feasibility of large-scale implementation of V-TTG across clinical and home settings and whether repeated administration of virtual education improves long-term outcomes.

To date, the relationship between inhaler technique and health outcomes has been difficult to study in real-world settings because of limited availability of discrete administrative data. In previous studies, improved technique was associated with short-term improved health outcomes, including fewer 30-day acute care events among participants receiving in-person TTG vs brief verbal instructions.^22,23^ In this study, the percentages of participants in both groups with acute care visits (emergency department visits or hospitalization) at 30 days were similar. More data are needed to understand whether V-TTG can further improve health outcomes if used for refresher education. In addition, outside controlled research studies, documentation of education, especially inhaler technique, is frequently lacking. Therefore, little is known about the real-world impact of inhaler education. Technology that captures patients’ responses may permit measurement of the inhaler educational intervention’s effect on health outcomes in real-world settings.

Another potential advantage of V-TTG is identification of patients who may benefit from additional in-person training. Because interventions that rely on technology may not be suitable for all patients, if V-TTG proves to be inaccessible to some patients, in-person education could be provided. Using in-person education for a smaller and preidentified high-risk patient population could be less costly than providing it for all inpatients. Investigations are needed into practical methods to deploy V-TTG for screening of high-risk patients who need hands-on training.

### Limitations

This study has limitations. First, our study population primarily comprised urban, underserved, black patients, with a slightly higher proportion of these patients than our clinical population, and our intervention was developed using direct feedback from this population. Also, trained nonclinical research personnel provided in-person TTG education. However, our validated checklists have high interrater reliability^11^; thus, this approach could be adapted by clinical staff. Therefore, future work is needed to test generalizability among diverse patient populations in clinical settings.

An important methodologic limitation is the significant difference in baseline inhaler misuse between V-TTG and in-person TTG groups. This imbalance adds complexity to deciphering the findings, although overall results were not different (noninferiority not met). Future studies across diverse settings and populations with similar baseline rates of misuse are needed to validate our findings. Another important limitation is that participants were provided with devices to complete V-TTG education; thus, data are needed on technology access for such education in hospitals and homes. Although most US adults report mobile device ownership, device size, and/or willingness to use, data plans and service may vary across patient subgroups and setting which could affect real-world use of V-TTG.^41,42^ Therefore, generalizability to clinical and home settings across diverse populations and geographies needs testing in future head-to-head comparisons. Although V-TTG may be a cost-effective system-based solution to providing effective guideline-recommended inhaler education, real-world testing is needed. For instance, if clinician or staff time is needed for V-TTG, the hypothesized cost-savings could be mitigated because completion of V-TTG takes significantly longer than in-person TTG.

## Conclusions

This study found that virtual TTG may be nearly as effective as in-person education for correcting baseline inhaler misuse among hospitalized patients. Larger-scale pragmatic studies are needed to determine whether V-TTG will improve long-term inhaler technique skills and/or patient outcomes when used across health care and home settings and to evaluate feasibility and potential cost-savings of wide-scale implementation. If V-TTG is shown to be feasible and effective across diverse populations and settings, this innovation could transform the ability of health care systems and individual clinicians to deliver guideline-recommended education for inhalers effectively with the goal of improving health outcomes and avoiding exacerbations.

## Notes

Supplement 1. **Trial Protocol**

Supplement 2. **eFigure 1.** A Conceptual Model of In-person TTG and V-TTG **eFigure 2.** Virtual Teach-to-Goal Screenshots **eFigure 3.** Metered Dose Inhaler Checklist **eTable.** V-TTG versus TTG Results

Supplement 3. **Data Sharing Statement**
