# Supplementary material for: Effectiveness of Virtual vs In-Person Inhaler Education for Hospitalized Patients With Obstructive Lung Disease: A Randomized Clinical Trial
Source: JAMA Netw Open. 2020 Jan 3;3(1):e1918205. doi: 10.1001/jamanetworkopen.2019.18205 (PMC6991242; doi:10.1001/jamanetworkopen.2019.18205)
Supplement: Supplement 1. — Trial Protocol [file jamanetwopen-3-e1918205-s001.pdf]

Table of Contents: Protocols

*Please note this was for my K23 which had two projects, a pre/post (Aim 1) and the non-inferiority RCT (Aim 2). I am submitting all versions of the protocols, though some are updates to Aim 1 which is not related to the submitted manuscript. We have published those data previously (Press et al. JACIP, 2017).

Protocols

1. October 9, 2012-first submission for NIH Just In Time (JIT)
2. November 29, 2012
3. August 31, 2014
4. March 18, 2015
5. October 19, 2015
6. June 9, 2016
7. May 15, 2017
8. Consent form (Round 2= Aim 2)

Protocol Title: Video vs. TTG Respiratory inhaler technique Assessment and InstructioN (V-TRaIN)

University of Chicago
PI: Valerie G. Press, MD, MPH

Co-Investigators: David Meltzer, MD, PhD, Vineet Arora, MD, MAPP, Ted Naureckas, MD

Research personnel: Kristin Constantine, Nicole Babuskow, Danielle Blazek, Lisa Spampinato, Laura Ruth Venable

BACKGROUND

Chronic obstructive pulmonary disease (COPD) results in nearly half a million hospitalizations in the United States annually[1] and is the third leading cause of 30-day re-hospitalizations.[2] Clinical trials have established the efficacy of treatments primarily dispensed via respiratory inhaler devices that reduce morbidity and health care utilization if they are used correctly.[3-4] Unfortunately, the effectiveness of these medications in real-world settings is limited by the fact that patients often do not use inhalers correctly.[5-6] Current guidelines recommend assessing and teaching inhaler technique at all health care encounters, including hospitalization.[7] My work has found that over 75% of hospitalized patients in an urban, predominantly underserved population misuse their respiratory inhalers, highlighting a missed opportunity to educate these patients with high potential to benefit.[8] Hospitalization, therefore, provides a potential ‘teachable moment’ to correct this misuse.[9] My preliminary data indicate that one strategy, in-person teach-to-goal (TTG), is effective in teaching hospitalized patients proper inhaler technique and is more effective than simple verbal instruction.[10]

While TTG is a promising method to improve care for patients who use inhalers, several limitations prevent widespread adoption. First, TTG relies on in-person assessment and education, as well as training and monitoring instructors to ensure fidelity, making it time-consuming and costly. Also, because a single educational session does not ensure long-term retention,[11-12] post-discharge reinforcement may be needed, which may be impractical with in-person TTG. One potential method to surmount TTG's limitations is use of interactive video module education (VME), a method that has been used for health education in other clinical contexts.[13-14] Through iterative self-assessments and video-demonstrations on a tablet computer, VME has the potential to be less costly, maintain fidelity, and be more easily extended into the post-discharge setting than in-person TTG. However, certain questions remain about VME. It is unclear whether VME will yield similar results when compared to TTG, or whether urban, underserved patients will have the ability to, and be willing to use, VME in the post-discharge setting. Therefore, before widespread implementation of VME, it is critical to rigorously develop and test VME for inhaler education in the hospital setting. Ultimately, it will also be important to understand patients’ ability and willingness to use post-discharge VME for educational reinforcement to allow for this strategy to transition patients across care settings from hospital to home. Through this ATS award, I will acquire critical data I need to develop R-01 studies focused on improving self-management for patients with obstructive pulmonary disease across care transitions.

<u>We hypothesize that interactive VME will lead to non-inferior rates of ability to demonstrate correct inhaler use compared to rates with TTG among hospitalized patients with COPD.</u>

The specific aims to test this hypothesis are:

1. **To develop and iteratively test VME to teach correct use of metered dose inhaler (MDIs) and dry-powder Diskus® devices to hospitalized patients with COPD.**
   We will partner with Click to Play Media™ (C2P) to develop VME modules that (self)-assess and teach respiratory inhaler technique to hospitalized participants. Once the VME is developed we will iteratively pilot test (n=30) the VME strategy (up to 3 rounds) to obtain important patient feedback and preferences for the module.

2. **To determine if the proportion of hospitalized patients with COPD who demonstrate correct inhaler technique after receiving VME is not significantly less than (non-inferior to) the proportion who demonstrate correct inhaler technique after receiving in-person TTG.**
   To accomplish this aim we will build on our prior work with developing and evaluating the in-person TTG strategy to develop a parallel VME strategy that incorporates guideline-recommended components of assessment and instruction. We will conduct a behavioral randomized clinical trial to evaluate the relative effectiveness and durability of VME versus in-person TTG education on the ability of hospitalized patients to demonstrate correct MDI and Diskus® use. Secondarily, we will assess whether there is variation in comparative effectiveness by patient characteristics including age, gender, and health literacy level, among other important factors.

OVERVIEW OF TRIAL DESIGN

This will be a randomized, controlled, non-inferiority clinical trial conducted of patients admitted to the University of Chicago. Adults admitted with a history of COPD will be eligible to participate in this study. Upon receiving consent, participants will be randomized to either the VME or TTG. Inhaler technique will be measured prior to hospital discharge before and after education (VME or TTG). This will provide data on the short-term effectiveness of the interventions. Retention will be examined at 30 days after hospital discharge.

ELIGIBILITY CRITERIA

The goal of patient selection is to enroll adults admitted with a history of physician-diagnosed COPD.

Inclusion criteria

1. Age 18 years and older
2. Admission to the inpatient medical service and surgical service
3. Physician-diagnosed COPD. We will enroll patients even if the primary reason for admission is COPD (e.g., patients admitted for heart failure, but with a physician diagnosis of COPD are eligible).
4. Physician plans to discharge patients home a metered dose inhaler (MDI; e.g., albuterol)

Exclusion criteria

1. Currently in an intensive care unit
2. Physician declines to provide consent
3. Patient unable to provide consent (e.g., history of cognitive impairment, unable to understand English) or declines to provide consent
4. Previous participant in this study

RECRUITMENT

We will employ the same recruitment plan as in the protocol for our Effectiveness of interventions to Teach Respiratory Inhaler Technique (E-TRaIN) study (Protocol #11-0248). In summary, admission logs (EPIC) at the University of Chicago will be examined each day to screen for potentially eligible patients. The treating physician will be contacted for verbal assent using standardized text. Among patients whose physician provides assent, medical records will be reviewed to ascertain eligibility and informed consent will be sought from eligible patients. A total of 118 patients will be recruited at the University of Chicago Medical Center over 15 months.

TREATMENT CONDITIONS

1. **Teach-to-Goal (TTG):** During inpatient admission, participants assigned to the TTG conditon will be provided with an intensive, iterative education and evaluation strategy that consists of the following steps: First, participants will undergo an initial assessment of respiratory inhaler technique for MDI (and a Diskus® device if they are also prescribed medication using the device, i.e., fluticasone/salmeterol [Advair Diskus ®]) using standardized device-specific checklists (Form 1) that we have developed and used in the SARI study (Protocol #15729A), in TURI (#16900A), and in E-TRaIN (#11-0248). This will be followed by the educational intervention whereby participants will then be taught using verbal instructions and then by demonstration by a trained educator regarding the appropriate use of the MDI (and, if applicable, fluticasone/salmeterol (Advair Diskus®) using standardized written instructions (Forms 2a and 2b). Next the participants will be asked to demonstrate how they use their respiratory inhalers; their post-TTG technique will be graded using the same checklists as the initial assessment. The participants will receive additional rounds of instruction following this assessment if needed (e.g., if the participant did not achieve mastery of the MDI or Diskus® after one round of teaching). Although initial pilot work showed that two rounds was sufficient, more recent data indicates that some participants require > 2 rounds; since the essence of this education technique is to teach-to-goal, and this requires only a few additional minutes (maxium) we will attempt to teach-to-goal. The patient's hospital physician (attending physician or their designee) will be provided information about the patients inhaler technique for both inhalers after education; this will provide an opportunity for the patient to receive addition instructions by the clinical team if needed. All instructions will be provided by a dedicated trained educator; all assessments will be performed by a second research assisstant (trained assessor). The trained assessor will be masked to treatment assignment (see below).[15]
2. **Video Module Education (VME):** The RE will provide participants with a tablet device and will demonstrate how to access VME and complete the pre/post e-learning assessments. The RE will provide technical support but will neither participate directly in the education nor help with the self-assessments. The participants will first complete the pre-assessment e-learning tool on the tablet. They will then watch the video instruction that will provide a complete demonstration with verbal instructions on correct inhaler technique. Next the participants will complete the post-assessment e-learning tool. Based on participants' performance, they will be directed to further tailored video-instruction. The cycle of self-assessment and video instruction will continue until sufficient mastery has been achieved.

Other considerations:

1. *Masking:* To minimize risk of biased measurements of inhaler technique, the trained assessor will be masked to the treatment condition. Specifically, the trained assessor will not be informed about the treatment assignment, will remain outside the patients' room during the educational intervention (and the room door will remain closed during the intervention), and the trained educator will be instructed not to inform the assessor about the treatment condition. We have successfully used similar procedures for masking the trained assessor in other IRB-approved studies (see E-TRaIN, IRB # 11-0248).

2. *Patients being discharged home on a MDI and a Diskus will receive VME or TTG interventions for both devices.* If randomized to the TTG intervention, then TTG will be used to teach MDI and Diskus use; likewise if randomized to the VME intervention, then VME will be used to teach MDI and Diskus use. Based on results of screening data from the TURI study (IRB #16900A), we expect that about 10-20% of participants (10 to 20 participants enrolled in this study) will be prescribed both devices. We would like to include this option to teach two different devices (in patients prescribed both devices) to collect preliminary data about the feasibility of VME for MDI vs. TTG for Diskus; these data would be valuable as we prepare for future studies.

PROTOCOL FOR BOTH TREATMENT GROUPS:

Following the screening procedures above, we will use the following steps:

1. **Obtain assent from treating physician prior to approaching patients hospitalized with asthma or COPD.** The treating physician will be contacted by a research assistant for verbal assent using standardized text (Form 3).

2. **Obtain written informed consent from patient using standardized text.** One hundred eighteen participants (N=59 TTG condition; N=59 VME condition) will be enrolled in this study.

3. **Optimize room conditions**
   - Room well lit (turn on overhead lights if necessary)
   - Patient preferably sitting up
   - Private setting (close door to patient room)
   - Interviewer should directly face patient
   - Patient should use reading glasses if they use one

4. **Assess patient's respiratory symptoms/morbidity (<u>Forms 4-5-6</u>), which includes the** Borg symptom score, Asthma Symptom Severity Index (ASSI), Chronic Bronchitis Symptom Questionnaire, COPD Severity Score (CSS), Airway Questionnaire (AQ-20), COPD Helplessness Index (CHI) and demographic and other clinical information.

5. **Assess patients' self-efficacy regarding respiratory inhaler use (Form 7)**

6. **Assess lung function. Perform spirometry pre and post-bronchodilator (after 2 puffs albuterol MDI via spacer) using portable KoKo spirometer. (Forms 8a and 8b).** These data will provide an objective measure of disease severity at the time of the hospitalization. We have previously demonstrated that this population has poor perception of airflow obstruction and spirometry is feasible during the inpatient setting.[16]

7. **Assess patient's inhaler technique using Inhaler checklists (Form 1;** for a MDI and, if applicable, Diskus®) by the trained assessor.

8. **Assess health literacy. Check vision (Form 9).** Administer STOHFLA questionnaire (Form 10) if the patient's visual acuity is at least 20/50 in one eye (Check the patient's visual acuity in each eye using the Snellen Pocket Eye Chart (Form 11), otherwise, the patient is ineligible to complete the HL evaluation. Explain the purpose of this survey. Use the following text.

   - We want to find out what health care information is understood by patients and what is confusing to them, in order to help us write things more clearly.
   - Here are some medical instructions that you or anybody might see around the hospital. These instructions are in sentences that have some of the words missing.
   - Where a word is missing, a blank line is drawn, and 4 possible words that could go in the blank appear just below it. I want you to figure out which of those 4 words should go in the blank, which word makes the sentence make sense.
   - When you think you know which one it is, circle the letter in front of that word, and go on to the next one.
   - When you finish the page, turn the page and keep going until you finish all the pages.
   - Provide the patient a pen and a clipboard and ask them to complete the questionnaire (Form 10). If the patients ask for help with completing the questionnaire, instruct them to do the best they can and that you are not allowed to assist them. Only responses completed within 7 minutes are used in calculating a health literacy score. Record the start and stop time and any questions that were answered after 7 minutes had elapsed. Do not grade the STOFHLA until after the study visit is complete.

9. **Obtain randomization assignment from the study biostatistician.** Randomization will be block-stratified by level of health literacy to ensure an equal number of subjects within each stratum assigned to TTG and VME Patients will then receive either the TTG or VME intervention by the trained educator.

10. **Collect patient contact information and provide patient $25 in cash for participating in this study as well as parking pass/bus pass for their follow-up visit.** Record patient's social security number and obtain patients signature that they received the patient incentive.

1. **Provide patients an appointment for an in-person interview 30 days after discharge (patients will be given a letter with their appointment time).** At the in-person interview, we will ask patients to complete a short interviewer-administered questionnaire about patient's respiratory symptoms/morbidity/self-reported ability to use MDI and Diskus® inhalers, quality of life (Forms 4 and 12). We will also assess lung function (spirometry) and use of inhalers (MDI and/or Diskus). Upon completing this portion the participant will receive $50 as well as parking pass/bus pass for their next follow-up visit.

OUTCOMES/MEASURES

The primary and secondary outcomes are listed below. Measures for each are described. Initial measurements (Vo; see Data Collection Schedule below) will be made prior to randomization; at V1, the trained educator will assess respiratory inhaler technique by asking the subject to demonstrate use of the MDI (and Diskus® if to be discharged home on fluticasone/salmeterol). Follow-up assessment, V2, will take place at 30 days (+/-7 days) post-hospital discharge.

Primary outcome
The primary outcome will be inhaler technique. We will define inhaler technique in two ways:
a) Correct Use (i.e., >75% of steps correct)
b) Mastery (i.e., perfect technique, 100% steps correct)

This outcome will be measured at V1 and V2. We will test our hypothesis that patients will have a reduced proportion of respiratory events when taught effective inhaler technique with TTG compared to BI education separately for the MDI and Diskus® devices after hospital discharge. We will employ the same checklists used in the E-TRaIN study (IRB Protocol #11-0248, high reliability, kappa >0.94). These checklists will be used to measure the primary outcome: ability to use inhalers (defined as (a) correct use of inhaler: >9/12 steps for MDI; >8/10 steps for Diskus®; and (b) mastery of inhaler technique, i.e., perfect technique: 12/12 steps for MDI; 10/10 steps for Diskus®).

The **primary outcome** is the proportion of participants with MDI misuse post VME vs. TTG education. This will provide data on the short-term effectiveness of the interventions. Longer-term retention (durability) will be examined 30 days post-hospital discharge using an intention-to-treat analysis. We have powered on this binary variable. [Table 1] However, because we recognize that there is more information about which steps are less critical and those that are more critical, I will also work with Robert Gibbons, a nationally recognized expert in biostatistics, to model a mixed effects ordinal regression model with random intercepts and time effects within correlation of each participant with terms for mode (VME and TTG), as well as interaction with time, to determine the predicted probability in each category for VME and TTG of how prevalence of misuse changes over time.[17] **Mediators include** health literacy[18] and vision level[19] as I have previously identified them as important mediators of baseline inhaler misuse and/or ability to learn correct technique.[8,10]

Secondary outcomes
Secondary outcomes will include:
a) Change in self-efficacy for respiratory inhalers (i.e., V0-V2). We will ask all patients to state how strongly they agree or disagree with the following statement "I am confident that I know how to use [insert inhaler name] correctly" for both MDI and Diskus®. The subject will be shown an example of each inhaler. Each item will be scored on a 5-point

Likert scale (strongly disagree to strongly agree); the mean score will be used to measure self-efficacy. Cronbach's alpha will be calculated to evaluate internal consistency.

b) <u>Symptom burden (i.e., BORG, ASSI, and CBSQ) at V1 and V2</u>. Patient reported outcomes will be measured using a modified Borg dyspnea scale (Borg), Asthma Symptom Severity Index (ASSI), Chronic Bronchitis Symptom Questionnaire (CBSQ).

c) <u>Acute care utilization for respiratory exacerbations at V2</u>. Data will be collected using interviewer administrated surveys. *Respiratory events* are defined as: physician, emergency room or hospital visit, or death. This composite endpoint is due to small sample size.

The comparative effectiveness of TTG versus VME with regards to these secondary outcomes will employ simple univariate and/or multivariate logistic regression, generalized estimating equations, or generalized non-linear mixed models, as appropriate. For each of these outcomes, we will evaluate whether health literacy level modifies the effectiveness of VME (Vs. TTG) by including an interaction term in the regression model. Model assumptions will be checked. Use of two inhalers will also provide the opportunity to evaluate if the comparative effectiveness of VME (vs. TTG) varies by device. A two-tailed p-value less than 0.05 will define statistical significance. The sample size calculations demonstrate that N=59 per group will provide 80% power for an intention-to-treat analysis to test for non-inferiority of VME vs. TTG.

Secondary Outcomes include quality of life (QOL),[20] patient reported outcomes,[20-21] lung function**,** utilization of health care services (outpatient visits, ED and hospital admissions, deaths), and self-efficacy of inhaler technique.[8,10] We will also evaluate the differential effects of VME vs. TTG by participant characteristics (e.g., age, health literacy). Finally we will evaluate the preliminary comparative durability of VME vs. TTG at 30 days post-hospital discharge. We will perform all tests separately for both MDI and Diskus devices.

DATA COLLECTION SCHEDULE

**VISIT**	**Inpatient Visit**<br>**V0-V1**		**30 day FU**<br>**V2**
Visit duration (minutes)	60 (total for V0 to V1)		60
Visit location	Inpatient		Outpatient
Visit type	In-person		In-person
Eligibility evaluation	■		
Consent	■		
Randomization	■		
Self Efficacy: Self-reported inhaler technique	■		■
Patient demonstration of inhaler technique	■	■	■
Vision Screen			
Health Literacy	■		
Spirometry before and after bronchodilator	■		■

Review of medications	■		■
Medication count/weigh			■
Questionnaire about symptom burden, respiratory exacerbations, acute care utilization	■		■
Discharge Questionnaire		■	

Vo: initial assessment V1: after instruction V2: 30 days post-discharge follow-up

This study will require 24 months:

1. Develop/pilot test interactive video (6 month)
2. Training of personnel (1 month)
3. Begin recruitment (1-3 participants/week, for 15 months)
4. Complete follow-up of last enrollee (1 month after last enrolled participant)
5. Data analyses and preparation of final report (2 months).

The research will take place on the inpatient wards of UCMC.

ANALYSES

Analysis plan. We will use means, medians, proportions, scatterplots, and histograms to describe the data. We will test for group differences using the *t*-test, the Wilcoxon rank sum test, Pearson's chi-squared test, or Fisher's exact tests as appropriate. We will adhere to the intention-to-treat principle when testing for differences by intervention type. The **primary outcome** will be correct inhaler technique; chi-squared tests will be used to compare post VME vs. post TTG prevalence of inhaler misuse (<75% of steps correct). **Secondary outcomes** will include change in self-efficacy for respiratory inhalers, symptom burden (i.e., BORG and CSS), self-reported utilization of health care services for respiratory exacerbations, basic disease knowledge, and QOL. The comparative effectiveness of VME versus TTG with regards to these secondary outcomes will employ simple univariate and/or multivariate logistic regression, as appropriate. For each of these outcomes, we will evaluate whether patient characteristics (vision, age, and/or health literacy level) modifies the effectiveness of VME versus TTG by evaluating interactions in the regression model. A two-tailed p-value less than 0.05 will define statistical significance. The sample size calculations (see below) demonstrate that n=59 per group will provide 80% power for an intention-to-treat analysis.

Sample size. (Table 1) The main outcome is correct MDI use (>75% steps correct). In my preliminary studies above, the proportion of participants with correct MDI use after TTG was 88-100% (n=98, n=50, respectively).[8,10] These data (expected proportion of correct MDI use of 0.95), along with a noninferiority effect size of -0.10, were used to calculate the sample size.[22] We will enroll 118 participants over 15 months and will have sufficient power (0.8) to determine if VME is non-inferior to TTG, at a significance level of p<0.05. We have experience recruiting participants and have been able to recruit up to 12 participants per month; therefore, we do not foresee any problems with recruiting this number of participants in this timeline.

Table 1: Sample Size per intervention (VME/TTG)

	**Power**				
**EFFECT SIZE**	**0.60**	**0.70**	**0.80**	**0.90**	**0.95**
**-0.05**	137	179	236	326	412
**-0.10**	35	45	59	82	103
**-0.15**	16	20	27	37	46

<u>Interim analyses:</u> After 50% of the target sample size have completed the study (59 participants have completed the study) or after 50% of the study period (~7 months), whichever occurs first,

results of an interim analysis will be provided to a Data Safety and Monitoring Board (Drs. Dana Edelson, MD; Steve White, MD; Neda Laiteerapong, MD; physicians who are not study investigators). Investigators and research assistants will remain masked to the results of the interim analyses until the end of the study. The DSMB will be asked to consider issues related to study performance (enrollment rates, study completion rates) and differences in outcomes between groups and provide recommendations to the PI and the IRB whether to continue the study or terminate enrollment.

HUMAN SUBJECTS

We are not aware of any potential benefits to study subjects. Potential risks include:

1. The subject may be uncomfortable answering some interview questions. They can refuse to answer any question.

2. The lung function test is safe and is commonly used to measure the severity of COPD but can cause some minor chest soreness or lightheadedness; study test will be done by a trained member of the research staff with the subject in a seated position. To further minimize the risk of adverse events with spirometry, we will not perform spirometry in subjects with SBP>180 or DBP>100 mm Hg.

3. Two puffs of albuterol (for post-bronchodilator spirometry) is generally well tolerated. We have not had adverse events with 2 puffs albuterol after enrolling >170 subjects in the SARI, COAT, and TURI studies (IRBs# 15729A, 14831A, 16900A). In some cases, however, 2 puffs of albuterol can cause jitteriness, anxiety, and tachyarrhythmias. To minimize this risk, we will withhold albuterol in subjects who are hospitalized with tachyarrhythmias or palpitations or report problems after using albuterol.

4. Long acting beta-2-agonists (salmeterol or formoterol). These medications are generally well-tolerated, but can have albuterol-like adverse effects; in addition, these agents have been associated with increase risk of asthma-related deaths.

5. Inhaled corticosteroids (fluticasone/budesonide). These medications are well tolerated, but can cause thrush, dysphonia, and other less common adverse effects.

6. Loss of confidentiality. To help ensure that patients' health information remains private, we will restrict access to data collected for our study to study personnel only (via use of password-protection and locked cabinets for study documents). Study ID numbers will be generated and will be used when discussing and/or reviewing data at study meetings. Also, study reports will report results in aggregate and not contain information that can be used to identify individual patients.

Data Safety Monitoring Plan

The PI will review monthly data reports reviewing recruitment, consent, intervention activities, adverse events, and follow-up. Although few, if any, intervention-related adverse events are anticipated in this education-related study, the study population is at risk for poor outcomes, including life-threatening exacerbations and death. The PI will comply with prompt reporting of any unanticipated events and the Data Safety and Monitoring Board (DSMB) (Dana Edelson, MD, Steve White, MD, Neda Laiteerapong, MD). The DSMB (see interim analysis above) will also review results of the interim and final analyses.

PATIENT INCENTIVES

Participants will be provided $25 for completing the initial baseline interview and $50 completing the post-hospital discharge in-person interview. Total possible monetary compensation is $75. Participants will also be provided parking passes or bus passes for their follow-up visits as we recognize that this cost burden should not fall on the participant for participating in our study. All tests for this study will be done free of charge. Monetary compensation will be reduced, based on the extent to which the participante completes the study.

BIBLIOGRAPHY

1. Morbidity and Mortality 2007 Chart Book on Cardiovascular, Lung, and Blood diseases. Available at: http://www.nhlbi.nih.gov/resources/docs/07-chtbk.pdf. Accessed June 06, 2012.

2. Jencks SF, Williams MV, Coleman EA. Rehospitalizations among patients in the Medicare fee-for-service program. N Engl J Med 2009;360(14):1418-1428. PMID: 19339721.

3. Hanania NA, Darken P, Horstman D, Reisner C, Lee B, Davis S, Shah T. The efficacy and safety of Fluticasone Propionate (250ug) /Salmeterol (50ug) combined in the Diskus® Inhaler for the treatment of COPD. Chest 2003;124:834-843. PMID: 12970006.

4. Celli BR, Thomas NE, Anderson JA, Ferguson GT, Jenkins CR, Jones PW, Vestbo J, Knobil K, Yates JC, Calverley PMA. Effect of pharmacotherapy on rate of decline of lung function in chronic obstructive pulmonary disease. Results from the TORCH Study. Am J Resp Crit Care Med 2008;178:332-338. PMID: 18511702.

5. Melani, AS, Bonavia M, Cilenti V, Cinti C, Lodi M, Martucci P, Serra M, Scichilone N, Sestini P, Aliani M, Neri M, on behalf of the Gruppo Educazionale Associazione Italiana Penumologi Ospedalieri (AIPO). Inhaler mishandling remains common in real life and is associated with reduced disease control. Respir Med 2011;105:930-938. PMID: 21367593.

6. Dolovich MB, Ahrens RC, Hess DR, Anderson P, Dhand R, Rau JL, Smaldone GC, Guyatt G. Device selection and outcomes of aerosol therapy: Evidence-based guidelines: American College of Chest Physicians/American College of Asthma, Allergy, and Immunology Chest. 2005;127:335-71. PMID: 21367593.

7. The Global initiative for Chronic Obstructive Lung Disease (GOLD) Guidelines: Available at: http://www.goldcopd.com/. Accessed June 6, 2012.

8. Press VG, Shah LM, Lewis S, Ivy K, Charbeneau J, Mazurek A, Badlani S, Naureckas E, Krishnan JA. Misuse of respiratory inhalers in hospitalized patients with asthma or COPD. J Gen Intern Med 2012;26(6), 635-642. PMID: 21249463 PMCID: PMC3101982.

9. Cohen DJ, Clark EC, Lawson PJ, Casucci BA, Flocke SA. Identifying teachable moments for health behavior counseling in primary care. Patient Educ Couns 2011;85:e8-e15. PMID: 21183305

10. Press VG, Arora VM, Lewis SL, Charbeneau J, Starkey J, Naureckas ET, Krishnan JA. Teaching the use of respiratory inhalers to hospitalized patients with asthma or COPD: A randomized trial (TURI). J Gen Intern Med May 2012. [Epub ahead of print.] PMID: 22592354.

11. Paasche-Orlow MK, Riekert KA, Bilderback A, Chanmugam A, Hill P, Rand CS. Tailored education may reduce health literacy disparities in asthma self-management. Am J Respir Crit Care Med 2005;172(8):980-986. PMID: 16081544 PMCID: PMC2718412.

12. Virchow JC, Crompton GK, Negro RD, Pedesen S, Magnan A, Seidenberg J, Barnes PJ. Importance of inhaler devices in the management of airway disease. Respir Med 2008;102:10-19. PMID: 17923402.

13. Jack BW, Chetty VK, Anthony D, Greenwalk JL, Sanchez GM, Johnson AE, Forsythe SR, O'Donnell JK, Paasche-Orlow MK, Manasseh C, Martin S, Culpepper L. A Reengineered Hospital Discharge Program to Decrease Rehospitalization. A Randomized Trial. Ann Intern Med 2009;150(3):178:187. PMID: 19189907. PMCID: PMC2738592.

14. Gerber BS, Brodsky IG, Lawless KA, Smolin LI, Arozullah AM, Smith EV, Berbaum ML, Heckerling PS, Eiser AR. Implementation and evaluation of a low-literacy diabetes education computer multimedia application. Diabetes Care 2005;28(7):1574-1580. PMID: 15983303.

15. Press VG, Shah LM, Lewis SL, Ivy K, Charbeneau JT, Mazurek A, Krishnan JA. Teach-to-goal intervention to teach respiratory inhaler technique in patients hospitalized for asthma or COPD. Abstract, accepted for poster presentation, Society of General Internal Medicine Annual Meeting May 2009.

16. Davis SQ, Permutt Z, Permutt S, Naureckas ET, Bilderback AL, Rand CS, Stein BD, Krishnan JA. Perception of airflow obstruction in patients hospitalized for acute asthma. *Annals of Asthma, Allergy, and Immunology.* 2009*;102(6)455-61.*

17. Hedeker D, Gibbons RD. Longitudinal Data Analysis. New Jersey: John Wiley & Sons; 2006.

18. Nurss JR, Parker RM, Williams MV, Baker, DW. Short Test of Functional Health Literacy in Adults (STOFHLA). Hartford: Peppercorn Books & Press; 2001.

19. Snellen Eye Chart: A Description and Explanation. Available at: http://precision-vision.com/index.cfm/feature/24_149/snellen-eye-chart---a-description-and-explanation.cfm. Accessed June 6, 2012.

20. Kendrick KR, Baxi SC, Smith RM. Usefulness of the modified 0-10 Borg scale in assessing the degree of dyspnea in patients with COPD and asthma. J Emerg Nurs 2002;26:216-222. PMID: 10839848.

21. Eisner MD, Trupin L, Katz PP, Yelin EH, Earnest G, Balmes J, Blanc PD. Development and validation of a survey-based COPD severity score. Chest 2005;127:1890-1897. PMID: 15947299.

22. Hung HMJ, Wang SJ, O'Neill R. A regulatory perspective on choice of margin and statistical inference issue in non-inferiority trials. Biom J 2005;47(1):28-36. PMID: 16395994.

Protocol Title: Video vs. TTG Respiratory inhaler technique Assessment and InstructioN (V-TRaIN)

University of Chicago
PI: Valerie G. Press, MD, MPH

Co-Investigators: David Meltzer, MD, PhD, Vineet Arora, MD, MAPP, Ted Naureckas, MD

Research personnel: Kristin Constantine, Nicole Babuskow, Danielle Blazek, Lisa Spampinato, Laura Ruth Venable

BACKGROUND

Asthma and Chronic obstructive pulmonary disease (COPD) results in over a million hospitalizations in the United States annually[1] and COPD is the third leading cause of 30-day re-hospitalizations.[2] Clinical trials have established the efficacy of treatments primarily dispensed via respiratory inhaler devices that reduce morbidity and health care utilization if they are used correctly.[3-4] Unfortunately, the effectiveness of these medications in real-world settings is limited by the fact that patients often do not use inhalers correctly.[5-6] Current guidelines recommend assessing and teaching inhaler technique at all health care encounters, including hospitalization.[77.5] My work has found that over 75% of hospitalized patients in an urban, predominantly underserved population misuse their respiratory inhalers, highlighting a missed opportunity to educate these patients with high potential to benefit.[8] Hospitalization, therefore, provides a potential ‘teachable moment' to correct this misuse.[9] My preliminary data indicate that one strategy, in-person teach-to-goal (TTG), is effective in teaching hospitalized patients proper inhaler technique and is more effective than simple verbal instruction.[10]

While TTG is a promising method to improve care for patients who use inhalers, several limitations prevent widespread adoption. First, TTG relies on in-person assessment and education, as well as training and monitoring instructors to ensure fidelity, making it time-consuming and costly. Also, because a single educational session does not ensure long-term retention,[11-12] post-discharge reinforcement may be needed, which may be impractical with in-person TTG. One potential method to surmount TTG's limitations is use of interactive video module education (VME), a method that has been used for health education in other clinical contexts.[13-14] Through iterative self-assessments and video-demonstrations on a tablet computer, VME has the potential to be less costly, maintain fidelity, and be more easily extended into the post-discharge setting than in-person TTG. However, certain questions remain about VME. It is unclear whether VME will yield similar results when compared to TTG, or whether urban, underserved patients will have the ability to, and be willing to use, VME in the post-discharge setting. Therefore, before widespread implementation of VME, it is critical to rigorously develop and test VME for inhaler education in the hospital setting. Ultimately, it will also be important to understand patients' ability and willingness to use post-discharge VME for educational reinforcement to allow for this strategy to transition patients across care settings from hospital to home. Through these funded projects, I will acquire critical data I need to develop R-01 studies focused on improving self-management for patients with obstructive pulmonary disease across care transitions.

<u>We hypothesize that interactive VME will lead to non-inferior rates of ability to demonstrate correct inhaler use compared to rates with TTG among hospitalized patients with Asthma or COPD.</u>

The specific aims to test this hypothesis are:

1. **To develop and iteratively test VME to teach correct use of metered dose inhaler (MDIs) and dry-powder Diskus® devices to hospitalized patients with Asthma or COPD.**

   We will partner with Click to Play Media™ (C2P) to develop VME modules that (self)-assess and teach respiratory inhaler technique to hospitalized participants. Once the VME is developed we will iteratively pilot test (n=30) the VME strategy (up to 3 rounds) to obtain important patient feedback and preferences for the module.

2. **To determine if the proportion of hospitalized patients with Asthma or COPD who demonstrate correct inhaler technique after receiving VME is not significantly less than (non-inferior to) the proportion who demonstrate correct inhaler technique after receiving in-person TTG.**

   To accomplish this aim we will build on our prior work with developing and evaluating the in-person TTG strategy to develop a parallel VME strategy that incorporates guideline-recommended components of assessment and instruction. We will conduct a behavioral randomized clinical trial to evaluate the relative effectiveness and durability of VME versus in-person TTG education on the ability of hospitalized patients to demonstrate correct MDI and Diskus® use. Secondarily, we will assess whether there is variation in comparative effectiveness by patient characteristics including age, gender, and health literacy level, among other important factors.

OVERVIEW OF TRIAL DESIGN

This will be a randomized, controlled, non-inferiority clinical trial conducted of patients admitted to the University of Chicago. Adults admitted with a history of Asthma or COPD will be eligible to participate in this study. Upon receiving consent, participants will be randomized to either the VME or TTG. Inhaler technique will be measured prior to hospital discharge before and after education (VME or TTG). This will provide data on the short-term effectiveness of the interventions. Retention will be examined at 30 days after hospital discharge.

ELIGIBILITY CRITERIA

The goal of patient selection is to enroll adults admitted with a history of physician-diagnosed Asthma or COPD.

Inclusion criteria

1. Age 18 years and older
2. Admission to the inpatient medical service and surgical service
3. Physician-diagnosed Asthma or COPD. We will enroll patients even if the primary reason for admission is not Asthma or COPD (e.g., patients admitted for heart failure, but with a physician diagnosis of COPD are eligible).
4. Physician plans to discharge patients home a metered dose inhaler (MDI; e.g., albuterol)

Exclusion criteria

1. Currently in an intensive care unit
2. Physician declines to provide consent
3. Patient unable to provide consent (e.g., history of cognitive impairment, unable to understand English) or declines to provide consent
4. Previous participant in this study

RECRUITMENT

We will employ the same recruitment plan as in the protocol for our Effectiveness of interventions to Teach Respiratory Inhaler Technique (E-TRaIN) study (Protocol #11-0248). In summary, admission logs (EPIC) at the University of Chicago will be examined each day to screen for potentially eligible patients. The treating physician will be contacted for verbal assent using standardized text. Among patients whose physician provides assent, medical records will be reviewed to ascertain eligibility and informed consent will be sought from eligible patients. A total of 142 patients will be recruited at the University of Chicago Medical Center over 15 months.

TREATMENT CONDITIONS

1. **Teach-to-Goal (TTG):** During inpatient admission, participants assigned to the TTG conditon will be provided with an intensive, iterative education and evaluation strategy that consists of the following steps: First, participants will undergo an initial assessment of respiratory inhaler technique for MDI (and a Diskus® device if they are also prescribed medication using the device, i.e., fluticasone/salmeterol [Advair Diskus ®]) using standardized device-specific checklists (Form 1) that we have developed and used in the SARI study (Protocol #15729A), in TURI (#16900A), and in E-TRaIN (#11-0248). This will be followed by the educational intervention whereby participants will then be taught using verbal instructions and then by demonstration by a trained educator regarding the appropriate use of the MDI (and, if applicable, fluticasone/salmeterol (Advair Diskus®) using standardized written instructions (Forms 2a and 2b). Next the participants will be asked to demonstrate how they use their respiratory inhalers; their post-TTG technique will be graded using the same checklists as the initial assessment. The participants will receive additional rounds of instruction following this assessment if needed (e.g., if the participant did not achieve mastery of the MDI or Diskus® after one round of teaching). Although initial pilot work showed that two rounds was sufficient, more recent data indicates that some participants require > 2 rounds; since the essence of this education technique is to teach-to-goal, and this requires only a few additional minutes (maxium) we will attempt to teach-to-goal. The patient's hospital physician (attending physician or their designee) will be provided information about the patients inhaler technique for both inhalers after education; this will provide an opportunity for the patient to receive addition instructions by the clinical team if needed. All instructions will be provided by a dedicated trained educator; all assessments will be performed by a second research assisstant (trained assessor). The trained assessor will be masked to treatment assignment (see below).[15]
2. **Video Module Education (VME):** The RE will provide participants with a tablet device and will demonstrate how to access VME and complete the pre/post e-learning assessments. The RE will provide technical support but will neither participate directly in the education nor help with the self-assessments. The participants will first complete the pre-assessment e-learning tool on the tablet. They will then watch the video instruction that will provide a complete demonstration with verbal instructions on correct inhaler technique. Next the participants will complete the post-assessment e-learning tool. Based on participants' performance, they will be directed to further tailored video-instruction. The cycle of self-assessment and video instruction will continue until sufficient mastery has been achieved.

Other considerations:

1. *Masking:* To minimize risk of biased measurements of inhaler technique, the trained assessor will be masked to the treatment condition. Specifically, the trained assessor will not be informed about the treatment assignment, will remain outside the patients' room during the educational intervention (and the room door will remain closed during the intervention), and the trained educator will be instructed not to inform the assessor about the treatment condition. We have successfully used similar procedures for masking the trained assessor in other IRB-approved studies (see E-TRaIN, IRB # 11-0248).

2. *Patients being discharged home on a MDI and a Diskus will receive VME or TTG interventions for both devices.* If randomized to the TTG intervention, then TTG will be used to teach MDI and Diskus use; likewise if randomized to the VME intervention, then VME will be used to teach MDI and Diskus use. Based on results of screening data from the TURI study (IRB #16900A), we expect that about 10-20% of participants (10 to 20 participants enrolled in this study) will be prescribed both devices. We would like to include this option to teach two different devices (in patients prescribed both devices) to collect preliminary data about the feasibility of VME for MDI vs. TTG for Diskus; these data would be valuable as we prepare for future studies.

PROTOCOL FOR BOTH TREATMENT GROUPS:

Following the screening procedures above, we will use the following steps:

1. **Obtain assent from treating physician prior to approaching patients hospitalized with asthma or COPD.** The treating physician will be contacted by a research assistant for verbal assent using standardized text (Form 3).

2. **Obtain written informed consent from patient using standardized text.** One hundred eighteen participants (N=59 TTG condition; N=59 VME condition) will be enrolled in this study.

3. **Optimize room conditions**
   - Room well lit (turn on overhead lights if necessary)
   - Patient preferably sitting up
   - Private setting (close door to patient room)
   - Interviewer should directly face patient
   - Patient should use reading glasses if they use one

4. **Assess patient's respiratory symptoms/morbidity (<u>Forms 4-5-6</u>), which includes the** Borg symptom score, Asthma Symptom Severity Index (ASSI), Chronic Bronchitis Symptom Questionnaire, COPD Severity Score (CSS), Airway Questionnaire (AQ-20), COPD Helplessness Index (CHI) and demographic and other clinical information.

5. **Assess patients' self-efficacy regarding respiratory inhaler use (Form 7)**

6. **Assess lung function. Perform spirometry pre and post-bronchodilator (after 2 puffs albuterol MDI via spacer) using portable KoKo spirometer. (Forms 8a and**

8b). These data will provide an objective measure of disease severity at the time of the hospitalization. We have previously demonstrated that this population has poor perception of airflow obstruction and spirometry is feasible during the inpatient setting.[16]

7. **Assess patient's inhaler technique using Inhaler checklists (Form 1;** for a MDI and, if applicable, Diskus®) by the trained assessor.

8. **Assess health literacy. Check vision (Form 9).** Administer STOHFLA questionnaire (Form 10) if the patient's visual acuity is at least 20/50 in one eye (Check the patient's visual acuity in each eye using the Snellen Pocket Eye Chart (Form 11), otherwise, the patient is ineligible to complete the HL evaluation. Explain the purpose of this survey. Use the following text.

   - We want to find out what health care information is understood by patients and what is confusing to them, in order to help us write things more clearly.
   - Here are some medical instructions that you or anybody might see around the hospital. These instructions are in sentences that have some of the words missing.
   - Where a word is missing, a blank line is drawn, and 4 possible words that could go in the blank appear just below it. I want you to figure out which of those 4 words should go in the blank, which word makes the sentence make sense.
   - When you think you know which one it is, circle the letter in front of that word, and go on to the next one.
   - When you finish the page, turn the page and keep going until you finish all the pages.
   - Provide the patient a pen and a clipboard and ask them to complete the questionnaire (Form 10). If the patients ask for help with completing the questionnaire, instruct them to do the best they can and that you are not allowed to assist them. Only responses completed within 7 minutes are used in calculating a health literacy score. Record the start and stop time and any questions that were answered after 7 minutes had elapsed. Do not grade the STOFHLA until after the study visit is complete.

9. **Obtain randomization assignment from the study biostatistician.** Randomization will be block-stratified by level of health literacy to ensure an equal number of subjects within each stratum assigned to TTG and VME Patients will then receive either the TTG or VME intervention by the trained educator.

10. **Collect patient contact information and provide patient $25 in cash for participating in this study as well as parking pass/bus pass for their follow-up visit.** Record patient's social security number and obtain patients signature that they received the patient incentive.

    1. **Provide patients an appointment for an in-person interview 30 days after discharge (patients will be given a letter with their appointment time).** At

the in-person interview, we will ask patients to complete a short interviewer-administered questionnaire about patient's respiratory symptoms/morbidity/self-reported ability to use MDI and Diskus® inhalers, quality of life (Forms 4 and 12). We will also assess lung function (spirometry) and use of inhalers (MDI and/or Diskus). Upon completing this portion the participant will receive $50 as well as parking pass/bus pass for their next follow-up visit.

OUTCOMES/MEASURES

The primary and secondary outcomes are listed below. Measures for each are described. Initial measurements (Vo; see Data Collection Schedule below) will be made prior to randomization; at V1, the trained educator will assess respiratory inhaler technique by asking the subject to demonstrate use of the MDI (and Diskus® if to be discharged home on fluticasone/salmeterol). Follow-up assessment, V2, will take place at 30 days (+/-7 days) post-hospital discharge.

Primary outcome

The primary outcome will be inhaler technique. We will define inhaler technique in two ways:

a) Correct Use (i.e., >75% of steps correct)
b) Mastery (i.e., perfect technique, 100% steps correct)

This outcome will be measured at V1 and V2. We will test our hypothesis that patients will have a reduced proportion of respiratory events when taught effective inhaler technique with TTG compared to BI education separately for the MDI and Diskus® devices after hospital discharge. We will employ the same checklists used in the E-TRaIN study (IRB Protocol #11-0248, high reliability, kappa >0.94). These checklists will be used to measure the primary outcome: ability to use inhalers (defined as (a) correct use of inhaler: >9/12 steps for MDI; >8/10 steps for Diskus®; and (b) mastery of inhaler technique, i.e., perfect technique: 12/12 steps for MDI; 10/10 steps for Diskus®).

The **primary outcome** is the proportion of participants with MDI misuse post VME vs. TTG education. This will provide data on the short-term effectiveness of the interventions. Longer-term retention (durability) will be examined 30 days post-hospital discharge using an intention-to-treat analysis. We have powered on this binary variable. [Table 1] However, because we recognize that there is more information about which steps are less critical and those that are more critical, I will also work with Robert Gibbons, a nationally recognized expert in biostatistics, to model a mixed effects ordinal regression model with random intercepts and time effects within correlation of each participant with terms for mode (VME and TTG), as well as interaction with time, to determine the predicted probability in each category for VME and TTG of how prevalence of misuse changes over time.[17] **Mediators include** health literacy[18] and vision level[19] as I have previously identified them as important mediators of baseline inhaler misuse and/or ability to learn correct technique.[8,10]

Secondary outcomes

Secondary outcomes will include:

a) Change in self-efficacy for respiratory inhalers (i.e., V0-V2). We will ask all patients to state how strongly they agree or disagree with the following statement "I am confident that I know how to use [insert inhaler name] correctly" for both MDI and Diskus®. The subject will be shown an example of each inhaler. Each item will be scored on a 5-point Likert scale (strongly disagree to strongly agree); the mean score will be used to

measure self-efficacy. Cronbach's alpha will be calculated to evaluate internal consistency.

b) Symptom burden (i.e., BORG, ASSI, and CBSQ) at V1 and V2. Patient reported outcomes will be measured using a modified Borg dyspnea scale (Borg), Asthma Symptom Severity Index (ASSI), Chronic Bronchitis Symptom Questionnaire (CBSQ).

c) Acute care utilization for respiratory exacerbations at V2. Data will be collected using interviewer administrated surveys. *Respiratory events* are defined as: physician, emergency room or hospital visit, or death. This composite endpoint is due to small sample size.

The comparative effectiveness of TTG versus VME with regards to these secondary outcomes will employ simple univariate and/or multivariate logistic regression, generalized estimating equations, or generalized non-linear mixed models, as appropriate. For each of these outcomes, we will evaluate whether health literacy level modifies the effectiveness of VME (Vs. TTG) by including an interaction term in the regression model. Model assumptions will be checked. Use of two inhalers will also provide the opportunity to evaluate if the comparative effectiveness of VME (vs. TTG) varies by device. A two-tailed p-value less than 0.05 will define statistical significance. The sample size calculations demonstrate that N=59 per group will provide 80% power for an intention-to-treat analysis to test for non-inferiority of VME vs. TTG.

Secondary Outcomes include quality of life (QOL),[20] patient reported outcomes,[20-21] lung function, utilization of health care services (outpatient visits, ED and hospital admissions, deaths), and self-efficacy of inhaler technique.[8,10] We will also evaluate the differential effects of VME vs. TTG by participant characteristics (e.g., age, health literacy). Finally we will evaluate the preliminary comparative durability of VME vs. TTG at 30 days post-hospital discharge. We will perform all tests separately for both MDI and Diskus devices.

DATA COLLECTION SCHEDULE

	**Inpatient Visit**		**30 day FU**
**VISIT**	**V0-V1**		**V2**
Visit duration (minutes)	60 (total for V0 to V1)		60
Visit location	Inpatient		Outpatient
Visit type	In-person		In-person
Eligibility evaluation	■		
Consent	■		
Randomization	■		
Self Efficacy: Self-reported inhaler technique	■		■
Patient demonstration of inhaler technique	■	■	■
Vision Screen			
Health Literacy	■		
Spirometry before and after bronchodilator	■		■
Review of medications	■		■

Medication count/weigh			■
Questionnaire about symptom burden, respiratory exacerbations, acute care utilization	■		■
Discharge Questionnaire		■	

Vo: initial assessment V1: after instruction V2: 30 days post-discharge follow-up

This study will require 24 months:

1. Develop/pilot test interactive video (6 month)
2. Training of personnel (1 month)
3. Begin recruitment (1-3 participants/week, for 15 months)
4. Complete follow-up of last enrollee (1 month after last enrolled participant)
5. Data analyses and preparation of final report (2 months).

The research will take place on the inpatient wards of UCMC.

ANALYSES

Analysis plan. We will use means, medians, proportions, scatterplots, and histograms to describe the data. We will test for group differences using the *t*-test, the Wilcoxon rank sum test, Pearson's chi-squared test, or Fisher's exact tests as appropriate. We will adhere to the intention-to-treat principle when testing for differences by intervention type. The **primary outcome** will be correct inhaler technique; chi-squared tests will be used to compare post VME vs. post TTG prevalence of inhaler misuse (<75% of steps correct). **Secondary outcomes** will include change in self-efficacy for respiratory inhalers, symptom burden (i.e., BORG and CSS), self-reported utilization of health care services for respiratory exacerbations, basic disease knowledge, and QOL. The comparative effectiveness of VME versus TTG with regards to these secondary outcomes will employ simple univariate and/or multivariate logistic regression, as appropriate. For each of these outcomes, we will evaluate whether patient characteristics (vision, age, and/or health literacy level) modifies the effectiveness of VME versus TTG by evaluating interactions in the regression model. A two-tailed p-value less than 0.05 will define statistical significance. The sample size calculations (see below) demonstrate that n=59 per group will provide 80% power for an intention-to-treat analysis.

Sample size. (Table 1) The main outcome is correct MDI use (>75% steps correct). In my preliminary studies above, the proportion of participants with correct MDI use after TTG was 88-100% (n=98, n=50, respectively).[8,10] These data (expected proportion of correct MDI use of 0.95), along with a noninferiority effect size of -0.10, were used to calculate the sample size.[22] We will enroll 142 participants over 15 months and will have sufficient power (0.8) to determine if VME is non-inferior to TTG, at a significance level of p<0.05. We have experience recruiting participants and have been able to recruit up to 12 participants per month; therefore, we do not foresee any problems with recruiting this number of participants in this timeline.

Table 1: Sample Size per intervention (VME/TTG)

	**Power**				
**EFFECT SIZE**	**0.60**	**0.70**	**0.80**	**0.90**	**0.95**
**-0.05**	137	179	236	326	412
**-0.10**	35	45	59	82	103
**-0.15**	16	20	27	37	46

<u>Interim analyses:</u> After 50% of the target sample size have completed the study (59 participants have completed the study) or after 50% of the study period (~7 months), whichever occurs first, results of an interim analysis will be provided to a Data Safety and Monitoring Board (Drs. Dana

Edelson, MD; Steve White, MD; Neda Laiteerapong, MD; physicians who are not study investigators). Investigators and research assistants will remain masked to the results of the interim analyses until the end of the study. The DSMB will be asked to consider issues related to study performance (enrollment rates, study completion rates) and differences in outcomes between groups and provide recommendations to the PI and the IRB whether to continue the study or terminate enrollment.

HUMAN SUBJECTS

We are not aware of any potential benefits to study subjects. Potential risks include:

1. The subject may be uncomfortable answering some interview questions. They can refuse to answer any question.

2. The lung function test is safe and is commonly used to measure the severity of COPD but can cause some minor chest soreness or lightheadedness; study test will be done by a trained member of the research staff with the subject in a seated position. To further minimize the risk of adverse events with spirometry, we will not perform spirometry in subjects with SBP>180 or DBP>100 mm Hg.

3. Two puffs of albuterol (for post-bronchodilator spirometry) is generally well tolerated. We have not had adverse events with 2 puffs albuterol after enrolling >170 subjects in the SARI, COAT, and TURI studies (IRBs# 15729A, 14831A, 16900A). In some cases, however, 2 puffs of albuterol can cause jitteriness, anxiety, and tachyarrhythmias. To minimize this risk, we will withhold albuterol in subjects who are hospitalized with tachyarrhythmias or palpitations or report problems after using albuterol.

4. Long acting beta-2-agonists (salmeterol or formoterol). These medications are generally well-tolerated, but can have albuterol-like adverse effects; in addition, these agents have been associated with increase risk of asthma-related deaths.

5. Inhaled corticosteroids (fluticasone/budesonide). These medications are well tolerated, but can cause thrush, dysphonia, and other less common adverse effects.

6. Loss of confidentiality. To help ensure that patients' health information remains private, we will restrict access to data collected for our study to study personnel only (via use of password-protection and locked cabinets for study documents). Study ID numbers will be generated and will be used when discussing and/or reviewing data at study meetings. Also, study reports will report results in aggregate and not contain information that can be used to identify individual patients.

Data Safety Monitoring Plan

The PI will review monthly data reports reviewing recruitment, consent, intervention activities, adverse events, and follow-up. Although few, if any, intervention-related adverse events are anticipated in this education-related study, the study population is at risk for poor outcomes, including life-threatening exacerbations and death. The PI will comply with prompt reporting of any unanticipated events and the Data Safety and Monitoring Board (DSMB) (Dana Edelson, MD, Steve White, MD, Neda Laiteerapong, MD). The DSMB (see interim analysis above) will also review results of the interim and final analyses.

PATIENT INCENTIVES

Participants will be provided $25 for completing the initial baseline interview and $50 completing the post-hospital discharge in-person interview. Total possible monetary compensation is $75. Participants will also be provided parking passes or bus passes for their follow-up visits as we recognize that this cost burden should not fall on the participant for participating in our study. All tests for this study will be done free of charge. Monetary compensation will be reduced, based on the extent to which the participante completes the study.

BIBLIOGRAPHY

1. Morbidity and Mortality 2007 Chart Book on Cardiovascular, Lung, and Blood diseases. Available at: http://www.nhlbi.nih.gov/resources/docs/07-chtbk.pdf. Accessed June 06, 2012.

2. Jencks SF, Williams MV, Coleman EA. Rehospitalizations among patients in the Medicare fee-for-service program. N Engl J Med 2009;360(14):1418-1428. PMID: 19339721.

3. Hanania NA, Darken P, Horstman D, Reisner C, Lee B, Davis S, Shah T. The efficacy and safety of Fluticasone Propionate (250ug) /Salmeterol (50ug) combined in the Diskus® Inhaler for the treatment of COPD. Chest 2003;124:834-843. PMID: 12970006.

4. Celli BR, Thomas NE, Anderson JA, Ferguson GT, Jenkins CR, Jones PW, Vestbo J, Knobil K, Yates JC, Calverley PMA. Effect of pharmacotherapy on rate of decline of lung function in chronic obstructive pulmonary disease. Results from the TORCH Study. Am J Resp Crit Care Med 2008;178:332-338. PMID: 18511702.

5. Melani, AS, Bonavia M, Cilenti V, Cinti C, Lodi M, Martucci P, Serra M, Scichilone N, Sestini P, Aliani M, Neri M, on behalf of the Gruppo Educazionale Associazione Italiana Penumologi Ospedalieri (AIPO). Inhaler mishandling remains common in real life and is associated with reduced disease control. Respir Med 2011;105:930-938. PMID: 21367593.

6. Dolovich MB, Ahrens RC, Hess DR, Anderson P, Dhand R, Rau JL, Smaldone GC, Guyatt G. Device selection and outcomes of aerosol therapy: Evidence-based guidelines: American College of Chest Physicians/American College of Asthma, Allergy, and Immunology Chest. 2005;127:335-71. PMID: 21367593.

7. The Global initiative for Chronic Obstructive Lung Disease (GOLD) Guidelines: Available at: http://www.goldcopd.com/. Accessed June 6, 2012.

7.5 Guidelines for the Diagnosis and Management of Asthma (EPR-3). Available at: https://www.nhlbi.nih.gov/guidelines/asthma/. Accessed June 6, 2012.

8. Press VG, Shah LM, Lewis S, Ivy K, Charbeneau J, Mazurek A, Badlani S, Naureckas E, Krishnan JA. Misuse of respiratory inhalers in hospitalized patients with asthma or COPD. J Gen Intern Med 2012;26(6), 635-642. PMID: 21249463 PMCID: PMC3101982.

9. Cohen DJ, Clark EC, Lawson PJ, Casucci BA, Flocke SA. Identifying teachable moments for health behavior counseling in primary care. Patient Educ Couns 2011;85:e8-e15. PMID: 21183305

10. Press VG, Arora VM, Lewis SL, Charbeneau J, Starkey J, Naureckas ET, Krishnan JA. Teaching the use of respiratory inhalers to hospitalized patients with asthma or COPD: A randomized trial (TURI). J Gen Intern Med May 2012. [Epub ahead of print.] PMID: 22592354.

11. Paasche-Orlow MK, Riekert KA, Bilderback A, Chanmugam A, Hill P, Rand CS. Tailored education may reduce health literacy disparities in asthma self-management. Am J Respir Crit Care Med 2005;172(8):980-986. PMID: 16081544 PMCID: PMC2718412.

12. Virchow JC, Crompton GK, Negro RD, Pedesen S, Magnan A, Seidenberg J, Barnes PJ. Importance of inhaler devices in the management of airway disease. Respir Med 2008;102:10-19. PMID: 17923402.

13. Jack BW, Chetty VK, Anthony D, Greenwalk JL, Sanchez GM, Johnson AE, Forsythe SR, O’Donnell JK, Paasche-Orlow MK, Manasseh C, Martin S, Culpepper L. A Reengineered Hospital Discharge Program to Decrease Rehospitalization. A Randomized Trial. Ann Intern Med 2009;150(3):178:187. PMID: 19189907. PMCID: PMC2738592.

14. Gerber BS, Brodsky IG, Lawless KA, Smolin LI, Arozullah AM, Smith EV, Berbaum ML, Heckerling PS, Eiser AR. Implementation and evaluation of a low-literacy diabetes education computer multimedia application. Diabetes Care 2005;28(7):1574-1580. PMID: 15983303.

15. Press VG, Shah LM, Lewis SL, Ivy K, Charbeneau JT, Mazurek A, Krishnan JA. Teach-to-goal intervention to teach respiratory inhaler technique in patients hospitalized for asthma or COPD. Abstract, accepted for poster presentation, Society of General Internal Medicine Annual Meeting May 2009.

16. Davis SQ, Permutt Z, Permutt S, Naureckas ET, Bilderback AL, Rand CS, Stein BD, Krishnan JA. Perception of airflow obstruction in patients hospitalized for acute asthma. *Annals of Asthma, Allergy, and Immunology.* 2009*;102(6)455-61.*

17. Hedeker D, Gibbons RD. Longitudinal Data Analysis. New Jersey: John Wiley & Sons; 2006.

18. Nurss JR, Parker RM, Williams MV, Baker, DW. Short Test of Functional Health Literacy in Adults (STOFHLA). Hartford: Peppercorn Books & Press; 2001.

19. Snellen Eye Chart: A Description and Explanation. Available at: http://precision-vision.com/index.cfm/feature/24_149/snellen-eye-chart---a-description-and-explanation.cfm. Accessed June 6, 2012.

20. Kendrick KR, Baxi SC, Smith RM. Usefulness of the modified 0-10 Borg scale in assessing the degree of dyspnea in patients with COPD and asthma. J Emerg Nurs 2002;26:216-222. PMID: 10839848.

21. Eisner MD, Trupin L, Katz PP, Yelin EH, Earnest G, Balmes J, Blanc PD. Development and validation of a survey-based COPD severity score. Chest 2005;127:1890-1897. PMID: 15947299.

22. Hung HMJ, Wang SJ, O'Neill R. A regulatory perspective on choice of margin and statistical inference issue in non-inferiority trials. Biom J 2005;47(1):28-36. PMID: 16395994.

Protocol Title: Video vs. TTG Respiratory inhaler technique Assessment and InstructioN (V-TRaIN)

University of Chicago
PI: Valerie G. Press, MD, MPH

Co-Investigators: David Meltzer, MD, PhD, Vineet Arora, MD, MAPP, Ted Naureckas, MD

Research personnel: Kristin Constantine, Nicole Babuskow, Danielle Blazek, Lisa Spampinato, Laura Ruth Venable

BACKGROUND

Asthma and Chronic obstructive pulmonary disease (COPD) results in over a million hospitalizations in the United States annually[1] and COPD is the third leading cause of 30-day re-hospitalizations.[2] Clinical trials have established the efficacy of treatments primarily dispensed via respiratory inhaler devices that reduce morbidity and health care utilization if they are used correctly.[3-4] Unfortunately, the effectiveness of these medications in real-world settings is limited by the fact that patients often do not use inhalers correctly.[5-6] Current guidelines recommend assessing and teaching inhaler technique at all health care encounters, including hospitalization.[77.5] My work has found that over 75% of hospitalized patients in an urban, predominantly underserved population misuse their respiratory inhalers, highlighting a missed opportunity to educate these patients with high potential to benefit.[8] Hospitalization, therefore, provides a potential 'teachable moment' to correct this misuse.[9] My preliminary data indicate that one strategy, in-person teach-to-goal (TTG), is effective in teaching hospitalized patients proper inhaler technique and is more effective than simple verbal instruction.[10]

While TTG is a promising method to improve care for patients who use inhalers, several limitations prevent widespread adoption. First, TTG relies on in-person assessment and education, as well as training and monitoring instructors to ensure fidelity, making it time-consuming and costly. Also, because a single educational session does not ensure long-term retention,[11-12] post-discharge reinforcement may be needed, which may be impractical with in-person TTG. One potential method to surmount TTG's limitations is use of interactive video module education (VME), a method that has been used for health education in other clinical contexts.[13-14] Through iterative self-assessments and video-demonstrations on a tablet computer, VME has the potential to be less costly, maintain fidelity, and be more easily extended into the post-discharge setting than in-person TTG. However, certain questions remain about VME. It is unclear whether VME will yield similar results when compared to TTG, or whether urban, underserved patients will have the ability to, and be willing to use, VME in the post-discharge setting. Therefore, before widespread implementation of VME, it is critical to rigorously develop and test VME for inhaler education in the hospital setting. Ultimately, it will also be important to understand patients' ability and willingness to use post-discharge VME for educational reinforcement to allow for this strategy to transition patients across care settings from hospital to home. Through these funded projects, I will acquire critical data I need to develop R-01 studies focused on improving self-management for patients with obstructive pulmonary disease across care transitions.

<u>We hypothesize that interactive VME will lead to non-inferior rates of ability to demonstrate correct inhaler use compared to rates with TTG among hospitalized patients with Asthma or COPD.</u>

The specific aims to test this hypothesis are:

1. **To develop and iteratively test VME to teach correct use of metered dose inhaler (MDIs) and dry-powder Diskus® devices to hospitalized patients with Asthma or COPD.**

   We partnered with Click to Play Media™ (C2P) and Smart Sparrow to develop VME modules that (self)-assess and teach respiratory inhaler technique to hospitalized participants. We have tested the VME in focus groups (IRB #13-1139). Now that the VME is developed we will iteratively test (n=30-40) the VME strategy (up to 3 rounds (90-120 total)) to obtain important patient feedback, preferences and preliminary efficacy estimates for the module.

   We hypothesize that participants' post-VME inhaler technique will be significantly better than their pre-VME inhaler technique.

2. **To determine if the proportion of hospitalized patients with Asthma or COPD who demonstrate correct inhaler technique after receiving VME is not significantly less than (non-inferior to) the proportion who demonstrate correct inhaler technique after receiving in-person TTG.**

   To accomplish this aim we will build on our prior work with developing and evaluating the in-person TTG strategy to develop a parallel VME strategy that incorporates guideline-recommended components of assessment and instruction. We will conduct a behavioral randomized clinical trial to evaluate the relative effectiveness and durability of VME versus in-person TTG education on the ability of hospitalized patients to demonstrate correct MDI and Diskus® use. Secondarily, we will assess whether there is variation in comparative effectiveness by patient characteristics including age, gender, and health literacy level, among other important factors.

We hypothesize that interactive VME will lead to non-inferior rates of ability to demonstrate correct inhaler use compared to rates with TTG among hospitalized patients with Asthma or COPD.

SPECIFIC AIM 1

This will be a pre/post study of inpatients admitted to the University of Chicago. Adults admitted with a history of physician diagnosed COPD or asthma will be eligible to participate in this study. Upon receiving consent, participants will enrolled. Inhaler technique will be measured pre and post VME. This will provide data on the short-term efficacy of the VME.

Inclusion criteria:

1. Age 18 years and older
2. Admission to the inpatient medical service and surgical service
3. Physician-diagnosed COPD or asthma. We will enroll patients even if the primary reason for admission is not COPD or asthma (e.g., patients admitted for heart failure, but with a physician diagnosis of COPD are eligible).
4. Physician plans to discharge patients home a metered dose inhaler (MDI; e.g., albuterol)

Exclusion criteria
1. Currently in an intensive care unit
2. Physician declines to provide consent
3. Patient unable to provide consent (e.g., history of cognitive impairment, unable to understand English) or declines to provide consent

RECRUITMENT

Admission logs (EPIC) at the University of Chicago will be examined each day to screen for potentially eligible patients. The treating physician will be contacted for verbal assent using standardized text. Among patients whose physician provides assent, medical records will be reviewed to ascertain eligibility and informed consent will be sought from eligible patients. Sixty to ninety participants (approximately 30 per iterative round of VME up to 3 rounds) will be recruited at the University of Chicago Medicine over 2-6 months.

Video Module Education (VME):

The RE will provide participants with a tablet device and will demonstrate how to access VME and complete the pre/post e-learning assessments. The RE will provide technical support but will neither participate directly in the education nor help with the self-assessments. The participants will first complete the pre-assessment e-learning tool on the tablet. They will then watch the video instruction that will provide a complete demonstration with verbal instructions on correct inhaler technique. Next the participants will complete the post-assessment e-learning tool. Based on participants' performance, they will be directed to further tailored video-instruction. The cycle of self-assessment and video instruction will continue until sufficient mastery has been achieved.

Following the screening procedures above, we will use the following steps:

1. **Obtain assent from treating physician prior to approaching patients hospitalized with asthma or COPD.** The treating physician will be contacted by a research assistant for verbal assent using standardized text (Form 3).

2. **Obtain written informed consent from patient using standardized text.**

3. **Optimize room conditions**
   - Room well lit (turn on overhead lights if necessary)
   - Patient preferably sitting up
   - Private setting (close door to patient room)
   - Interviewer should directly face patient
   - Patient should use reading glasses if they use one

4. **Assess patient's respiratory symptoms/morbidity (Forms 4-5-6), which includes the** Borg symptom score, Asthma Symptom Severity Index (ASSI), Chronic Bronchitis Symptom Questionnaire, COPD Severity Score (CSS), Airway Questionnaire (AQ-20), COPD Helplessness Index (CHI) and demographic and other clinical information.

5. **Assess patients' self-efficacy regarding respiratory inhaler use (Form 7)**

6. **Assess lung function. Perform spirometry pre and post-bronchodilator (after 2 puffs albuterol MDI via spacer) using portable KoKo spirometer. (Forms 8a and 8b).** These data will provide an objective measure of disease severity at the time of the hospitalization. We have previously demonstrated that this population has poor perception of airflow obstruction and spirometry is feasible during the inpatient setting.[16]

7. **Assess patient's inhaler technique using Inhaler checklists (Form 1;** for a MDI) by the trained assessor.

8. **Assess health literacy. Check vision (Form 9). Ask brief health literacy screen questions (Form 10a) for all participants and a**dminister STOHFLA questionnaire (Form 10b) if the patient's visual acuity is at least 20/50 in one eye (Check the patient's visual acuity in each eye using the Snellen Pocket Eye Chart (<u>Form 11</u>), otherwise, the patient is ineligible to complete the STOHFLA HL evaluation (will complete the BHLS questions).

a) **BHLS**

We want to find out what health care information is understood by patients and what is confusing to them, in order to help us understand what patients may find confusing.]

1) How often do you have problems learning about your medical condition because of difficulty understanding written information?
2) How confident are you filling out medical forms by yourself?
3) How often do you have someone help you read hospital material?

b) STOHFLA

Explain the purpose of this survey. Use the following text.

- We want to find out what health care information is understood by patients and what is confusing to them, in order to help us write things more clearly.

- Here are some medical instructions that <u>you or anybody might see</u> around the hospital. These instructions are in sentences that have some of the words missing.

- Where a word is missing, a blank line is drawn, and 4 possible words that could go in the blank appear just below it. I want you to figure out which of those 4 words should go in the blank, which word makes the sentence make sense.

- When you think you know which one it is, circle the letter in front of that word, and go on to the next one.

- When you finish the page, turn the page and keep going until you finish all the pages.

- Provide the patient a pen and a clipboard and ask them to complete the questionnaire (Form 10). If the patients ask for help with completing the questionnaire, instruct them to do the best they can and that you are not allowed to assist them. Only responses completed within 7 minutes are used in calculating a health literacy score. Record the start and stop time and any questions that were

answered after 7 minutes had elapsed. Do not grade the STOFHLA until after the study visit is complete.

9. **Collect patient contact information and provide patient $25 in cash for participating in this study.** Record patient's social security number and obtain patients signature that they received the patient incentive.

OUTCOMES/MEASURES-AIM 1

The primary and secondary outcomes are listed below. Measures for each are described. Initial measurements (Vo; see Data Collection Schedule below) will be made prior to randomization; at V1, the trained educator will assess respiratory inhaler technique by asking the subject to demonstrate use of the MDI.

Primary outcome

The primary outcome will be inhaler technique. We will define inhaler technique in two ways:

a) Correct Use (i.e., >75% of steps correct)
b) Mastery (i.e., perfect technique, 100% steps correct)

This outcome will be measured at V1. We will test our hypothesis that patients will have improved inhaler technique post-VME compared to pre-VME. We will employ the same checklists used in the E-TRaIN study (IRB Protocol #11-0248, high reliability, kappa >0.94). These checklists will be used to measure the primary outcome: ability to use inhalers (defined as (a) correct use of inhaler: >9/12 steps for MDI; and (b) mastery of inhaler technique, i.e., perfect technique: 12/12 steps for MDI).

The **primary outcome** is the proportion of participants with MDI misuse post VME vs. pre VME. This will provide data on the short-term efficacy of VME.

Secondary outcomes

Secondary outcomes will include:

a) <u>Symptom burden (i.e., BORG, ASSI, and CBSQ) at V1 and V2</u>. Patient reported outcomes will be measured using a modified Borg dyspnea scale (Borg), Asthma Symptom Severity Index (ASSI), Chronic Bronchitis Symptom Questionnaire (CBSQ).
b) quality of life (QOL),[20]
c) and self-efficacy of inhaler technique.[8,10]

The efficacy of VME with regards to these secondary outcomes will employ simple univariate and/or multivariate logistic regression, generalized estimating equations, or generalized non-linear mixed models, as appropriate. For each of these outcomes, we will evaluate whether health literacy level modifies the effectiveness of VME by including an interaction term in the regression model. Model assumptions will be checked. A two-tailed p-value less than 0.05 will define statistical significance.

DATA COLLECTION SCHEDULE-AIM 1

VISIT	Inpatient Visit V0-V1	
Visit duration (minutes)	60 (total for V0 to V1)	
Visit location	Inpatient	
Visit type	In-person	
Eligibility evaluation	■	
Consent	■	
Self Efficacy: Self-reported inhaler technique	■	
Patient demonstration of inhaler technique	■	■
Vision Screen		
Health Literacy	■	
Spirometry before and after bronchodilator	■	
Review of medications	■	
Medication count/weigh		
Questionnaire about symptom burden, respiratory exacerbations, acute care utilization	■	
Discharge Questionnaire		■

Vo: initial assessment V1: after instruction V2: 30 days post-discharge follow-up

This study will require 2-6 months:

1. Pilot test interactive video round 1 (2 months)
2. Pilot test interactive video round 2 (2 months) if changes to VME needed
3. Pilot test interactive video round 3 (2 months) if changes to VME needed
4. Data analyses and preparation of final report (2 months).

The research will take place on the inpatient wards of UCM.

ANALYSES-AIM 1

Analysis plan. We will use means, medians, proportions, scatterplots, and histograms to describe the data. We will test for group differences using the *t*-test, the Wilcoxon rank sum test, Pearson's chi-squared test, or Fisher's exact tests as appropriate. We will adhere to the intention-to-treat principle when testing for differences by intervention type. The **primary outcome** will be correct inhaler technique; chi-squared tests will be used to compare post VME vs. pre VME prevalence of inhaler misuse (<75% of steps correct). **Secondary outcomes** will include self-efficacy for respiratory inhalers, symptom burden (i.e., BORG and CSS), baseline self-reported utilization of health care

services for respiratory exacerbations, basic disease knowledge, and QOL. For each of these outcomes, we will evaluate whether patient characteristics (vision, age, and/or health literacy level) modifies the effectiveness of VME by evaluating interactions in the regression model. A two-tailed p-value less than 0.05 will define statistical significance.

SPECIFIC AIM 2

This will be a randomized, controlled, non-inferiority clinical trial conducted of patients admitted to the University of Chicago. Adults admitted with a history of Asthma or COPD will be eligible to participate in this study. Upon receiving consent, participants will be randomized to either the VME or TTG. Inhaler technique will be measured prior to hospital discharge before and after education (VME or TTG). This will provide data on the short-term effectiveness of the interventions. Retention will be examined at 30 days after hospital discharge.

ELIGIBILITY CRITERIA

The goal of patient selection is to enroll adults admitted with a history of physician-diagnosed Asthma or COPD.

Inclusion criteria

1. Age 18 years and older
2. Admission to the inpatient medical service and surgical service
3. Physician-diagnosed Asthma or COPD. We will enroll patients even if the primary reason for admission is not Asthma or COPD (e.g., patients admitted for heart failure, but with a physician diagnosis of COPD are eligible).
4. Physician plans to discharge patients home a metered dose inhaler (MDI; e.g., albuterol)

Exclusion criteria

1. Currently in an intensive care unit
2. Physician declines to provide consent
3. Patient unable to provide consent (e.g., history of cognitive impairment, unable to understand English) or declines to provide consent
4. Previous participant in this study

RECRUITMENT

We will employ the same recruitment plan as in the protocol for our Effectiveness of interventions to Teach Respiratory Inhaler Technique (E-TRaIN) study (Protocol #11-0248). In summary, admission logs (EPIC) at the University of Chicago will be examined each day to screen for potentially eligible patients. The treating physician will be contacted for verbal assent using standardized text. Among patients whose physician provides assent, medical records will be reviewed to ascertain eligibility and informed consent will be sought from eligible patients. A total of 142 patients will be recruited at the University of Chicago Medical Center over 15 months.

TREATMENT CONDITIONS

1. **Teach-to-Goal (TTG):** During inpatient admission, participants assigned to the TTG conditon will be provided with an intensive, iterative education and evaluation strategy that consists of the following steps: First, participants will undergo an initial assessment

of respiratory inhaler technique for MDI (and a Diskus® device if they are also prescribed medication using the device, i.e., fluticasone/salmeterol [Advair Diskus ®]) using standardized device-specific checklists (Form 1) that we have developed and used in the SARI study (Protocol #15729A), in TURI (#16900A), and in E-TRaIN (#11-0248). This will be followed by the educational intervention whereby participants will then be taught using verbal instructions and then by demonstration by a trained educator regarding the appropriate use of the MDI (and, if applicable, fluticasone/salmeterol (Advair Diskus®) using standardized written instructions (Forms 2a and 2b). Next the participants will be asked to demonstrate how they use their respiratory inhalers; their post-TTG technique will be graded using the same checklists as the initial assessment. The participants will receive additional rounds of instruction following this assessment if needed (e.g., if the participant did not achieve mastery of the MDI or Diskus® after one round of teaching). Although initial pilot work showed that two rounds was sufficient, more recent data indicates that some participants require > 2 rounds; since the essence of this education technique is to teach-to-goal, and this requires only a few additional minutes (maxium) we will attempt to teach-to-goal. The patient's hospital physician (attending physician or their designee) will be provided information about the patients inhaler technique for both inhalers after education; this will provide an opportunity for the patient to receive addition instructions by the clinical team if needed. All instructions will be provided by a dedicated trained educator; all assessments will be performed by a second research assisstant (trained assessor). The trained assessor will be masked to treatment assignment (see below).[15]

2. **Video Module Education (VME):** The RE will provide participants with a tablet device and will demonstrate how to access VME and complete the pre/post e-learning assessments. The RE will provide technical support but will neither participate directly in the education nor help with the self-assessments. The participants will first complete the pre-assessment e-learning tool on the tablet. They will then watch the video instruction that will provide a complete demonstration with verbal instructions on correct inhaler technique. Next the participants will complete the post-assessment e-learning tool. Based on participants' performance, they will be directed to further tailored video-instruction. The cycle of self-assessment and video instruction will continue until sufficient mastery has been achieved.

Other considerations:

1. *Masking:* To minimize risk of biased measurements of inhaler technique, the trained assessor will be masked to the treatment condition. Specifically, the trained assessor will not be informed about the treatment assignment, will remain outside the patients' room during the educational intervention (and the room door will remain closed during the intervention), and the trained educator will be instructed not to inform the assessor about the treatment condition. We have successfully used similar procedures for masking the trained assessor in other IRB-approved studies (see E-TRaIN, IRB # 11-0248).

2. *Patients being discharged home on a MDI and a Diskus will receive VME or TTG interventions for both devices.* If randomized to the TTG intervention, then TTG will be used to teach MDI and Diskus use; likewise if randomized to the VME intervention, then VME will be used to teach MDI and Diskus use. Based on results of screening data from the TURI study (IRB #16900A), we expect that about 10-20% of participants (10 to 20 participants enrolled in this study) will be prescribed both devices. We would like to include this option to teach two different devices (in patients prescribed both devices) to collect preliminary data

about the feasibility of VME for MDI vs. TTG for Diskus; these data would be valuable as we prepare for future studies.

PROTOCOL FOR BOTH GROUPS:

Following the screening procedures above, we will use the following steps:

10. **Obtain assent from treating physician prior to approaching patients hospitalized with asthma or COPD.** The treating physician will be contacted by a research assistant for verbal assent using standardized text (Form 3).

11. **Obtain written informed consent from patient using standardized text.** One hundred eighteen participants (N=59 TTG condition; N=59 VME condition) will be enrolled in this study.

12. **Optimize room conditions**
    - Room well lit (turn on overhead lights if necessary)
    - Patient preferably sitting up
    - Private setting (close door to patient room)
    - Interviewer should directly face patient
    - Patient should use reading glasses if they use one

13. **Assess patient's respiratory symptoms/morbidity (Forms 4-5-6), which includes the** Borg symptom score, Asthma Symptom Severity Index (ASSI), Chronic Bronchitis Symptom Questionnaire, COPD Severity Score (CSS), Airway Questionnaire (AQ-20), COPD Helplessness Index (CHI) and demographic and other clinical information.

14. **Assess patients' self-efficacy regarding respiratory inhaler use (Form 7)**

15. **Assess lung function. Perform spirometry pre and post-bronchodilator (after 2 puffs albuterol MDI via spacer) using portable KoKo spirometer. (Forms 8a and 8b).** These data will provide an objective measure of disease severity at the time of the hospitalization. We have previously demonstrated that this population has poor perception of airflow obstruction and spirometry is feasible during the inpatient setting.[16]

16. **Assess patient's inhaler technique using Inhaler checklists (Form 1;** for a MDI and, if applicable, Diskus®) by the trained assessor.

17. **Assess health literacy. Check vision (Form 9). Ask brief health literacy screen questions (Form 10a) for all participants and a**dminister STOHFLA questionnaire (Form 10b) if the patient's visual acuity is at least 20/50 in one eye (Check the patient's visual acuity in each eye using the Snellen Pocket Eye Chart (Form 11), otherwise, the patient is ineligible to complete the STOHFLA HL evaluation (will complete the BHLS questions).

    a) BHLS

We want to find out what health care information is understood by patients and what is confusing to them, in order to help us understand what patients may find confusing.
1) How often do you have problems learning about your medical condition because of difficulty understanding written information?
2) How confident are you filling out medical forms by yourself?
3) How often do you have someone help you read hospital material?

b) STOHFLA
Assess health literacy. Check vision (Form 9). Administer STOHFLA questionnaire (Form 10) if the patient's visual acuity is at least 20/50 in one eye (Check the patient's visual acuity in each eye using the Snellen Pocket Eye Chart (Form 11), otherwise, the patient is ineligible to complete the HL evaluation. Explain the purpose of this survey. Use the following text.

- We want to find out what health care information is understood by patients and what is confusing to them, in order to help us write things more clearly.
- Here are some medical instructions that you or anybody might see around the hospital. These instructions are in sentences that have some of the words missing.
- Where a word is missing, a blank line is drawn, and 4 possible words that could go in the blank appear just below it. I want you to figure out which of those 4 words should go in the blank, which word makes the sentence make sense.
- When you think you know which one it is, circle the letter in front of that word, and go on to the next one.
- When you finish the page, turn the page and keep going until you finish all the pages.
- Provide the patient a pen and a clipboard and ask them to complete the questionnaire (Form 10). If the patients ask for help with completing the questionnaire, instruct them to do the best they can and that you are not allowed to assist them. Only responses completed within 7 minutes are used in calculating a health literacy score. Record the start and stop time and any questions that were answered after 7 minutes had elapsed. Do not grade the STOFHLA until after the study visit is complete.

18. **Obtain randomization assignment from the study biostatistician.** Randomization will be block-stratified by level of health literacy to ensure an equal number of subjects within each stratum assigned to TTG and VME Patients will then receive either the TTG or VME intervention by the trained educator.

19. **Collect patient contact information and provide patient $25 in cash for participating in this study as well as parking pass/bus pass for their follow-up visit.** Record patient's social security number and obtain patients signature that they received the patient incentive.

1. **Provide patients an appointment for an in-person interview 30 days after discharge (patients will be given a letter with their appointment time).** At the in-person interview, we will ask patients to complete a short interviewer-administered questionnaire about patient's respiratory symptoms/morbidity/self-reported ability to use MDI and Diskus® inhalers, quality of life (Forms 4 and 12). We will also assess lung function (spirometry) and use of inhalers (MDI and/or Diskus). Upon completing this portion the participant will receive $50 as well as parking pass/bus pass for their next follow-up visit.

OUTCOMES/MEASURES-AIM 2

The primary and secondary outcomes are listed below. Measures for each are described. Initial measurements (Vo; see Data Collection Schedule below) will be made prior to randomization; at V1, the trained educator will assess respiratory inhaler technique by asking the subject to demonstrate use of the MDI (and Diskus® if to be discharged home on fluticasone/salmeterol). Follow-up assessment, V2, will take place at 30 days (+/-7 days) post-hospital discharge.

Primary outcome
The primary outcome will be inhaler technique. We will define inhaler technique in two ways:

c) Correct Use (i.e., >75% of steps correct)
d) Mastery (i.e., perfect technique, 100% steps correct)

This outcome will be measured at V1 and V2. We will test our hypothesis that patients will have a <u>reduced proportion of respiratory events when taught effective inhaler technique with TTG compared to BI education</u> separately for the MDI and Diskus® devices after hospital discharge. We will employ the same checklists used in the E-TRaIN study (IRB Protocol #11-0248, high reliability, kappa >0.94). These checklists will be used to measure the primary outcome: ability to use inhalers (defined as (a) correct use of inhaler: >9/12 steps for MDI; >8/10 steps for Diskus®; and (b) mastery of inhaler technique, i.e., perfect technique: 12/12 steps for MDI; 10/10 steps for Diskus®).

The **primary outcome** is the proportion of participants with MDI misuse post VME vs. TTG education. This will provide data on the short-term effectiveness of the interventions. Longer-term retention (durability) will be examined 30 days post-hospital discharge using an intention-to-treat analysis. We have powered on this binary variable. [Table 1] However, because we recognize that there is more information about which steps are less critical and those that are more critical, I will also work with Robert Gibbons, a nationally recognized expert in biostatistics, to model a mixed effects ordinal regression model with random intercepts and time effects within correlation of each participant with terms for mode (VME and TTG), as well as interaction with time, to determine the predicted probability in each category for VME and TTG of how prevalence of misuse changes over time.[17] **Mediators include** health literacy[18] and vision level[19] as I have previously identified them as important mediators of baseline inhaler misuse and/or ability to learn correct technique.[8,10]

Secondary outcomes
Secondary outcomes will include:

d) <u>Change in self-efficacy for respiratory inhalers (i.e., V0-V2).</u> We will ask all patients to state how strongly they agree or disagree with the following statement "I am confident that I know how to use [insert inhaler name] correctly" for both MDI and Diskus®. The subject will be shown an example of each inhaler. Each item will be scored on a 5-point

Likert scale (strongly disagree to strongly agree); the mean score will be used to measure self-efficacy. Cronbach's alpha will be calculated to evaluate internal consistency.

e) Symptom burden (i.e., BORG, ASSI, and CBSQ) at V1 and V2. Patient reported outcomes will be measured using a modified Borg dyspnea scale (Borg), Asthma Symptom Severity Index (ASSI), Chronic Bronchitis Symptom Questionnaire (CBSQ).

f) Acute care utilization for respiratory exacerbations at V2. Data will be collected using interviewer administrated surveys. *Respiratory events* are defined as: physician, emergency room or hospital visit, or death. This composite endpoint is due to small sample size.

The comparative effectiveness of TTG versus VME with regards to these secondary outcomes will employ simple univariate and/or multivariate logistic regression, generalized estimating equations, or generalized non-linear mixed models, as appropriate. For each of these outcomes, we will evaluate whether health literacy level modifies the effectiveness of VME (Vs. TTG) by including an interaction term in the regression model. Model assumptions will be checked. Use of two inhalers will also provide the opportunity to evaluate if the comparative effectiveness of VME (vs. TTG) varies by device. A two-tailed p-value less than 0.05 will define statistical significance. The sample size calculations demonstrate that N=59 per group will provide 80% power for an intention-to-treat analysis to test for non-inferiority of VME vs. TTG.

Secondary Outcomes include quality of life (QOL),[20] patient reported outcomes,[20-21] lung function**,** utilization of health care services (outpatient visits, ED and hospital admissions, deaths), and self-efficacy of inhaler technique.[8,10] We will also evaluate the differential effects of VME vs. TTG by participant characteristics (e.g., age, health literacy). Finally we will evaluate the preliminary comparative durability of VME vs. TTG at 30 days post-hospital discharge. We will perform all tests separately for both MDI and Diskus devices.

DATA COLLECTION SCHEDULE-AIM 2

**VISIT**	**Inpatient Visit**<br>**V0-V1**		**30 day FU**<br>**V2**
Visit duration (minutes)	60 (total for V0 to V1)		60
Visit location	Inpatient		Outpatient
Visit type	In-person		In-person
Eligibility evaluation	■		
Consent	■		
Randomization	■		
Self Efficacy: Self-reported inhaler technique	■		■
Patient demonstration of inhaler technique	■	■	■
Vision Screen			
Health Literacy	■		
Spirometry before and after bronchodilator	■		■

Review of medications	■		■
Medication count/weigh			■
Questionnaire about symptom burden, respiratory exacerbations, acute care utilization	■		■
Discharge Questionnaire		■	

Vo: initial assessment V1: after instruction V2: 30 days post-discharge follow-up

This study will require 24 months:

5. Develop/pilot test interactive video (6 month)
6. Training of personnel (1 month)
7. Begin recruitment (1-3 participants/week, for 15 months)
8. Complete follow-up of last enrollee (1 month after last enrolled participant)
9. Data analyses and preparation of final report (2 months).

The research will take place on the inpatient wards of UCM.

ANALYSES-AIM 2

Analysis plan. We will use means, medians, proportions, scatterplots, and histograms to describe the data. We will test for group differences using the *t*-test, the Wilcoxon rank sum test, Pearson's chi-squared test, or Fisher's exact tests as appropriate. We will adhere to the intention-to-treat principle when testing for differences by intervention type. The **primary outcome** will be correct inhaler technique; chi-squared tests will be used to compare post VME vs. post TTG prevalence of inhaler misuse (<75% of steps correct). **Secondary outcomes** will include change in self-efficacy for respiratory inhalers, symptom burden (i.e., BORG and CSS), self-reported utilization of health care services for respiratory exacerbations, basic disease knowledge, and QOL. The comparative effectiveness of VME versus TTG with regards to these secondary outcomes will employ simple univariate and/or multivariate logistic regression, as appropriate. For each of these outcomes, we will evaluate whether patient characteristics (vision, age, and/or health literacy level) modifies the effectiveness of VME versus TTG by evaluating interactions in the regression model. A two-tailed p-value less than 0.05 will define statistical significance. The sample size calculations (see below) demonstrate that n=59 per group will provide 80% power for an intention-to-treat analysis.

Sample size. (Table 1) The main outcome is correct MDI use (>75% steps correct). In my preliminary studies above, the proportion of participants with correct MDI use after TTG was 88-100% (n=98, n=50, respectively).[8,10] These data (expected proportion of correct MDI use of 0.95), along with a noninferiority effect size of -0.10, were used to calculate the sample size.[22] We will enroll 142 participants over 15 months and will have sufficient power (0.8) to determine if VME is non-inferior to TTG, at a significance level of p<0.05. We have experience recruiting participants and have been able to recruit up to 12 participants per month; therefore, we do not foresee any problems with recruiting this number of participants in this timeline.

Table 1: Sample Size per intervention (VME/TTG)

	**Power**				
**EFFECT SIZE**	**0.60**	**0.70**	**0.80**	**0.90**	**0.95**
**-0.05**	137	179	236	326	412
**-0.10**	35	45	59	82	103
**-0.15**	16	20	27	37	46

Interim analyses: After 50% of the target sample size have completed the study (59 participants have completed the study) or after 50% of the study period (~7 months), whichever occurs first, results of an interim analysis will be provided to a Data Safety and Monitoring Board (Drs. Dana Edelson, MD; Steve White, MD; Neda Laiteerapong, MD; physicians who are not study investigators). Investigators and research assistants will remain masked to the results of the interim analyses until the end of the study. The DSMB will be asked to consider issues related to study performance (enrollment rates, study completion rates) and differences in outcomes between groups and provide recommendations to the PI and the IRB whether to continue the study or terminate enrollment.

BENEFITS AND RISKS AIMS1 AND 2

Benefits

We are not aware of any potential benefits to study subjects.

Risks

Potential risks include:

1. The subject may be uncomfortable answering some interview questions. They can refuse to answer any question.

2. The lung function test is safe and is commonly used to measure the severity of COPD but can cause some minor chest soreness or lightheadedness; study test will be done by a trained member of the research staff with the subject in a seated position. To further minimize the risk of adverse events with spirometry, we will not perform spirometry in subjects with SBP>180 or DBP>100 mm Hg.

3. Two puffs of albuterol (for post-bronchodilator spirometry) is generally well tolerated. We have not had adverse events with 2 puffs albuterol after enrolling >170 subjects in the SARI, COAT, and TURI studies (IRBs# 15729A, 14831A, 16900A). In some cases, however, 2 puffs of albuterol can cause jitteriness, anxiety, and tachyarrhythmias. To minimize this risk, we will withhold albuterol in subjects who are hospitalized with tachyarrhythmias or palpitations or report problems after using albuterol.

4. Long acting beta-2-agonists (salmeterol or formoterol). These medications are generally well-tolerated, but can have albuterol-like adverse effects; in addition, these agents have been associated with increase risk of asthma-related deaths.

5. Inhaled corticosteroids (fluticasone/budesonide). These medications are well tolerated, but can cause thrush, dysphonia, and other less common adverse effects.

6. Loss of confidentiality. To help ensure that patients' health information remains private, we will restrict access to data collected for our study to study personnel only (via use of password-protection and locked cabinets for study documents). Study ID numbers will be generated and will be used when discussing and/or reviewing data at study meetings. Also, study reports will report results in aggregate and not contain information that can be used to identify individual patients.

Data Safety Monitoring Plan-AIM 2

The PI will review monthly data reports reviewing recruitment, consent, intervention activities, adverse events, and follow-up. Although few, if any, intervention-related adverse events are anticipated in this education-related study, the study population is at risk for poor outcomes, including life-threatening exacerbations and death. The PI will comply with prompt reporting of any unanticipated events and the Data Safety and Monitoring Board (DSMB) (Dana Edelson, MD, Steve White, MD, Neda Laiteerapong, MD). The DSMB (see interim analysis above) will also review results of the interim and final analyses.

PATIENT INCENTIVES-AIM 1 AND 2

Participants will be provided $25 for completing the initial baseline interview (only interview for AIM 1, first interview for AIM 2) and $50 completing the post-hospital discharge in-person interview. Total possible monetary compensation is $25 for AIM 1 and $75 for AIM 2. For AIM 2, participants will also be provided parking passes or bus passes for their follow-up visits as we recognize that this cost burden should not fall on the participant for participating in our study. Monetary compensation will be reduced, based on the extent to which the participante completes the study. For both AIMs all tests for this study will be done free of charge.

BIBLIOGRAPHY

1. Morbidity and Mortality 2007 Chart Book on Cardiovascular, Lung, and Blood diseases. Available at: http://www.nhlbi.nih.gov/resources/docs/07-chtbk.pdf. Accessed June 06, 2012.

2. Jencks SF, Williams MV, Coleman EA. Rehospitalizations among patients in the Medicare fee-for-service program. N Engl J Med 2009;360(14):1418-1428. PMID: 19339721.

3. Hanania NA, Darken P, Horstman D, Reisner C, Lee B, Davis S, Shah T. The efficacy and safety of Fluticasone Propionate (250ug) /Salmeterol (50ug) combined in the Diskus® Inhaler for the treatment of COPD. Chest 2003;124:834-843. PMID: 12970006.

4. Celli BR, Thomas NE, Anderson JA, Ferguson GT, Jenkins CR, Jones PW, Vestbo J, Knobil K, Yates JC, Calverley PMA. Effect of pharmacotherapy on rate of decline of lung function in chronic obstructive pulmonary disease. Results from the TORCH Study. Am J Resp Crit Care Med 2008;178:332-338. PMID: 18511702.

5. Melani, AS, Bonavia M, Cilenti V, Cinti C, Lodi M, Martucci P, Serra M, Scichilone N, Sestini P, Aliani M, Neri M, on behalf of the Gruppo Educazionale Associazione Italiana Penumologi Ospedalieri (AIPO). Inhaler mishandling remains common in real life and is associated with reduced disease control. Respir Med 2011;105:930-938. PMID: 21367593.

6. Dolovich MB, Ahrens RC, Hess DR, Anderson P, Dhand R, Rau JL, Smaldone GC, Guyatt G. Device selection and outcomes of aerosol therapy: Evidence-based guidelines: American College of Chest Physicians/American College of Asthma, Allergy, and Immunology Chest. 2005;127:335-71. PMID: 21367593.

7. The Global initiative for Chronic Obstructive Lung Disease (GOLD) Guidelines: Available at: http://www.goldcopd.com/. Accessed June 6, 2012.

7.5 Guidelines for the Diagnosis and Management of Asthma (EPR-3). Available at: https://www.nhlbi.nih.gov/guidelines/asthma/. Accessed June 6, 2012.

8. Press VG, Shah LM, Lewis S, Ivy K, Charbeneau J, Mazurek A, Badlani S, Naureckas E, Krishnan JA. Misuse of respiratory inhalers in hospitalized patients with asthma or COPD. J Gen Intern Med 2012;26(6), 635-642. PMID: 21249463 PMCID: PMC3101982.

9. Cohen DJ, Clark EC, Lawson PJ, Casucci BA, Flocke SA. Identifying teachable moments for health behavior counseling in primary care. Patient Educ Couns 2011;85:e8-e15. PMID: 21183305

10. Press VG, Arora VM, Lewis SL, Charbeneau J, Starkey J, Naureckas ET, Krishnan JA. Teaching the use of respiratory inhalers to hospitalized patients with asthma or COPD: A randomized trial (TURI). J Gen Intern Med May 2012. [Epub ahead of print.] PMID: 22592354.

11. Paasche-Orlow MK, Riekert KA, Bilderback A, Chanmugam A, Hill P, Rand CS. Tailored education may reduce health literacy disparities in asthma self-management. Am J Respir Crit Care Med 2005;172(8):980-986. PMID: 16081544 PMCID: PMC2718412.

12. Virchow JC, Crompton GK, Negro RD, Pedesen S, Magnan A, Seidenberg J, Barnes PJ. Importance of inhaler devices in the management of airway disease. Respir Med 2008;102:10-19. PMID: 17923402.

13. Jack BW, Chetty VK, Anthony D, Greenwalk JL, Sanchez GM, Johnson AE, Forsythe SR, O'Donnell JK, Paasche-Orlow MK, Manasseh C, Martin S, Culpepper L. A Reengineered Hospital Discharge Program to Decrease Rehospitalization. A Randomized Trial. Ann Intern Med 2009;150(3):178:187. PMID: 19189907. PMCID: PMC2738592.

14. Gerber BS, Brodsky IG, Lawless KA, Smolin LI, Arozullah AM, Smith EV, Berbaum ML, Heckerling PS, Eiser AR. Implementation and evaluation of a low-literacy diabetes education computer multimedia application. Diabetes Care 2005;28(7):1574-1580. PMID: 15983303.

15. Press VG, Shah LM, Lewis SL, Ivy K, Charbeneau JT, Mazurek A, Krishnan JA. Teach-to-goal intervention to teach respiratory inhaler technique in patients hospitalized for asthma or COPD. Abstract, accepted for poster presentation, Society of General Internal Medicine Annual Meeting May 2009.

16. Davis SQ, Permutt Z, Permutt S, Naureckas ET, Bilderback AL, Rand CS, Stein BD, Krishnan JA. Perception of airflow obstruction in patients hospitalized for acute asthma. *Annals of Asthma, Allergy, and Immunology.* 2009*;102(6)455-61.*

17. Hedeker D, Gibbons RD. Longitudinal Data Analysis. New Jersey: John Wiley & Sons; 2006.

18. Nurss JR, Parker RM, Williams MV, Baker, DW. Short Test of Functional Health Literacy in Adults (STOFHLA). Hartford: Peppercorn Books & Press; 2001.

19. Snellen Eye Chart: A Description and Explanation. Available at: http://precision-vision.com/index.cfm/feature/24_149/snellen-eye-chart---a-description-and-explanation.cfm. Accessed June 6, 2012.

20. Kendrick KR, Baxi SC, Smith RM. Usefulness of the modified 0-10 Borg scale in assessing the degree of dyspnea in patients with COPD and asthma. J Emerg Nurs 2002;26:216-222. PMID: 10839848.

21. Eisner MD, Trupin L, Katz PP, Yelin EH, Earnest G, Balmes J, Blanc PD. Development and validation of a survey-based COPD severity score. Chest 2005;127:1890-1897. PMID: 15947299.

22. Hung HMJ, Wang SJ, O'Neill R. A regulatory perspective on choice of margin and statistical inference issue in non-inferiority trials. Biom J 2005;47(1):28-36. PMID: 16395994.

Protocol Title: Video vs. TTG Respiratory inhaler technique Assessment and InstructioN (V-TRaIN)

University of Chicago
PI: Valerie G. Press, MD, MPH

Co-Investigators: David Meltzer, MD, PhD, Vineet Arora, MD, MAPP, Ted Naureckas, MD

Research personnel: John Kim, Nicole Woodrick, Nicole Twu, Richa Adhikari, Ashley Hull

BACKGROUND

Asthma and Chronic obstructive pulmonary disease (COPD) results in over a million hospitalizations in the United States annually[1] and COPD is the third leading cause of 30-day re-hospitalizations.[2] Clinical trials have established the efficacy of treatments primarily dispensed via respiratory inhaler devices that reduce morbidity and health care utilization if they are used correctly.[3-4] Unfortunately, the effectiveness of these medications in real-world settings is limited by the fact that patients often do not use inhalers correctly.[5-6] Current guidelines recommend assessing and teaching inhaler technique at all health care encounters, including hospitalization.[77.5] My work has found that over 75% of hospitalized patients in an urban, predominantly underserved population misuse their respiratory inhalers, highlighting a missed opportunity to educate these patients with high potential to benefit.[8] Hospitalization, therefore, provides a potential 'teachable moment' to correct this misuse.[9] My preliminary data indicate that one strategy, in-person teach-to-goal (TTG), is effective in teaching hospitalized patients proper inhaler technique and is more effective than simple verbal instruction.[10]

While TTG is a promising method to improve care for patients who use inhalers, several limitations prevent widespread adoption. First, TTG relies on in-person assessment and education, as well as training and monitoring instructors to ensure fidelity, making it time-consuming and costly. Also, because a single educational session does not ensure long-term retention,[11-12] post-discharge reinforcement may be needed, which may be impractical with in-person TTG. One potential method to surmount TTG's limitations is use of interactive video module education (VME), a method that has been used for health education in other clinical contexts.[13-14] Through iterative self-assessments and video-demonstrations on a tablet computer, VME has the potential to be less costly, maintain fidelity, and be more easily extended into the post-discharge setting than in-person TTG. However, certain questions remain about VME. It is unclear whether VME will yield similar results when compared to TTG, or whether urban, underserved patients will have the ability to, and be willing to use, VME in the post-discharge setting. Therefore, before widespread implementation of VME, it is critical to rigorously develop and test VME for inhaler education in the hospital setting. Ultimately, it will also be important to understand patients' ability and willingness to use post-discharge VME for educational reinforcement to allow for this strategy to transition patients across care settings from hospital to home. Through these funded projects, I will acquire critical data I need to develop R-01 studies focused on improving self-management for patients with obstructive pulmonary disease across care transitions.

<u>We hypothesize that interactive VME will lead to non-inferior rates of ability to demonstrate correct inhaler use compared to rates with TTG among hospitalized patients with Asthma or COPD.</u>

The specific aims to test this hypothesis are:

1. **To develop and iteratively test VME to teach correct use of metered dose inhaler (MDIs) and dry-powder Diskus® devices to hospitalized patients with Asthma or COPD.**

   We partnered with Click to Play Media™ (C2P) and Smart Sparrow to develop VME modules that (self)-assess and teach respiratory inhaler technique to hospitalized participants. We have tested the VME in focus groups (IRB #13-1139). Now that the VME is developed we will iteratively test (n=30-40) the VME strategy (up to 3 rounds (90-120 total)) to obtain important patient feedback, preferences and preliminary efficacy estimates for the module.

   We hypothesize that participants' post-VME inhaler technique will be significantly better than their pre-VME inhaler technique.

2. **To determine if the proportion of hospitalized patients with Asthma or COPD who demonstrate correct inhaler technique after receiving VME is not significantly less than (non-inferior to) the proportion who demonstrate correct inhaler technique after receiving in-person TTG.**

   To accomplish this aim we will build on our prior work with developing and evaluating the in-person TTG strategy to develop a parallel VME strategy that incorporates guideline-recommended components of assessment and instruction. We will conduct a behavioral randomized clinical trial to evaluate the relative effectiveness and durability of VME versus in-person TTG education on the ability of hospitalized patients to demonstrate correct MDI and Diskus® use. Secondarily, we will assess whether there is variation in comparative effectiveness by patient characteristics including age, gender, and health literacy level, among other important factors.

We hypothesize that interactive VME will lead to non-inferior rates of ability to demonstrate correct inhaler use compared to rates with TTG among hospitalized patients with Asthma or COPD.

SPECIFIC AIM 1

This will be a pre/post study of inpatients admitted to the University of Chicago. Adults admitted with a history of physician diagnosed COPD or asthma will be eligible to participate in this study. Upon receiving consent, participants will enrolled. Inhaler technique will be measured pre and post VME. This will provide data on the short-term efficacy of the VME.

Inclusion criteria:

1. Age 18 years and older
2. Admission to the inpatient medical service and surgical service
3. Physician-diagnosed COPD or asthma. We will enroll patients even if the primary reason for admission is not COPD or asthma (e.g., patients admitted for heart failure, but with a physician diagnosis of COPD are eligible).
4. Physician plans to discharge patients home a metered dose inhaler (MDI; e.g., albuterol)

Exclusion criteria

1. Currently in an intensive care unit
2. Physician declines to provide consent
3. Patient unable to provide consent (e.g., history of cognitive impairment, unable to understand English) or declines to provide consent

RECRUITMENT

Admission logs (EPIC) at the University of Chicago will be examined each day to screen for potentially eligible patients. The treating physician will be contacted for verbal assent using standardized text. Among patients whose physician provides assent, medical records will be reviewed to ascertain eligibility and informed consent will be sought from eligible patients. Sixty to ninety participants (approximately 30 per iterative round of VME up to 3 rounds) will be recruited at the University of Chicago Medicine over 2-6 months.

Video Module Education (VME):

The RE will provide participants with a tablet device and will demonstrate how to access VME and complete the pre/post e-learning assessments. The RE will provide technical support but will neither participate directly in the education nor help with the self-assessments. The participants will first complete the pre-assessment e-learning tool on the tablet. They will then watch the video instruction that will provide a complete demonstration with verbal instructions on correct inhaler technique. Next the participants will complete the post-assessment e-learning tool. Based on participants' performance, they will be directed to further tailored video-instruction. The cycle of self-assessment and video instruction will continue until sufficient mastery has been achieved.

Following the screening procedures above, we will use the following steps:

1. **Obtain assent from treating physician prior to approaching patients hospitalized with asthma or COPD.** The treating physician will be contacted by a research assistant for verbal assent using standardized text (Form 3).

2. **Obtain written informed consent from patient using standardized text.**

3. **Optimize room conditions**
   - Room well lit (turn on overhead lights if necessary)
   - Patient preferably sitting up
   - Private setting (close door to patient room)
   - Interviewer should directly face patient
   - Patient should use reading glasses if they use one

4. **Assess patient's respiratory symptoms/morbidity (<u>Forms 4-5-6</u>), which includes the** Borg symptom score, Asthma Symptom Severity Index (ASSI), Chronic Bronchitis Symptom Questionnaire, COPD Severity Score (CSS), Airway Questionnaire (AQ-20), COPD Helplessness Index (CHI) and demographic and other clinical information.

5. **Assess patients' self-efficacy regarding respiratory inhaler use (Form 7)**

6. **Assess lung function. Perform spirometry pre and post-bronchodilator (after 2 puffs albuterol MDI via spacer) using portable KoKo spirometer. (Forms 8a and 8b).** These data will provide an objective measure of disease severity at the time of the hospitalization. We have previously demonstrated that this population has poor perception of airflow obstruction and spirometry is feasible during the inpatient setting.[16]

7. **Assess patient's inhaler technique using Inhaler checklists (Form 1;** for a MDI) by the trained assessor.

8. **Assess health literacy. Check vision (Form 9). Ask brief health literacy screen questions (Form 10a) for all participants and a**dminister STOHFLA questionnaire (Form 10b) if the patient's visual acuity is at least 20/50 in one eye (Check the patient's visual acuity in each eye using the Snellen Pocket Eye Chart (Form 11). If the patient's visual acuity is not 20/50 in at least one eye, administer readers.

   - 4 strengths of glasses: 2.00, 2.25, 2.75, and 3.25
   - Start with 2.00, and repeat the vision screen, with each eye separately
   - If visual acuity of 20/50 is reached in at least one eye, the patient is eligible to complete the STOHFLA HL evaluation using the readers
   - If vision is not corrected after trying all of the strengths of the readers, the patient is ineligible to complete the STOHFLA HL evaluation

a) BHLS

We want to find out what health care information is understood by patients and what is confusing to them, in order to help us understand what patients may find confusing.]

1) How often do you have problems learning about your medical condition because of difficulty understanding written information?
2) How confident are you filling out medical forms by yourself?
3) How often do you have someone help you read hospital material?

b) STOHFLA

Explain the purpose of this survey. Use the following text.

- We want to find out what health care information is understood by patients and what is confusing to them, in order to help us write things more clearly.

- Here are some medical instructions that you or anybody might see around the hospital. These instructions are in sentences that have some of the words missing.

- Where a word is missing, a blank line is drawn, and 4 possible words that could go in the blank appear just below it. I want you to figure out which of those 4 words should go in the blank, which word makes the sentence make sense.

- When you think you know which one it is, circle the letter in front of that word, and go on to the next one.

- When you finish the page, turn the page and keep going until you finish all the pages.
- Provide the patient a pen and a clipboard and ask them to complete the questionnaire (Form 10). If the patients ask for help with completing the questionnaire, instruct them to do the best they can and that you are not allowed to assist them. Only responses completed within 7 minutes are used in calculating a health literacy score. Record the start and stop time and any questions that were answered after 7 minutes had elapsed. Do not grade the STOFHLA until after the study visit is complete.

9. Administer VME Survey

Explain the purpose of this survey. Use the following text.

We are thinking of doing a study that would use interactive video modules to teach patients about skills to manage their health problems. Video modules include answering a few questions, followed by watching a video to teach about a health problem or skill, followed by a few questions to see if the video was helpful.

- D1.a. How likely would you be to use an **interactive** video-module education **program** after discharge to learn about skills to help your health problem?
- D1.b. How much do you agree with this statement: “I would be willing to use the video-module education but do not have access (do not have devices and/or internet) needed to use this education” (circle one)
- D1.c. How much to you agree with this statement: “I would be willing to use the video-module education but I do not think I would know how (be able to) even though I have a device (laptop/smartphone/tablet).
- D1.d1. How much do you agree with this statement: “ I have NO interest in using the video-module education after being discharged from the hospital.”
    i. d2. If not, please explain:
- D1.e. If you think you might use the video-module education, what device would you use to participate? (check all that apply)
- D1.f. How much do you agree with this statement: “I would be willing to use video-module education after discharge home if I was provided with **access** to (a computer, tablet, cell phone with internet (smartphone), etc))”
- D1.g. g. How much to you agree with this statement: “I would be willing to use a public resource such as a library, drugstore, grocery store, church or temple to be able to use the video-module education after being discharged from the hospital.”

10. **Collect patient contact information and provide patient $25 in cash for participating in this study.** Record patient's social security number and obtain patients signature that they received the patient incentive.

OUTCOMES/MEASURES-AIM 1

The primary and secondary outcomes are listed below. Measures for each are described. Initial measurements (Vo; see Data Collection Schedule below) will be made prior to randomization; at V1, the trained educator will assess respiratory inhaler technique by asking the subject to demonstrate use of the MDI.

Primary outcome

The primary outcome will be inhaler technique. We will define inhaler technique in two ways:

a) Correct Use (i.e., >75% of steps correct)
b) Mastery (i.e., perfect technique, 100% steps correct)

This outcome will be measured at V1. We will test our hypothesis that patients will have improved inhaler technique post-VME compared to pre-VME. We will employ the same checklists used in the E-TRaIN study (IRB Protocol #11-0248, high reliability, kappa >0.94). These checklists will be used to measure the primary outcome: ability to use inhalers (defined as (a) correct use of inhaler: >9/12 steps for MDI; and (b) mastery of inhaler technique, i.e., perfect technique: 12/12 steps for MDI).

The **primary outcome** is the proportion of participants with MDI misuse post VME vs. pre VME. This will provide data on the short-term efficacy of VME.

Secondary outcomes

Secondary outcomes will include:

a) <u>Symptom burden (i.e., BORG, ASSI, and CBSQ) at V1 and V2</u>. Patient reported outcomes will be measured using a modified Borg dyspnea scale (Borg), Asthma Symptom Severity Index (ASSI), Chronic Bronchitis Symptom Questionnaire (CBSQ).
b) quality of life (QOL),[20]
c) and self-efficacy of inhaler technique.[8,10]

The efficacy of VME with regards to these secondary outcomes will employ simple univariate and/or multivariate logistic regression, generalized estimating equations, or generalized non-linear mixed models, as appropriate. For each of these outcomes, we will evaluate whether health literacy level modifies the effectiveness of VME by including an interaction term in the regression model. Model assumptions will be checked. A two-tailed p-value less than 0.05 will define statistical significance.

DATA COLLECTION SCHEDULE-AIM 1

	Inpatient Visit	
**VISIT**	**V0-V1**	
Visit duration (minutes)	60 (total for V0 to V1)	
Visit location	Inpatient	
Visit type	In-person	
Eligibility evaluation	■	
Consent	■	
Self Efficacy: Self-reported inhaler technique	■	
Patient demonstration of inhaler technique	■	■
Vision Screen		
Health Literacy	■	
VME Survey	■	
Spirometry before and after bronchodilator	■	
Review of medications	■	
Medication count/weigh		
Questionnaire about symptom burden, respiratory exacerbations, acute care utilization	■	
Discharge Questionnaire		■

Vo: initial assessment V1: after instruction V2: 30 days post-discharge follow-up

This study will require 2-6 months:

1. Pilot test interactive video round 1 (2 months)
2. Pilot test interactive video round 2 (2 months) if changes to VME needed
3. Pilot test interactive video round 3 (2 months) if changes to VME needed
4. Data analyses and preparation of final report (2 months).

The research will take place on the inpatient wards of UCM.

ANALYSES-AIM 1

Analysis plan. We will use means, medians, proportions, scatterplots, and histograms to describe the data. We will test for group differences using the *t*-test, the Wilcoxon rank sum test, Pearson's chi-squared test, or Fisher's exact tests as appropriate. We

will adhere to the intention-to-treat principle when testing for differences by intervention type. The **primary outcome** will be correct inhaler technique; chi-squared tests will be used to compare post VME vs. pre VME prevalence of inhaler misuse (<75% of steps correct). **Secondary outcomes** will include self-efficacy for respiratory inhalers, symptom burden (i.e., BORG and CSS), baseline self-reported utilization of health care services for respiratory exacerbations, basic disease knowledge, and QOL. For each of these outcomes, we will evaluate whether patient characteristics (vision, age, and/or health literacy level) modifies the effectiveness of VME by evaluating interactions in the regression model. A two-tailed p-value less than 0.05 will define statistical significance.

SPECIFIC AIM 2

This will be a randomized, controlled, non-inferiority clinical trial conducted of patients admitted to the University of Chicago. Adults admitted with a history of Asthma or COPD will be eligible to participate in this study. Upon receiving consent, participants will be randomized to either the VME or TTG. Inhaler technique will be measured prior to hospital discharge before and after education (VME or TTG). This will provide data on the short-term effectiveness of the interventions. Retention will be examined at 30 days after hospital discharge.

ELIGIBILITY CRITERIA

The goal of patient selection is to enroll adults admitted with a history of physician-diagnosed Asthma or COPD.

Inclusion criteria

1. Age 18 years and older
2. Admission to the inpatient medical service and surgical service
3. Physician-diagnosed Asthma or COPD. We will enroll patients even if the primary reason for admission is not Asthma or COPD (e.g., patients admitted for heart failure, but with a physician diagnosis of COPD are eligible).
4. Physician plans to discharge patients home a metered dose inhaler (MDI; e.g., albuterol)

Exclusion criteria

1. Currently in an intensive care unit
2. Physician declines to provide consent
3. Patient unable to provide consent (e.g., history of cognitive impairment, unable to understand English) or declines to provide consent
4. Previous participant in this study

RECRUITMENT

We will employ the same recruitment plan as in the protocol for our Effectiveness of interventions to Teach Respiratory Inhaler Technique (E-TRaIN) study (Protocol #11-0248). In summary, admission logs (EPIC) at the University of Chicago will be examined each day to screen for potentially eligible patients. The treating physician will be contacted for verbal assent using standardized text. Among patients whose physician provides assent, medical records will be reviewed to ascertain eligibility and informed consent will be sought from eligible patients. A total of 142 patients will be recruited at the University of Chicago Medical Center over 15 months.

TREATMENT CONDITIONS

1. **Teach-to-Goal (TTG):** During inpatient admission, participants assigned to the TTG conditon will be provided with an intensive, iterative education and evaluation strategy that consists of the following steps: First, participants will undergo an initial assessment of respiratory inhaler technique for MDI (and a Diskus® device if they are also prescribed medication using the device, i.e., fluticasone/salmeterol [Advair Diskus ®]) using standardized device-specific checklists (Form 1) that we have developed and used in the SARI study (Protocol #15729A), in TURI (#16900A), and in E-TRaIN (#11-0248). This will be followed by the educational intervention whereby participants will then be taught using verbal instructions and then by demonstration by a trained educator regarding the appropriate use of the MDI (and, if applicable, fluticasone/salmeterol (Advair Diskus®) using standardized written instructions (Forms 2a and 2b). Next the participants will be asked to demonstrate how they use their respiratory inhalers; their post-TTG technique will be graded using the same checklists as the initial assessment. The participants will receive additional rounds of instruction following this assessment if needed (e.g., if the participant did not achieve mastery of the MDI or Diskus® after one round of teaching). Although initial pilot work showed that two rounds was sufficient, more recent data indicates that some participants require > 2 rounds; since the essence of this education technique is to teach-to-goal, and this requires only a few additional minutes (maxium) we will attempt to teach-to-goal. The patient's hospital physician (attending physician or their designee) will be provided information about the patients inhaler technique for both inhalers after education; this will provide an opportunity for the patient to receive addition instructions by the clinical team if needed. All instructions will be provided by a dedicated trained educator; all assessments will be performed by a second research assisstant (trained assessor). The trained assessor will be masked to treatment assignment (see below).[15]
2. **Video Module Education (VME):** The RE will provide participants with a tablet device and will demonstrate how to access VME and complete the pre/post e-learning assessments. The RE will provide technical support but will neither participate directly in the education nor help with the self-assessments. The participants will first complete the pre-assessment e-learning tool on the tablet. They will then watch the video instruction that will provide a complete demonstration with verbal instructions on correct inhaler technique. Next the participants will complete the post-assessment e-learning tool. Based on participants' performance, they will be directed to further tailored video-instruction. The cycle of self-assessment and video instruction will continue until sufficient mastery has been achieved.

<u>Other considerations:</u>

1. *Masking:* To minimize risk of biased measurements of inhaler technique, the trained assessor will be masked to the treatment condition. Specifically, the trained assessor will not be informed about the treatment assignment, will remain outside the patients' room during the educational intervention (and the room door will remain closed during the intervention), and the trained educator will be instructed not to inform the assessor about the treatment condition. We have successfully used similar procedures for masking the trained assessor in other IRB-approved studies (see E-TRaIN, IRB # 11-0248).

2. *Patients being discharged home on a MDI and a Diskus will receive VME or TTG interventions for both devices.* If randomized to the TTG intervention, then TTG will be used to teach MDI and Diskus use; likewise if randomized to the VME intervention, then VME will

be used to teach MDI and Diskus use. Based on results of screening data from the TURI study (IRB #16900A), we expect that about 10-20% of participants (10 to 20 participants enrolled in this study) will be prescribed both devices. We would like to include this option to teach two different devices (in patients prescribed both devices) to collect preliminary data about the feasibility of VME for MDI vs. TTG for Diskus; these data would be valuable as we prepare for future studies.

PROTOCOL FOR BOTH GROUPS:

Following the screening procedures above, we will use the following steps:

11. **Obtain assent from treating physician prior to approaching patients hospitalized with asthma or COPD.** The treating physician will be contacted by a research assistant for verbal assent using standardized text (Form 3).

12. **Obtain written informed consent from patient using standardized text.** One hundred eighteen participants (N=59 TTG condition; N=59 VME condition) will be enrolled in this study.

13. **Optimize room conditions**
    - Room well lit (turn on overhead lights if necessary)
    - Patient preferably sitting up
    - Private setting (close door to patient room)
    - Interviewer should directly face patient
    - Patient should use reading glasses if they use one

14. **Assess patient's respiratory symptoms/morbidity (Forms 4-5-6), which includes the** Borg symptom score, Asthma Symptom Severity Index (ASSI), Chronic Bronchitis Symptom Questionnaire, COPD Severity Score (CSS), Airway Questionnaire (AQ-20), COPD Helplessness Index (CHI) and demographic and other clinical information.

15. **Assess patients' self-efficacy regarding respiratory inhaler use (Form 7)**

16. **Assess lung function. Perform spirometry pre and post-bronchodilator (after 2 puffs albuterol MDI via spacer) using portable KoKo spirometer. (Forms 8a and 8b).** These data will provide an objective measure of disease severity at the time of the hospitalization. We have previously demonstrated that this population has poor perception of airflow obstruction and spirometry is feasible during the inpatient setting.[16]

17. **Assess patient's inhaler technique using Inhaler checklists (Form 1;** for a MDI and, if applicable, Diskus®) by the trained assessor.

18. **Assess health literacy. Check vision (Form 9). Ask brief health literacy screen questions (Form 10a) for all participants and a**dminister STOHFLA questionnaire (Form 10b) if the patient's visual acuity is at least 20/50 in one eye (Check the patient's visual acuity in each eye using the Snellen Pocket Eye Chart (Form 11), otherwise, the patient is ineligible to complete the STOHFLA HL evaluation (will complete the BHLS questions).

a) BHLS

We want to find out what health care information is understood by patients and what is confusing to them, in order to help us understand what patients may find confusing.

1) How often do you have problems learning about your medical condition because of difficulty understanding written information?
2) How confident are you filling out medical forms by yourself?
3) How often do you have someone help you read hospital material?

b) STOHFLA

Assess health literacy. Check vision (Form 9). Administer STOHFLA questionnaire (Form 10) if the patient's visual acuity is at least 20/50 in one eye (Check the patient's visual acuity in each eye using the Snellen Pocket Eye Chart (Form 11), otherwise, the patient is ineligible to complete the HL evaluation. Explain the purpose of this survey. Use the following text.

- We want to find out what health care information is understood by patients and what is confusing to them, in order to help us write things more clearly.
- Here are some medical instructions that you or anybody might see around the hospital. These instructions are in sentences that have some of the words missing.
- Where a word is missing, a blank line is drawn, and 4 possible words that could go in the blank appear just below it. I want you to figure out which of those 4 words should go in the blank, which word makes the sentence make sense.
- When you think you know which one it is, circle the letter in front of that word, and go on to the next one.
- When you finish the page, turn the page and keep going until you finish all the pages.
- Provide the patient a pen and a clipboard and ask them to complete the questionnaire (Form 10). If the patients ask for help with completing the questionnaire, instruct them to do the best they can and that you are not allowed to assist them. Only responses completed within 7 minutes are used in calculating a health literacy score. Record the start and stop time and any questions that were answered after 7 minutes had elapsed. Do not grade the STOFHLA until after the study visit is complete.

19. **Obtain randomization assignment from the study biostatistician.** Randomization will be block-stratified by level of health literacy to ensure an equal number of subjects within each stratum assigned to TTG and VME Patients will then receive either the TTG or VME intervention by the trained educator.

20. **Collect patient contact information and provide patient $25 in cash for participating in this study as well as parking pass/bus pass for their follow-up visit.** Record patient's social security number and obtain patients signature that they received the patient incentive.

1. **Provide patients an appointment for an in-person interview 30 days after discharge (patients will be given a letter with their appointment time).** At the in-person interview, we will ask patients to complete a short interviewer-administered questionnaire about patient's respiratory symptoms/morbidity/self-reported ability to use MDI and Diskus® inhalers, quality of life (Forms 4 and 12). We will also assess lung function (spirometry) and use of inhalers (MDI and/or Diskus). Upon completing this portion the participant will receive $50 as well as parking pass/bus pass for their next follow-up visit.

OUTCOMES/MEASURES-AIM 2

The primary and secondary outcomes are listed below. Measures for each are described. Initial measurements (Vo; see Data Collection Schedule below) will be made prior to randomization; at V1, the trained educator will assess respiratory inhaler technique by asking the subject to demonstrate use of the MDI (and Diskus® if to be discharged home on fluticasone/salmeterol). Follow-up assessment, V2, will take place at 30 days (+/-7 days) post-hospital discharge.

Primary outcome
The primary outcome will be inhaler technique. We will define inhaler technique in two ways:

c) Correct Use (i.e., >75% of steps correct)
d) Mastery (i.e., perfect technique, 100% steps correct)

This outcome will be measured at V1 and V2. We will test our hypothesis that patients will have a reduced proportion of respiratory events when taught effective inhaler technique with TTG compared to BI education separately for the MDI and Diskus® devices after hospital discharge. We will employ the same checklists used in the E-TRaIN study (IRB Protocol #11-0248, high reliability, kappa >0.94). These checklists will be used to measure the primary outcome: ability to use inhalers (defined as (a) correct use of inhaler: >9/12 steps for MDI; >8/10 steps for Diskus®; and (b) mastery of inhaler technique, i.e., perfect technique: 12/12 steps for MDI; 10/10 steps for Diskus®).

The **primary outcome** is the proportion of participants with MDI misuse post VME vs. TTG education. This will provide data on the short-term effectiveness of the interventions. Longer-term retention (durability) will be examined 30 days post-hospital discharge using an intention-to-treat analysis. We have powered on this binary variable. [Table 1] However, because we recognize that there is more information about which steps are less critical and those that are more critical, I will also work with Robert Gibbons, a nationally recognized expert in biostatistics, to model a mixed effects ordinal regression model with random intercepts and time effects within correlation of each participant with terms for mode (VME and TTG), as well as interaction with time, to determine the predicted probability in each category for VME and TTG of how prevalence of misuse changes over time.[17] **Mediators include** health literacy[18] and vision level[19] as I have previously identified them as important mediators of baseline inhaler misuse and/or ability to learn correct technique.[8,10]

Secondary outcomes
Secondary outcomes will include:

d) Change in self-efficacy for respiratory inhalers (i.e., V0-V2). We will ask all patients to state how strongly they agree or disagree with the following statement "I am confident that I know how to use [insert inhaler name] correctly" for both MDI and Diskus®. The

subject will be shown an example of each inhaler. Each item will be scored on a 5-point Likert scale (strongly disagree to strongly agree); the mean score will be used to measure self-efficacy. Cronbach's alpha will be calculated to evaluate internal consistency.

e) <u>Symptom burden (i.e., BORG, ASSI, and CBSQ) at V1 and V2</u>. Patient reported outcomes will be measured using a modified Borg dyspnea scale (Borg), Asthma Symptom Severity Index (ASSI), Chronic Bronchitis Symptom Questionnaire (CBSQ).

f) <u>Acute care utilization for respiratory exacerbations at V2</u>. Data will be collected using interviewer administrated surveys. *Respiratory events* are defined as: physician, emergency room or hospital visit, or death. This composite endpoint is due to small sample size.

The comparative effectiveness of TTG versus VME with regards to these secondary outcomes will employ simple univariate and/or multivariate logistic regression, generalized estimating equations, or generalized non-linear mixed models, as appropriate. For each of these outcomes, we will evaluate whether health literacy level modifies the effectiveness of VME (Vs. TTG) by including an interaction term in the regression model. Model assumptions will be checked. Use of two inhalers will also provide the opportunity to evaluate if the comparative effectiveness of VME (vs. TTG) varies by device. A two-tailed p-value less than 0.05 will define statistical significance. The sample size calculations demonstrate that N=59 per group will provide 80% power for an intention-to-treat analysis to test for non-inferiority of VME vs. TTG.

Secondary Outcomes include quality of life (QOL),[20] patient reported outcomes,[20-21] lung function, utilization of health care services (outpatient visits, ED and hospital admissions, deaths), and self-efficacy of inhaler technique.[8,10] We will also evaluate the differential effects of VME vs. TTG by participant characteristics (e.g., age, health literacy). Finally we will evaluate the preliminary comparative durability of VME vs. TTG at 30 days post-hospital discharge. We will perform all tests separately for both MDI and Diskus devices.

DATA COLLECTION SCHEDULE-AIM 2

**VISIT**	**Inpatient Visit**<br>**V0-V1**		**30 day FU**<br>**V2**
Visit duration (minutes)	60 (total for V0 to V1)		60
Visit location	Inpatient		Outpatient
Visit type	In-person		In-person
Eligibility evaluation	■		
Consent	■		
Randomization	■		
Self Efficacy: Self-reported inhaler technique	■		■
Patient demonstration of inhaler technique	■	■	■
Vision Screen			
Health Literacy	■		
Spirometry before and after	■		■

bronchodilator			
Review of medications	■		■
Medication count/weigh			■
Questionnaire about symptom burden, respiratory exacerbations, acute care utilization	■		■
Discharge Questionnaire		■	

Vo: initial assessment V1: after instruction V2: 30 days post-discharge follow-up

This study will require 24 months:

5. Develop/pilot test interactive video (6 month)
6. Training of personnel (1 month)
7. Begin recruitment (1-3 participants/week, for 15 months)
8. Complete follow-up of last enrollee (1 month after last enrolled participant)
9. Data analyses and preparation of final report (2 months).

The research will take place on the inpatient wards of UCM.

ANALYSES-AIM 2

Analysis plan. We will use means, medians, proportions, scatterplots, and histograms to describe the data. We will test for group differences using the *t*-test, the Wilcoxon rank sum test, Pearson's chi-squared test, or Fisher's exact tests as appropriate. We will adhere to the intention-to-treat principle when testing for differences by intervention type. The **primary outcome** will be correct inhaler technique; chi-squared tests will be used to compare post VME vs. post TTG prevalence of inhaler misuse (<75% of steps correct). **Secondary outcomes** will include change in self-efficacy for respiratory inhalers, symptom burden (i.e., BORG and CSS), self-reported utilization of health care services for respiratory exacerbations, basic disease knowledge, and QOL. The comparative effectiveness of VME versus TTG with regards to these secondary outcomes will employ simple univariate and/or multivariate logistic regression, as appropriate. For each of these outcomes, we will evaluate whether patient characteristics (vision, age, and/or health literacy level) modifies the effectiveness of VME versus TTG by evaluating interactions in the regression model. A two-tailed p-value less than 0.05 will define statistical significance. The sample size calculations (see below) demonstrate that n=59 per group will provide 80% power for an intention-to-treat analysis.

Sample size. (Table 1) The main outcome is correct MDI use (>75% steps correct). In my preliminary studies above, the proportion of participants with correct MDI use after TTG was 88-100% (n=98, n=50, respectively).[8,10] These data (expected proportion of correct MDI use of 0.95), along with a noninferiority effect size of -0.10, were used to calculate the sample size.[22] We will enroll 142 participants over 15 months and will have sufficient power (0.8) to determine if VME is non-inferior to TTG, at a significance level of p<0.05. We have experience recruiting participants and have been able to recruit up to 12 participants per month; therefore, we do not foresee any problems with recruiting this number of participants in this timeline.

**Table 1: Sample Size per intervention (VME/TTG)**					
	**Power**				
**EFFECT SIZE**	**0.60**	**0.70**	**0.80**	**0.90**	**0.95**
**-0.05**	137	179	236	326	412
**-0.10**	35	45	59	82	103
**-0.15**	16	20	27	37	46

Interim analyses: After 50% of the target sample size have completed the study (59 participants have completed the study) or after 50% of the study period (~7 months), whichever occurs first, results of an interim analysis will be provided to a Data Safety and Monitoring Board (Drs. Dana Edelson, MD; Steve White, MD; Neda Laiteerapong, MD; physicians who are not study investigators). Investigators and research assistants will remain masked to the results of the interim analyses until the end of the study. The DSMB will be asked to consider issues related to study performance (enrollment rates, study completion rates) and differences in outcomes between groups and provide recommendations to the PI and the IRB whether to continue the study or terminate enrollment.

BENEFITS AND RISKS AIMS1 AND 2

Benefits

We are not aware of any potential benefits to study subjects.

Risks

Potential risks include:

1. The subject may be uncomfortable answering some interview questions. They can refuse to answer any question.

2. The lung function test is safe and is commonly used to measure the severity of COPD but can cause some minor chest soreness or lightheadedness; study test will be done by a trained member of the research staff with the subject in a seated position. To further minimize the risk of adverse events with spirometry, we will not perform spirometry in subjects with SBP>180 or DBP>100 mm Hg.

3. Two puffs of albuterol (for post-bronchodilator spirometry) is generally well tolerated. We have not had adverse events with 2 puffs albuterol after enrolling >170 subjects in the SARI, COAT, and TURI studies (IRBs# 15729A, 14831A, 16900A). In some cases, however, 2 puffs of albuterol can cause jitteriness, anxiety, and tachyarrhythmias. To minimize this risk, we will withhold albuterol in subjects who are hospitalized with tachyarrhythmias or palpitations or report problems after using albuterol.

4. Long acting beta-2-agonists (salmeterol or formoterol). These medications are generally well-tolerated, but can have albuterol-like adverse effects; in addition, these agents have been associated with increase risk of asthma-related deaths.

5. Inhaled corticosteroids (fluticasone/budesonide). These medications are well tolerated, but can cause thrush, dysphonia, and other less common adverse effects.

6. Loss of confidentiality. To help ensure that patients' health information remains private, we will restrict access to data collected for our study to study personnel only (via use of password-protection and locked cabinets for study documents). Study ID numbers will be generated and will be used when discussing and/or reviewing data at study meetings. Also, study reports will report results in aggregate and not contain information that can be used to identify individual patients.

Data Safety Monitoring Plan-AIM 2

The PI will review monthly data reports reviewing recruitment, consent, intervention activities, adverse events, and follow-up. Although few, if any, intervention-related adverse events are anticipated in this education-related study, the study population is at risk for poor outcomes, including life-threatening exacerbations and death. The PI will comply with prompt reporting of any unanticipated events and the Data Safety and Monitoring Board (DSMB) (Dana Edelson, MD, Steve White, MD, Neda Laiteerapong, MD). The DSMB (see interim analysis above) will also review results of the interim and final analyses.

PATIENT INCENTIVES-AIM 1 AND 2

Participants will be provided $25 for completing the initial baseline interview (only interview for AIM 1, first interview for AIM 2) and $50 completing the post-hospital discharge in-person interview. Total possible monetary compensation is $25 for AIM 1 and $75 for AIM 2. For AIM 2, participants will also be provided parking passes or bus passes for their follow-up visits as we recognize that this cost burden should not fall on the participant for participating in our study. Monetary compensation will be reduced, based on the extent to which the participante completes the study. For both AIMs all tests for this study will be done free of charge.

BIBLIOGRAPHY

1. Morbidity and Mortality 2007 Chart Book on Cardiovascular, Lung, and Blood diseases. Available at: http://www.nhlbi.nih.gov/resources/docs/07-chtbk.pdf. Accessed June 06, 2012.

2. Jencks SF, Williams MV, Coleman EA. Rehospitalizations among patients in the Medicare fee-for-service program. N Engl J Med 2009;360(14):1418-1428. PMID: 19339721.

3. Hanania NA, Darken P, Horstman D, Reisner C, Lee B, Davis S, Shah T. The efficacy and safety of Fluticasone Propionate (250ug) /Salmeterol (50ug) combined in the Diskus® Inhaler for the treatment of COPD. Chest 2003;124:834-843. PMID: 12970006.

4. Celli BR, Thomas NE, Anderson JA, Ferguson GT, Jenkins CR, Jones PW, Vestbo J, Knobil K, Yates JC, Calverley PMA. Effect of pharmacotherapy on rate of decline of lung function in chronic obstructive pulmonary disease. Results from the TORCH Study. Am J Resp Crit Care Med 2008;178:332-338. PMID: 18511702.

5. Melani, AS, Bonavia M, Cilenti V, Cinti C, Lodi M, Martucci P, Serra M, Scichilone N, Sestini P, Aliani M, Neri M, on behalf of the Gruppo Educazionale Associazione Italiana Penumologi Ospedalieri (AIPO). Inhaler mishandling remains common in real life and is associated with reduced disease control. Respir Med 2011;105:930-938. PMID: 21367593.

6. Dolovich MB, Ahrens RC, Hess DR, Anderson P, Dhand R, Rau JL, Smaldone GC, Guyatt G. Device selection and outcomes of aerosol therapy: Evidence-based guidelines: American College of Chest Physicians/American College of Asthma, Allergy, and Immunology Chest. 2005;127:335-71. PMID: 21367593.

7. The Global initiative for Chronic Obstructive Lung Disease (GOLD) Guidelines: Available at: http://www.goldcopd.com/. Accessed June 6, 2012.

7.5 Guidelines for the Diagnosis and Management of Asthma (EPR-3). Available at: https://www.nhlbi.nih.gov/guidelines/asthma/. Accessed June 6, 2012.

8. Press VG, Shah LM, Lewis S, Ivy K, Charbeneau J, Mazurek A, Badlani S, Naureckas E, Krishnan JA. Misuse of respiratory inhalers in hospitalized patients with asthma or COPD. J Gen Intern Med 2012;26(6), 635-642. PMID: 21249463 PMCID: PMC3101982.

9. Cohen DJ, Clark EC, Lawson PJ, Casucci BA, Flocke SA. Identifying teachable moments for health behavior counseling in primary care. Patient Educ Couns 2011;85:e8-e15. PMID: 21183305

10. Press VG, Arora VM, Lewis SL, Charbeneau J, Starkey J, Naureckas ET, Krishnan JA. Teaching the use of respiratory inhalers to hospitalized patients with asthma or COPD: A randomized trial (TURI). J Gen Intern Med May 2012. [Epub ahead of print.] PMID: 22592354.

11. Paasche-Orlow MK, Riekert KA, Bilderback A, Chanmugam A, Hill P, Rand CS. Tailored education may reduce health literacy disparities in asthma self-management. Am J Respir Crit Care Med 2005;172(8):980-986. PMID: 16081544 PMCID: PMC2718412.

12. Virchow JC, Crompton GK, Negro RD, Pedesen S, Magnan A, Seidenberg J, Barnes PJ. Importance of inhaler devices in the management of airway disease. Respir Med 2008;102:10-19. PMID: 17923402.

13. Jack BW, Chetty VK, Anthony D, Greenwalk JL, Sanchez GM, Johnson AE, Forsythe SR, O'Donnell JK, Paasche-Orlow MK, Manasseh C, Martin S, Culpepper L. A Reengineered Hospital Discharge Program to Decrease Rehospitalization. A Randomized Trial. Ann Intern Med 2009;150(3):178:187. PMID: 19189907. PMCID: PMC2738592.

14. Gerber BS, Brodsky IG, Lawless KA, Smolin LI, Arozullah AM, Smith EV, Berbaum ML, Heckerling PS, Eiser AR. Implementation and evaluation of a low-literacy diabetes education computer multimedia application. Diabetes Care 2005;28(7):1574-1580. PMID: 15983303.

15. Press VG, Shah LM, Lewis SL, Ivy K, Charbeneau JT, Mazurek A, Krishnan JA. Teach-to-goal intervention to teach respiratory inhaler technique in patients hospitalized for asthma or COPD. Abstract, accepted for poster presentation, Society of General Internal Medicine Annual Meeting May 2009.

16. Davis SQ, Permutt Z, Permutt S, Naureckas ET, Bilderback AL, Rand CS, Stein BD, Krishnan JA. Perception of airflow obstruction in patients hospitalized for acute asthma. *Annals of Asthma, Allergy, and Immunology.* 2009*;102(6)455-61.*

17. Hedeker D, Gibbons RD. Longitudinal Data Analysis. New Jersey: John Wiley & Sons; 2006.

18. Nurss JR, Parker RM, Williams MV, Baker, DW. Short Test of Functional Health Literacy in Adults (STOFHLA). Hartford: Peppercorn Books & Press; 2001.

19. Snellen Eye Chart: A Description and Explanation. Available at: http://precision-vision.com/index.cfm/feature/24_149/snellen-eye-chart---a-description-and-explanation.cfm. Accessed June 6, 2012.

20. Kendrick KR, Baxi SC, Smith RM. Usefulness of the modified 0-10 Borg scale in assessing the degree of dyspnea in patients with COPD and asthma. J Emerg Nurs 2002;26:216-222. PMID: 10839848.

21. Eisner MD, Trupin L, Katz PP, Yelin EH, Earnest G, Balmes J, Blanc PD. Development and validation of a survey-based COPD severity score. Chest 2005;127:1890-1897. PMID: 15947299.

22. Hung HMJ, Wang SJ, O'Neill R. A regulatory perspective on choice of margin and statistical inference issue in non-inferiority trials. Biom J 2005;47(1):28-36. PMID: 16395994.

Version: 19October2015

Protocol Title: Video vs. TTG Respiratory inhaler technique Assessment and InstructioN (V-TRaIN)

University of Chicago
PI: Valerie G. Press, MD, MPH

Co-Investigators: David Meltzer, MD, PhD, Vineet Arora, MD, MAPP, Ted Naureckas, MD

Research personnel: John Kim, Nicole Woodrick, Nicole Twu, Ashley Hull, Ashley Snyder

BACKGROUND

Asthma and Chronic obstructive pulmonary disease (COPD) results in over a million hospitalizations in the United States annually[1] and COPD is the third leading cause of 30-day re-hospitalizations.[2] Clinical trials have established the efficacy of treatments primarily dispensed via respiratory inhaler devices that reduce morbidity and health care utilization if they are used correctly.[3-4] Unfortunately, the effectiveness of these medications in real-world settings is limited by the fact that patients often do not use inhalers correctly.[5-6] Current guidelines recommend assessing and teaching inhaler technique at all health care encounters, including hospitalization.[77.5] My work has found that over 75% of hospitalized patients in an urban, predominantly underserved population misuse their respiratory inhalers, highlighting a missed opportunity to educate these patients with high potential to benefit.[8] Hospitalization, therefore, provides a potential ‘teachable moment’ to correct this misuse.[9] My preliminary data indicate that one strategy, in-person teach-to-goal (TTG), is effective in teaching hospitalized patients proper inhaler technique and is more effective than simple verbal instruction.[10]

While TTG is a promising method to improve care for patients who use inhalers, several limitations prevent widespread adoption. First, TTG relies on in-person assessment and education, as well as training and monitoring instructors to ensure fidelity, making it time-consuming and costly. Also, because a single educational session does not ensure long-term retention,[11-12] post-discharge reinforcement may be needed, which may be impractical with in-person TTG. One potential method to surmount TTG's limitations is use of interactive video module education (VME), a method that has been used for health education in other clinical contexts.[13-14] Through iterative self-assessments and video-demonstrations on a tablet computer, VME has the potential to be less costly, maintain fidelity, and be more easily extended into the post-discharge setting than in-person TTG. However, certain questions remain about VME. It is unclear whether VME will yield similar results when compared to TTG, or whether urban, underserved patients will have the ability to, and be willing to use, VME in the post-discharge setting. Therefore, before widespread implementation of VME, it is critical to rigorously develop and test VME for inhaler education in the hospital setting. Ultimately, it will also be important to understand patients’ ability and willingness to use post-discharge VME for educational reinforcement to allow for this strategy to transition patients across care settings from hospital to home. Through these funded projects, I will acquire critical data I need to develop R-01 studies focused on improving self-management for patients with obstructive pulmonary disease across care transitions.

<u>We hypothesize that interactive VME will lead to non-inferior rates of ability to demonstrate correct inhaler use compared to rates with TTG among hospitalized patients with Asthma or COPD.</u>

The specific aims to test this hypothesis are:

1. **To develop and iteratively test VME to teach correct use of metered dose inhaler (MDIs) and dry-powder Diskus® devices to hospitalized patients with Asthma or COPD.**

   We partnered with Click to Play Media™ (C2P) and Smart Sparrow to develop VME modules that (self)-assess and teach respiratory inhaler technique to hospitalized participants. We have tested the VME in focus groups (IRB #13-1139). Now that the VME is developed we will iteratively test (n=30-40) the VME strategy (up to 3 rounds (90-120 total)) to obtain important patient feedback, preferences and preliminary efficacy estimates for the module.

   We hypothesize that participants' post-VME inhaler technique will be significantly better than their pre-VME inhaler technique.

2. **To determine if the proportion of hospitalized patients with Asthma or COPD who demonstrate correct inhaler technique after receiving VME is not significantly less than (non-inferior to) the proportion who demonstrate correct inhaler technique after receiving in-person TTG.**

   To accomplish this aim we will build on our prior work with developing and evaluating the in-person TTG strategy to develop a parallel VME strategy that incorporates guideline-recommended components of assessment and instruction. We will conduct a behavioral randomized clinical trial to evaluate the relative effectiveness and durability of VME versus in-person TTG education on the ability of hospitalized patients to demonstrate correct MDI and Diskus® use. Secondarily, we will assess whether there is variation in comparative effectiveness by patient characteristics including age, gender, and health literacy level, among other important factors.

<u>We hypothesize that interactive VME will lead to non-inferior rates of ability to demonstrate correct inhaler use compared to rates with TTG among hospitalized patients with Asthma or COPD.</u>

<u>SPECIFIC AIM 1</u>

This will be a pre/post study of inpatients admitted to the University of Chicago. Adults admitted with a history of physician diagnosed COPD or asthma will be eligible to participate in this study. Upon receiving consent, participants will enrolled. Inhaler technique will be measured pre and post VME. This will provide data on the short-term efficacy of the VME.

Inclusion criteria:

1. Age 18 years and older
2. Admission to the inpatient medical service and surgical service
3. Physician-diagnosed COPD or asthma. We will enroll patients even if the primary reason for admission is not COPD or asthma (e.g., patients admitted for heart failure, but with a physician diagnosis of COPD are eligible).
4. Physician plans to discharge patients home a metered dose inhaler (MDI; e.g., albuterol)

Exclusion criteria

1. Currently in an intensive care unit
2. Physician declines to provide consent
3. Patient unable to provide consent (e.g., history of cognitive impairment, unable to understand English) or declines to provide consent

RECRUITMENT

Admission logs (EPIC) at the University of Chicago will be examined each day to screen for potentially eligible patients. The treating physician will be contacted for verbal assent using standardized text. Among patients whose physician provides assent, medical records will be reviewed to ascertain eligibility and informed consent will be sought from eligible patients. Sixty to ninety participants (approximately 30 per iterative round of VME up to 3 rounds) will be recruited at the University of Chicago Medicine over 2-6 months.

Video Module Education (VME):

The RE will provide participants with a tablet device and will demonstrate how to access VME and complete the pre/post e-learning assessments. The RE will provide technical support but will neither participate directly in the education nor help with the self-assessments. The participants will first complete the pre-assessment e-learning tool on the tablet. They will then watch the video instruction that will provide a complete demonstration with verbal instructions on correct inhaler technique. Next the participants will complete the post-assessment e-learning tool. Based on participants' performance, they will be directed to further tailored video-instruction. The cycle of self-assessment and video instruction will continue until sufficient mastery has been achieved.

Following the screening procedures above, we will use the following steps:

1. **Obtain assent from treating physician prior to approaching patients hospitalized with asthma or COPD.** The treating physician will be contacted by a research assistant for verbal assent using standardized text (Form 3).

2. **Obtain written informed consent from patient using standardized text.**

3. **Optimize room conditions**
   - Room well lit (turn on overhead lights if necessary)
   - Patient preferably sitting up
   - Private setting (close door to patient room)
   - Interviewer should directly face patient
   - Patient should use reading glasses if they use one

4. **Assess patient's respiratory symptoms/morbidity (<u>Forms 4-5-6</u>), which includes the** Borg symptom score, Asthma Symptom Severity Index (ASSI), Chronic Bronchitis Symptom Questionnaire, COPD Severity Score (CSS), Airway Questionnaire (AQ-20), COPD Helplessness Index (CHI) and demographic and other clinical information.

5. **Assess patients' self-efficacy regarding respiratory inhaler use (Form 7)**

6. **Assess lung function. Perform spirometry pre and post-bronchodilator (after 2 puffs albuterol MDI via spacer) using portable KoKo spirometer. (Forms 8a and 8b).** These data will provide an objective measure of disease severity at the time of the hospitalization. We have previously demonstrated that this population has poor perception of airflow obstruction and spirometry is feasible during the inpatient setting.[16]

7. **Assess patient's inhaler technique using Inhaler checklists (Form 1;** for a MDI) by the trained assessor.

8. **Assess health literacy. Check vision (Form 9). Ask brief health literacy screen questions (Form 10a) for all participants and a**dminister STOHFLA questionnaire (Form 10b) if the patient's visual acuity is at least 20/50 in one eye (Check the patient's visual acuity in each eye using the Snellen Pocket Eye Chart (Form 11). If the patient's visual acuity is not 20/50 in at least one eye, administer readers.

   - 4 strengths of glasses: 2.00, 2.25, 2.75, and 3.25
   - Start with 2.00, and repeat the vision screen, with each eye separately
   - If visual acuity of 20/50 is reached in at least one eye, the patient is eligible to complete the STOHFLA HL evaluation using the readers
   - If vision is not corrected after trying all of the strengths of the readers, the patient is ineligible to complete the STOHFLA HL evaluation

a) BHLS

We want to find out what health care information is understood by patients and what is confusing to them, in order to help us understand what patients may find confusing.]

1) How often do you have problems learning about your medical condition because of difficulty understanding written information?
2) How confident are you filling out medical forms by yourself?
3) How often do you have someone help you read hospital material?

b) STOHFLA

Explain the purpose of this survey. Use the following text.

- We want to find out what health care information is understood by patients and what is confusing to them, in order to help us write things more clearly.

- Here are some medical instructions that you or anybody might see around the hospital. These instructions are in sentences that have some of the words missing.

- Where a word is missing, a blank line is drawn, and 4 possible words that could go in the blank appear just below it. I want you to figure out which of those 4 words should go in the blank, which word makes the sentence make sense.

- When you think you know which one it is, circle the letter in front of that word, and go on to the next one.

- When you finish the page, turn the page and keep going until you finish all the pages.

- Provide the patient a pen and a clipboard and ask them to complete the questionnaire (Form 10). If the patients ask for help with completing the questionnaire, instruct them to do the best they can and that you are not allowed to assist them. Only responses completed within 7 minutes are used in calculating a health literacy score. Record the start and stop time and any questions that were answered after 7 minutes had elapsed. Do not grade the STOFHLA until after the study visit is complete.

9. Administer VME Survey

Explain the purpose of this survey. Use the following text.

We are thinking of doing a study that would use interactive video modules to teach patients about skills to manage their health problems. Video modules include answering a few questions, followed by watching a video to teach about a health problem or skill, followed by a few questions to see if the video was helpful.

- D1.a. How likely would you be to use an **interactive** video-module education **program** after discharge to learn about skills to help your health problem?

- D1.b. How much do you agree with this statement: “I would be willing to use the video-module education but do not have access (do not have devices and/or internet) needed to use this education” (circle one)

- D1.c. How much to you agree with this statement: “I would be willing to use the video-module education but I do not think I would know how (be able to) even though I have a device (laptop/smartphone/tablet).

- D1.d1. How much do you agree with this statement: “ I have NO interest in using the video-module education after being discharged from the hospital.”
    i. d2. If not, please explain:

- D1.e. If you think you might use the video-module education, what device would you use to participate? (check all that apply)

- D1.f. How much do you agree with this statement: “I would be willing to use video-module education after discharge home if I was provided with **access** to (a computer, tablet, cell phone with internet (smartphone), etc))”

- D1.g. g. How much to you agree with this statement: “I would be willing to use a public resource such as a library, drugstore, grocery store, church or temple to be able to use the video-module education after being discharged from the hospital.”

10. **Collect patient contact information and provide patient $25 in cash for participating in this study.** Record patient's social security number and obtain patients signature that they received the patient incentive.

As part of Aim 1 procedures, we will ask if subjects may be interested in participating in a secondary study to test the feasibility of using VME at home. The consent form will contain information with a separate line to initial if the subject would like to participate.

5-10 subjects will be included in the home feasibility study. All subjects will be asked to use the VME on their own devices. They will be provided with an account to use the web-based VME, sent via email 3-5 days post-discharge. There will be an email reminder sent 7 days later if the VME has not yet been accessed. At 30-days post-discharge, the subject will receive a follow up phone call. This phone call will assess the following:

- Did you use the VME?
    - Yes
    - No
- How many times did you use the VME?
    - 0
    - 1-2
    - 3-5
    - Weekly
    - Daily
    - more than once per day
- If you did not use the VME, what were some reasons why?
- If you did use the VME:
    - Did the VME help you feel more confident about using your inhaler at home?
    - How long did it take you to use the VME at home?
    - Did you require help from anyone else in order to use the VME? Who helped you?
    - Is there any difference between the instructions in the video and those given by your doctor?
        - Yes
        - No
    - What device did you use to access the VME?
        - Computer
        - Tablet
        - Smartphone

OUTCOMES/MEASURES-AIM 1

The primary and secondary outcomes are listed below. Measures for each are described. Initial measurements (Vo; see Data Collection Schedule below) will be made prior to randomization; at V1, the trained educator will assess respiratory inhaler technique by asking the subject to demonstrate use of the MDI.

Primary outcome

The primary outcome will be inhaler technique. We will define inhaler technique in two ways:

a) Correct Use (i.e., >75% of steps correct)

b) Mastery (i.e., perfect technique, 100% steps correct)

This outcome will be measured at V1. We will test our hypothesis that patients will have improved inhaler technique post-VME compared to pre-VME. We will employ the same checklists used in the E-TRaIN study (IRB Protocol #11-0248, high reliability, kappa >0.94). These checklists will be used to measure the primary outcome: ability to use inhalers (defined as (a) correct use of inhaler: >9/12 steps for MDI; and (b) mastery of inhaler technique, i.e., perfect technique: 12/12 steps for MDI).

The **primary outcome** is the proportion of participants with MDI misuse post VME vs. pre VME. This will provide data on the short-term efficacy of VME.

Secondary outcomes

Secondary outcomes will include:

a) Symptom burden (i.e., BORG, ASSI, and CBSQ) at V1 and V2. Patient reported outcomes will be measured using a modified Borg dyspnea scale (Borg), Asthma Symptom Severity Index (ASSI), Chronic Bronchitis Symptom Questionnaire (CBSQ).
b) quality of life (QOL),[20]
c) and self-efficacy of inhaler technique.[8,10]

The efficacy of VME with regards to these secondary outcomes will employ simple univariate and/or multivariate logistic regression, generalized estimating equations, or generalized non-linear mixed models, as appropriate. For each of these outcomes, we will evaluate whether health literacy level modifies the effectiveness of VME by including an interaction term in the regression model. Model assumptions will be checked. A two-tailed p-value less than 0.05 will define statistical significance.

DATA COLLECTION SCHEDULE-AIM 1

	Inpatient Visit	
**VISIT**	**V0-V1**	
Visit duration (minutes)	60 (total for V0 to V1)	
Visit location	Inpatient	
Visit type	In-person	
Eligibility evaluation	■	
Consent	■	
Self Efficacy: Self-reported inhaler technique	■	
Patient demonstration of inhaler	■	■

technique		
Vision Screen		
Health Literacy	■	
VME Survey	■	
Spirometry before and after bronchodilator	■	
Review of medications	■	
Medication count/weigh		
Questionnaire about symptom burden, respiratory exacerbations, acute care utilization	■	
Discharge Questionnaire		■

Vo: initial assessment V1: after instruction V2: 30 days post-discharge follow-up

This study will require 2-6 months:

1. Pilot test interactive video round 1 (2 months)
2. Pilot test interactive video round 2 (2 months) if changes to VME needed
3. Pilot test interactive video round 3 (2 months) if changes to VME needed
4. Data analyses and preparation of final report (2 months).

The research will take place on the inpatient wards of UCM.

ANALYSES-AIM 1

Analysis plan. We will use means, medians, proportions, scatterplots, and histograms to describe the data. We will test for group differences using the *t*-test, the Wilcoxon rank sum test, Pearson's chi-squared test, or Fisher's exact tests as appropriate. We will adhere to the intention-to-treat principle when testing for differences by intervention type. The **primary outcome** will be correct inhaler technique; chi-squared tests will be used to compare post VME vs. pre VME prevalence of inhaler misuse (<75% of steps correct). **Secondary outcomes** will include self-efficacy for respiratory inhalers, symptom burden (i.e., BORG and CSS), baseline self-reported utilization of health care services for respiratory exacerbations, basic disease knowledge, and QOL. For each of these outcomes, we will evaluate whether patient characteristics (vision, age, and/or health literacy level) modifies the effectiveness of VME by evaluating interactions in the regression model. A two-tailed p-value less than 0.05 will define statistical significance.

SPECIFIC AIM 2

This will be a randomized, controlled, non-inferiority clinical trial conducted of patients admitted to the University of Chicago. Adults admitted with a history of Asthma or COPD will be eligible

to participate in this study. Upon receiving consent, participants will be randomized to either the VME or TTG. Inhaler technique will be measured prior to hospital discharge before and after education (VME or TTG). This will provide data on the short-term effectiveness of the interventions. Retention will be examined at 30 days after hospital discharge.

ELIGIBILITY CRITERIA

The goal of patient selection is to enroll adults admitted with a history of physician-diagnosed Asthma or COPD.

Inclusion criteria

1. Age 18 years and older
2. Admission to the inpatient medical service and surgical service
3. Physician-diagnosed Asthma or COPD. We will enroll patients even if the primary reason for admission is not Asthma or COPD (e.g., patients admitted for heart failure, but with a physician diagnosis of COPD are eligible).
4. Physician plans to discharge patients home a metered dose inhaler (MDI; e.g., albuterol)

Exclusion criteria

1. Currently in an intensive care unit
2. Physician declines to provide consent
3. Patient unable to provide consent (e.g., history of cognitive impairment, unable to understand English) or declines to provide consent
4. Previous participant in this study

RECRUITMENT

We will employ the same recruitment plan as in the protocol for our Effectiveness of interventions to Teach Respiratory Inhaler Technique (E-TRaIN) study (Protocol #11-0248). In summary, admission logs (EPIC) at the University of Chicago will be examined each day to screen for potentially eligible patients. The treating physician will be contacted for verbal assent using standardized text. Among patients whose physician provides assent, medical records will be reviewed to ascertain eligibility and informed consent will be sought from eligible patients. A total of 142 patients will be recruited at the University of Chicago Medical Center over 15 months.

TREATMENT CONDITIONS

1. **Teach-to-Goal (TTG):** During inpatient admission, participants assigned to the TTG conditon will be provided with an intensive, iterative education and evaluation strategy that consists of the following steps: First, participants will undergo an initial assessment of respiratory inhaler technique for MDI (and a Diskus® device if they are also prescribed medication using the device, i.e., fluticasone/salmeterol [Advair Diskus ®]) using standardized device-specific checklists (Form 1) that we have developed and used in the SARI study (Protocol #15729A), in TURI (#16900A), and in E-TRaIN (#11-0248). This will be followed by the educational intervention whereby participants will then be taught using verbal instructions and then by demonstration by a trained educator regarding the appropriate use of the MDI (and, if applicable, fluticasone/salmeterol (Advair Diskus®) using standardized written instructions (Forms 2a and 2b). Next the participants will be asked to demonstrate how they use their respiratory inhalers; their post-TTG technique will be graded using the same checklists as the initial assessment. The participants will receive additional rounds of instruction following this assessment if

needed (e.g., if the participant did not achieve mastery of the MDI or Diskus® after one round of teaching). Although initial pilot work showed that two rounds was sufficient, more recent data indicates that some participants require > 2 rounds; since the essence of this education technique is to teach-to-goal, and this requires only a few additional minutes (maxium) we will attempt to teach-to-goal. The patient's hospital physician (attending physician or their designee) will be provided information about the patients inhaler technique for both inhalers after education; this will provide an opportunity for the patient to receive addition instructions by the clinical team if needed. All instructions will be provided by a dedicated trained educator; all assessments will be performed by a second research assisstant (trained assessor). The trained assessor will be masked to treatment assignment (see below).[15]

2. **Video Module Education (VME):** The RE will provide participants with a tablet device and will demonstrate how to access VME and complete the pre/post e-learning assessments. The RE will provide technical support but will neither participate directly in the education nor help with the self-assessments. The participants will first complete the pre-assessment e-learning tool on the tablet. They will then watch the video instruction that will provide a complete demonstration with verbal instructions on correct inhaler technique. Next the participants will complete the post-assessment e-learning tool. Based on participants' performance, they will be directed to further tailored video-instruction. The cycle of self-assessment and video instruction will continue until sufficient mastery has been achieved.

<u>Other considerations:</u>

1. *Masking:* To minimize risk of biased measurements of inhaler technique, the trained assessor will be masked to the treatment condition. Specifically, the trained assessor will not be informed about the treatment assignment, will remain outside the patients' room during the educational intervention (and the room door will remain closed during the intervention), and the trained educator will be instructed not to inform the assessor about the treatment condition. We have successfully used similar procedures for masking the trained assessor in other IRB-approved studies (see E-TRaIN, IRB # 11-0248).

2. *Patients being discharged home on a MDI and a Diskus will receive VME or TTG interventions for both devices.* If randomized to the TTG intervention, then TTG will be used to teach MDI and Diskus use; likewise if randomized to the VME intervention, then VME will be used to teach MDI and Diskus use. Based on results of screening data from the TURI study (IRB #16900A), we expect that about 10-20% of participants (10 to 20 participants enrolled in this study) will be prescribed both devices. We would like to include this option to teach two different devices (in patients prescribed both devices) to collect preliminary data about the feasibility of VME for MDI vs. TTG for Diskus; these data would be valuable as we prepare for future studies.

PROTOCOL FOR BOTH GROUPS:

Following the screening procedures above, we will use the following steps:

11. **Obtain assent from treating physician prior to approaching patients hospitalized with asthma or COPD.** The treating physician will be contacted by a research assistant for verbal assent using standardized text (Form 3).

12. **Obtain written informed consent from patient using standardized text.** One hundred eighteen participants (N=59 TTG condition; N=59 VME condition) will be enrolled in this study.

13. **Optimize room conditions**
    - Room well lit (turn on overhead lights if necessary)
    - Patient preferably sitting up
    - Private setting (close door to patient room)
    - Interviewer should directly face patient
    - Patient should use reading glasses if they use one

14. **Assess patient's respiratory symptoms/morbidity (Forms 4-5-6), which includes the** Borg symptom score, Asthma Symptom Severity Index (ASSI), Chronic Bronchitis Symptom Questionnaire, COPD Severity Score (CSS), Airway Questionnaire (AQ-20), COPD Helplessness Index (CHI) and demographic and other clinical information.

15. **Assess patients' self-efficacy regarding respiratory inhaler use (Form 7)**

16. **Assess lung function. Perform spirometry pre and post-bronchodilator (after 2 puffs albuterol MDI via spacer) using portable KoKo spirometer. (Forms 8a and 8b).** These data will provide an objective measure of disease severity at the time of the hospitalization. We have previously demonstrated that this population has poor perception of airflow obstruction and spirometry is feasible during the inpatient setting.[16]

17. **Assess patient's inhaler technique using Inhaler checklists (Form 1;** for a MDI and, if applicable, Diskus®) by the trained assessor.

18. **Assess health literacy. Check vision (Form 9). Ask brief health literacy screen questions (Form 10a) for all participants and a**dminister STOHFLA questionnaire (Form 10b) if the patient's visual acuity is at least 20/50 in one eye (Check the patient's visual acuity in each eye using the Snellen Pocket Eye Chart (Form 11), otherwise, the patient is ineligible to complete the STOHFLA HL evaluation (will complete the BHLS questions).

    a) BHLS
    We want to find out what health care information is understood by patients and what is confusing to them, in order to help us understand what patients may find confusing.
    1) How often do you have problems learning about your medical condition because of difficulty understanding written information?
    2) How confident are you filling out medical forms by yourself?
    3) How often do you have someone help you read hospital material?

    b) STOHFLA
    Assess health literacy. Check vision (Form 9). Administer STOHFLA questionnaire (Form 10) if the patient's visual acuity is at least 20/50 in one eye (Check the patient's visual acuity in each eye using the Snellen Pocket Eye Chart (Form 11), otherwise, the

patient is ineligible to complete the HL evaluation. Explain the purpose of this survey. Use the following text.

- We want to find out what health care information is understood by patients and what is confusing to them, in order to help us write things more clearly.

- Here are some medical instructions that you or anybody might see around the hospital. These instructions are in sentences that have some of the words missing.

- Where a word is missing, a blank line is drawn, and 4 possible words that could go in the blank appear just below it. I want you to figure out which of those 4 words should go in the blank, which word makes the sentence make sense.

- When you think you know which one it is, circle the letter in front of that word, and go on to the next one.

- When you finish the page, turn the page and keep going until you finish all the pages.

- Provide the patient a pen and a clipboard and ask them to complete the questionnaire (Form 10). If the patients ask for help with completing the questionnaire, instruct them to do the best they can and that you are not allowed to assist them. Only responses completed within 7 minutes are used in calculating a health literacy score. Record the start and stop time and any questions that were answered after 7 minutes had elapsed. Do not grade the STOFHLA until after the study visit is complete.

19. **Obtain randomization assignment from the study biostatistician.** Randomization will be block-stratified by level of health literacy to ensure an equal number of subjects within each stratum assigned to TTG and VME Patients will then receive either the TTG or VME intervention by the trained educator.

20. **Collect patient contact information and provide patient $25 in cash for participating in this study as well as parking pass/bus pass for their follow-up visit.** Record patient's social security number and obtain patients signature that they received the patient incentive.

    1. **Provide patients an appointment for an in-person interview 30 days after discharge (patients will be given a letter with their appointment time).** At the in-person interview, we will ask patients to complete a short interviewer-administered questionnaire about patient's respiratory symptoms/morbidity/self-reported ability to use MDI and Diskus® inhalers, quality of life (Forms 4 and 12). We will also assess lung function (spirometry) and use of inhalers (MDI and/or Diskus). Upon completing this portion the participant will receive $50 as well as parking pass/bus pass for their next follow-up visit.

OUTCOMES/MEASURES-AIM 2

The primary and secondary outcomes are listed below. Measures for each are described. Initial measurements (Vo; see Data Collection Schedule below) will be made prior to

randomization; at V1, the trained educator will assess respiratory inhaler technique by asking the subject to demonstrate use of the MDI (and Diskus® if to be discharged home on fluticasone/salmeterol). Follow-up assessment, V2, will take place at 30 days (+/-7 days) post-hospital discharge.

Primary outcome

The primary outcome will be inhaler technique. We will define inhaler technique in two ways:

c) Correct Use (i.e., >75% of steps correct)
d) Mastery (i.e., perfect technique, 100% steps correct)

This outcome will be measured at V1 and V2. We will test our hypothesis that patients will have a reduced proportion of respiratory events when taught effective inhaler technique with TTG compared to BI education separately for the MDI and Diskus® devices after hospital discharge. We will employ the same checklists used in the E-TRaIN study (IRB Protocol #11-0248, high reliability, kappa >0.94). These checklists will be used to measure the primary outcome: ability to use inhalers (defined as (a) correct use of inhaler: >9/12 steps for MDI; >8/10 steps for Diskus®; and (b) mastery of inhaler technique, i.e., perfect technique: 12/12 steps for MDI; 10/10 steps for Diskus®).

The **primary outcome** is the proportion of participants with MDI misuse post VME vs. TTG education. This will provide data on the short-term effectiveness of the interventions. Longer-term retention (durability) will be examined 30 days post-hospital discharge using an intention-to-treat analysis. We have powered on this binary variable. [Table 1] However, because we recognize that there is more information about which steps are less critical and those that are more critical, I will also work with Robert Gibbons, a nationally recognized expert in biostatistics, to model a mixed effects ordinal regression model with random intercepts and time effects within correlation of each participant with terms for mode (VME and TTG), as well as interaction with time, to determine the predicted probability in each category for VME and TTG of how prevalence of misuse changes over time.[17] **Mediators include** health literacy[18] and vision level[19] as I have previously identified them as important mediators of baseline inhaler misuse and/or ability to learn correct technique.[8,10]

Secondary outcomes

Secondary outcomes will include:

d) Change in self-efficacy for respiratory inhalers (i.e., V0-V2). We will ask all patients to state how strongly they agree or disagree with the following statement "I am confident that I know how to use [insert inhaler name] correctly" for both MDI and Diskus®. The subject will be shown an example of each inhaler. Each item will be scored on a 5-point Likert scale (strongly disagree to strongly agree); the mean score will be used to measure self-efficacy. Cronbach's alpha will be calculated to evaluate internal consistency.
e) Symptom burden (i.e., BORG, ASSI, and CBSQ) at V1 and V2. Patient reported outcomes will be measured using a modified Borg dyspnea scale (Borg), Asthma Symptom Severity Index (ASSI), Chronic Bronchitis Symptom Questionnaire (CBSQ).
f) Acute care utilization for respiratory exacerbations at V2. Data will be collected using interviewer administrated surveys. *Respiratory events* are defined as: physician, emergency room or hospital visit, or death. This composite endpoint is due to small sample size.

The comparative effectiveness of TTG versus VME with regards to these secondary outcomes will employ simple univariate and/or multivariate logistic regression, generalized estimating equations, or generalized non-linear mixed models, as appropriate. For each of

these outcomes, we will evaluate whether health literacy level modifies the effectiveness of VME (Vs. TTG) by including an interaction term in the regression model. Model assumptions will be checked. Use of two inhalers will also provide the opportunity to evaluate if the comparative effectiveness of VME (vs. TTG) varies by device. A two-tailed p-value less than 0.05 will define statistical significance. The sample size calculations demonstrate that N=59 per group will provide 80% power for an intention-to-treat analysis to test for non-inferiority of VME vs. TTG.

Secondary Outcomes include quality of life (QOL),[20] patient reported outcomes,[20-21] lung function, utilization of health care services (outpatient visits, ED and hospital admissions, deaths), and self-efficacy of inhaler technique.[8,10] We will also evaluate the differential effects of VME vs. TTG by participant characteristics (e.g., age, health literacy). Finally we will evaluate the preliminary comparative durability of VME vs. TTG at 30 days post-hospital discharge. We will perform all tests separately for both MDI and Diskus devices.

Subjects in Aim 2 procedures will also be offered participation in the VME at Home sub-study. We will ask if subjects may be interested in participating in a secondary study to test the feasibility of using VME at home. The consent form will contain information with a separate line to initial if the subject would like to participate. If they initial this line, they will sign the separate VME at Home sub-study consent document.

5-10 subjects will be included in the home feasibility study. All subjects will be asked to use the VME on their own devices. They will be provided with an account to use the web-based VME, sent via email 3-5 days post-discharge. There will be an email reminder sent 7 days later if the VME has not yet been accessed. At 30-days post-discharge, the subject will receive a follow up phone call. This phone call will assess the following:

- Did you use the VME?
    - Yes
    - No
- How many times did you use the VME?
    - 0
    - 1-2
    - 3-5
    - Weekly
    - Daily
    - more than once per day
- If you did not use the VME, what were some reasons why?
- If you did use the VME:
    - Did the VME help you feel more confident about using your inhaler at home?
    - How long did it take you to use the VME at home?
    - Did you require help from anyone else in order to use the VME? Who helped you?
    - Is there any difference between the instructions in the video and those given by your doctor?
        - Yes
        - No
    - What device did you use to access the VME?
        - Computer
        - Tablet
        - Smartphone

DATA COLLECTION SCHEDULE-AIM 2

VISIT	Inpatient Visit V0-V1		30 day FU V2
Visit duration (minutes)	60 (total for V0 to V1)		60
Visit location	Inpatient		Outpatient
Visit type	In-person		In-person
Eligibility evaluation	■		
Consent	■		
Randomization	■		
Self Efficacy: Self-reported inhaler technique	■		■
Patient demonstration of inhaler technique	■	■	■
Vision Screen	■		
Health Literacy	■		
Spirometry before and after bronchodilator	■		■
Review of medications	■		■
Medication count/weigh			■
Questionnaire about symptom burden, respiratory exacerbations, acute care utilization	■		■
Discharge Questionnaire		■	

Vo: initial assessment V1: after instruction V2: 30 days post-discharge follow-up

This study will require 24 months:

5. Develop/pilot test interactive video (6 month)
6. Training of personnel (1 month)
7. Begin recruitment (1-3 participants/week, for 15 months)
8. Complete follow-up of last enrollee (1 month after last enrolled participant)
9. Data analyses and preparation of final report (2 months).

The research will take place on the inpatient wards of UCM.

ANALYSES-AIM 2

Analysis plan. We will use means, medians, proportions, scatterplots, and histograms to describe the data. We will test for group differences using the *t*-test, the Wilcoxon rank sum test, Pearson's chi-squared test, or Fisher's exact tests as appropriate. We will adhere to the intention-to-treat principle when testing for differences by intervention type. The **primary outcome** will be correct inhaler technique; chi-squared tests will be used to compare post VME vs. post TTG prevalence of inhaler misuse (<75% of steps correct). **Secondary outcomes** will include change in self-efficacy for respiratory inhalers, symptom burden (i.e., BORG and CSS),

self-reported utilization of health care services for respiratory exacerbations, basic disease knowledge, and QOL. The comparative effectiveness of VME versus TTG with regards to these secondary outcomes will employ simple univariate and/or multivariate logistic regression, as appropriate. For each of these outcomes, we will evaluate whether patient characteristics (vision, age, and/or health literacy level) modifies the effectiveness of VME versus TTG by evaluating interactions in the regression model. A two-tailed p-value less than 0.05 will define statistical significance. The sample size calculations (see below) demonstrate that n=59 per group will provide 80% power for an intention-to-treat analysis.

Sample size. (Table 1) The main outcome is correct MDI use (>75% steps correct). In my preliminary studies above, the proportion of participants with correct MDI use after TTG was 88-100% (n=98, n=50, respectively).[8,10] These data (expected proportion of correct MDI use of 0.95), along with a noninferiority effect size of -0.10, were used to calculate the sample size.[22] We will enroll 142 participants over 15 months and will have sufficient power (0.8) to determine if VME is non-inferior to TTG, at a significance level of p<0.05. We have experience recruiting participants and have been able to recruit up to 12 participants per month; therefore, we do not foresee any problems with recruiting this number of participants in this timeline.

Table 1: Sample Size per intervention (VME/TTG)

	**Power**				
**EFFECT SIZE**	**0.60**	**0.70**	**0.80**	**0.90**	**0.95**
**-0.05**	137	179	236	326	412
**-0.10**	35	45	59	82	103
**-0.15**	16	20	27	37	46

<u>Interim analyses:</u> After 50% of the target sample size have completed the study (59 participants have completed the study) or after 50% of the study period (~7 months), whichever occurs first, results of an interim analysis will be provided to a Data Safety and Monitoring Board (Drs. Dana Edelson, MD; Steve White, MD; Neda Laiteerapong, MD; physicians who are not study investigators). Investigators and research assistants will remain masked to the results of the interim analyses until the end of the study. The DSMB will be asked to consider issues related to study performance (enrollment rates, study completion rates) and differences in outcomes between groups and provide recommendations to the PI and the IRB whether to continue the study or terminate enrollment.

<u>BENEFITS AND RISKS AIMS1 AND 2</u>

Benefits

We are not aware of any potential benefits to study subjects.

Risks

Potential risks include:

1. The subject may be uncomfortable answering some interview questions. They can refuse to answer any question.

2. The lung function test is safe and is commonly used to measure the severity of COPD but can cause some minor chest soreness or lightheadedness; study test will be done by a trained member of the research staff with the subject in a seated position. To further minimize the risk

of adverse events with spirometry, we will not perform spirometry in subjects with SBP>180 or DBP>100 mm Hg.

3. Two puffs of albuterol (for post-bronchodilator spirometry) is generally well tolerated. We have not had adverse events with 2 puffs albuterol after enrolling >170 subjects in the SARI, COAT, and TURI studies (IRBs# 15729A, 14831A, 16900A). In some cases, however, 2 puffs of albuterol can cause jitteriness, anxiety, and tachyarrhythmias. To minimize this risk, we will withhold albuterol in subjects who are hospitalized with tachyarrhythmias or palpitations or report problems after using albuterol.

4. Long acting beta-2-agonists (salmeterol or formoterol). These medications are generally well-tolerated, but can have albuterol-like adverse effects; in addition, these agents have been associated with increase risk of asthma-related deaths.

5. Inhaled corticosteroids (fluticasone/budesonide). These medications are well tolerated, but can cause thrush, dysphonia, and other less common adverse effects.

6. Loss of confidentiality. To help ensure that patients' health information remains private, we will restrict access to data collected for our study to study personnel only (via use of password-protection and locked cabinets for study documents). Study ID numbers will be generated and will be used when discussing and/or reviewing data at study meetings. Also, study reports will report results in aggregate and not contain information that can be used to identify individual patients.

Data Safety Monitoring Plan-AIM 2

The PI will review monthly data reports reviewing recruitment, consent, intervention activities, adverse events, and follow-up. Although few, if any, intervention-related adverse events are anticipated in this education-related study, the study population is at risk for poor outcomes, including life-threatening exacerbations and death. The PI will comply with prompt reporting of any unanticipated events and the Data Safety and Monitoring Board (DSMB) (Dana Edelson, MD, Steve White, MD, Neda Laiteerapong, MD). The DSMB (see interim analysis above) will also review results of the interim and final analyses.

PATIENT INCENTIVES-AIM 1 AND 2

Participants will be provided $25 for completing the initial baseline interview (only interview for AIM 1, first interview for AIM 2) and $50 completing the post-hospital discharge in-person interview. Total possible monetary compensation is $25 for AIM 1 and $75 for AIM 2. For AIM 2, participants will also be provided parking passes or bus passes for their follow-up visits as we recognize that this cost burden should not fall on the participant for participating in our study. Monetary compensation will be reduced, based on the extent to which the participante completes the study. For both AIMs all tests for this study will be done free of charge.

BIBLIOGRAPHY

1. Morbidity and Mortality 2007 Chart Book on Cardiovascular, Lung, and Blood diseases. Available at: http://www.nhlbi.nih.gov/resources/docs/07-chtbk.pdf. Accessed June 06, 2012.

2. Jencks SF, Williams MV, Coleman EA. Rehospitalizations among patients in the Medicare fee-for-service program. N Engl J Med 2009;360(14):1418-1428. PMID: 19339721.

3. Hanania NA, Darken P, Horstman D, Reisner C, Lee B, Davis S, Shah T. The efficacy and safety of Fluticasone Propionate (250ug) /Salmeterol (50ug) combined in the Diskus® Inhaler for the treatment of COPD. Chest 2003;124:834-843. PMID: 12970006.

4. Celli BR, Thomas NE, Anderson JA, Ferguson GT, Jenkins CR, Jones PW, Vestbo J, Knobil K, Yates JC, Calverley PMA. Effect of pharmacotherapy on rate of decline of lung function in chronic obstructive pulmonary disease. Results from the TORCH Study. Am J Resp Crit Care Med 2008;178:332-338. PMID: 18511702.

5. Melani, AS, Bonavia M, Cilenti V, Cinti C, Lodi M, Martucci P, Serra M, Scichilone N, Sestini P, Aliani M, Neri M, on behalf of the Gruppo Educazionale Associazione Italiana Penumologi Ospedalieri (AIPO). Inhaler mishandling remains common in real life and is associated with reduced disease control. Respir Med 2011;105:930-938. PMID: 21367593.

6. Dolovich MB, Ahrens RC, Hess DR, Anderson P, Dhand R, Rau JL, Smaldone GC, Guyatt G. Device selection and outcomes of aerosol therapy: Evidence-based guidelines: American College of Chest Physicians/American College of Asthma, Allergy, and Immunology Chest. 2005;127:335-71. PMID: 21367593.

7. The Global initiative for Chronic Obstructive Lung Disease (GOLD) Guidelines: Available at: http://www.goldcopd.com/. Accessed June 6, 2012.

7.5 Guidelines for the Diagnosis and Management of Asthma (EPR-3). Available at: https://www.nhlbi.nih.gov/guidelines/asthma/. Accessed June 6, 2012.

8. Press VG, Shah LM, Lewis S, Ivy K, Charbeneau J, Mazurek A, Badlani S, Naureckas E, Krishnan JA. Misuse of respiratory inhalers in hospitalized patients with asthma or COPD. J Gen Intern Med 2012;26(6), 635-642. PMID: 21249463 PMCID: PMC3101982.

9. Cohen DJ, Clark EC, Lawson PJ, Casucci BA, Flocke SA. Identifying teachable moments for health behavior counseling in primary care. Patient Educ Couns 2011;85:e8-e15. PMID: 21183305

10. Press VG, Arora VM, Lewis SL, Charbeneau J, Starkey J, Naureckas ET, Krishnan JA. Teaching the use of respiratory inhalers to hospitalized patients with asthma or COPD: A randomized trial (TURI). J Gen Intern Med May 2012. [Epub ahead of print.] PMID: 22592354.

11. Paasche-Orlow MK, Riekert KA, Bilderback A, Chanmugam A, Hill P, Rand CS. Tailored education may reduce health literacy disparities in asthma self-management. Am J Respir Crit Care Med 2005;172(8):980-986. PMID: 16081544 PMCID: PMC2718412.

12. Virchow JC, Crompton GK, Negro RD, Pedesen S, Magnan A, Seidenberg J, Barnes PJ. Importance of inhaler devices in the management of airway disease. Respir Med 2008;102:10-19. PMID: 17923402.

13. Jack BW, Chetty VK, Anthony D, Greenwalk JL, Sanchez GM, Johnson AE, Forsythe SR, O'Donnell JK, Paasche-Orlow MK, Manasseh C, Martin S, Culpepper L. A Reengineered Hospital Discharge Program to Decrease Rehospitalization. A Randomized Trial. Ann Intern Med 2009;150(3):178:187. PMID: 19189907. PMCID: PMC2738592.

14. Gerber BS, Brodsky IG, Lawless KA, Smolin LI, Arozullah AM, Smith EV, Berbaum ML, Heckerling PS, Eiser AR. Implementation and evaluation of a low-literacy diabetes education computer multimedia application. Diabetes Care 2005;28(7):1574-1580. PMID: 15983303.

15. Press VG, Shah LM, Lewis SL, Ivy K, Charbeneau JT, Mazurek A, Krishnan JA. Teach-to-goal intervention to teach respiratory inhaler technique in patients hospitalized for asthma or COPD. Abstract, accepted for poster presentation, Society of General Internal Medicine Annual Meeting May 2009.

16. Davis SQ, Permutt Z, Permutt S, Naureckas ET, Bilderback AL, Rand CS, Stein BD, Krishnan JA. Perception of airflow obstruction in patients hospitalized for acute asthma. *Annals of Asthma, Allergy, and Immunology.* 2009*;102(6)455-61.*

17. Hedeker D, Gibbons RD. Longitudinal Data Analysis. New Jersey: John Wiley & Sons; 2006.

18. Nurss JR, Parker RM, Williams MV, Baker, DW. Short Test of Functional Health Literacy in Adults (STOFHLA). Hartford: Peppercorn Books & Press; 2001.

19. Snellen Eye Chart: A Description and Explanation. Available at: http://precision-vision.com/index.cfm/feature/24_149/snellen-eye-chart---a-description-and-explanation.cfm. Accessed June 6, 2012.

20. Kendrick KR, Baxi SC, Smith RM. Usefulness of the modified 0-10 Borg scale in assessing the degree of dyspnea in patients with COPD and asthma. J Emerg Nurs 2002;26:216-222. PMID: 10839848.

21. Eisner MD, Trupin L, Katz PP, Yelin EH, Earnest G, Balmes J, Blanc PD. Development and validation of a survey-based COPD severity score. Chest 2005;127:1890-1897. PMID: 15947299.

22. Hung HMJ, Wang SJ, O'Neill R. A regulatory perspective on choice of margin and statistical inference issue in non-inferiority trials. Biom J 2005;47(1):28-36. PMID: 16395994.

Version: 09 June 2016

Protocol Title: Video vs. TTG Respiratory inhaler technique Assessment and InstructioN (V-TRaIN)

University of Chicago
PI: Valerie G. Press, MD, MPH

Co-Investigators: David Meltzer, MD, PhD, Vineet Arora, MD, MAPP, Ted Naureckas, MD

Research personnel: John Kim, Nicole Woodrick, Nicole Twu, Ashley Hull, Ashley Snyder

BACKGROUND

Asthma and Chronic obstructive pulmonary disease (COPD) results in over a million hospitalizations in the United States annually[1] and COPD is the third leading cause of 30-day re-hospitalizations.[2] Clinical trials have established the efficacy of treatments primarily dispensed via respiratory inhaler devices that reduce morbidity and health care utilization if they are used correctly.[3-4] Unfortunately, the effectiveness of these medications in real-world settings is limited by the fact that patients often do not use inhalers correctly.[5-6] Current guidelines recommend assessing and teaching inhaler technique at all health care encounters, including hospitalization.[77.5] My work has found that over 75% of hospitalized patients in an urban, predominantly underserved population misuse their respiratory inhalers, highlighting a missed opportunity to educate these patients with high potential to benefit.[8] Hospitalization, therefore, provides a potential 'teachable moment' to correct this misuse.[9] My preliminary data indicate that one strategy, in-person teach-to-goal (TTG), is effective in teaching hospitalized patients proper inhaler technique and is more effective than simple verbal instruction.[10]

While TTG is a promising method to improve care for patients who use inhalers, several limitations prevent widespread adoption. First, TTG relies on in-person assessment and education, as well as training and monitoring instructors to ensure fidelity, making it time-consuming and costly. Also, because a single educational session does not ensure long-term retention,[11-12] post-discharge reinforcement may be needed, which may be impractical with in-person TTG. One potential method to surmount TTG's limitations is use of interactive video module education (VME), a method that has been used for health education in other clinical contexts.[13-14] Through iterative self-assessments and video-demonstrations on a tablet computer, VME has the potential to be less costly, maintain fidelity, and be more easily extended into the post-discharge setting than in-person TTG. However, certain questions remain about VME. It is unclear whether VME will yield similar results when compared to TTG, or whether urban, underserved patients will have the ability to, and be willing to use, VME in the post-discharge setting. Therefore, before widespread implementation of VME, it is critical to rigorously develop and test VME for inhaler education in the hospital setting. Ultimately, it will also be important to understand patients' ability and willingness to use post-discharge VME for educational reinforcement to allow for this strategy to transition patients across care settings from hospital to home. Through these funded projects, I will acquire critical data I need to develop R-01 studies focused on improving self-management for patients with obstructive pulmonary disease across care transitions.

<u>We hypothesize that interactive VME will lead to non-inferior rates of ability to demonstrate correct inhaler use compared to rates with TTG among hospitalized patients with Asthma or COPD.</u>

The specific aims to test this hypothesis are:

1. **To develop and iteratively test VME to teach correct use of metered dose inhaler (MDIs) and dry-powder Diskus® devices to hospitalized patients with Asthma or COPD.**

   We partnered with Click to Play Media™ (C2P) and Smart Sparrow to develop VME modules that (self)-assess and teach respiratory inhaler technique to hospitalized participants. We have tested the VME in focus groups (IRB #13-1139). Now that the VME is developed we will iteratively test (n=30-40) the VME strategy (up to 3 rounds (90-120 total)) to obtain important patient feedback, preferences and preliminary efficacy estimates for the module.

   We hypothesize that participants' post-VME inhaler technique will be significantly better than their pre-VME inhaler technique.

2. **To determine if the proportion of hospitalized patients with Asthma or COPD who demonstrate correct inhaler technique after receiving VME is not significantly less than (non-inferior to) the proportion who demonstrate correct inhaler technique after receiving in-person TTG.**

   To accomplish this aim we will build on our prior work with developing and evaluating the in-person TTG strategy to develop a parallel VME strategy that incorporates guideline-recommended components of assessment and instruction. We will conduct a behavioral randomized clinical trial to evaluate the relative effectiveness and durability of VME versus in-person TTG education on the ability of hospitalized patients to demonstrate correct MDI and Diskus® use. Secondarily, we will assess whether there is variation in comparative effectiveness by patient characteristics including age, gender, and health literacy level, among other important factors.

We hypothesize that interactive VME will lead to non-inferior rates of ability to demonstrate correct inhaler use compared to rates with TTG among hospitalized patients with Asthma or COPD.

SPECIFIC AIM 1

This will be a pre/post study of inpatients admitted to the University of Chicago. Adults admitted with a history of physician diagnosed COPD or asthma will be eligible to participate in this study. Upon receiving consent, participants will enrolled. Inhaler technique will be measured pre and post VME. This will provide data on the short-term efficacy of the VME.

Inclusion criteria:

1. Age 18 years and older
2. Admission to the inpatient medical service and surgical service
3. Physician-diagnosed COPD or asthma. We will enroll patients even if the primary reason for admission is not COPD or asthma (e.g., patients admitted for heart failure, but with a physician diagnosis of COPD are eligible).
4. Physician plans to discharge patients home a metered dose inhaler (MDI; e.g., albuterol)

Exclusion criteria

1. Currently in an intensive care unit
2. Physician declines to provide consent
3. Patient unable to provide consent (e.g., history of cognitive impairment, unable to understand English) or declines to provide consent
4. Less than 20/50 vision in both eyes

RECRUITMENT

Admission logs (EPIC) at the University of Chicago will be examined each day to screen for potentially eligible patients. The treating physician will be contacted for verbal assent using standardized text. Among patients whose physician provides assent, medical records will be reviewed to ascertain eligibility and informed consent will be sought from eligible patients. Sixty to ninety participants (approximately 30 per iterative round of VME up to 3 rounds) will be recruited at the University of Chicago Medicine over 2-6 months.

Video Module Education (VME):

The RE will provide participants with a tablet device and will demonstrate how to access VME and complete the pre/post e-learning assessments. The RE will provide technical support but will neither participate directly in the education nor help with the self-assessments. The participants will first complete the pre-assessment e-learning tool on the tablet. They will then watch the video instruction that will provide a complete demonstration with verbal instructions on correct inhaler technique. Next the participants will complete the post-assessment e-learning tool. Based on participants' performance, they will be directed to further tailored video-instruction. The cycle of self-assessment and video instruction will continue until sufficient mastery has been achieved.

Following the screening procedures above, we will use the following steps:

1. **Obtain assent from treating physician prior to approaching patients hospitalized with asthma or COPD.** The treating physician will be contacted by a research assistant for verbal assent using standardized text (Form 3).

2. **Obtain written informed consent from patient using standardized text.**

3. **Optimize room conditions**
   - Room well lit (turn on overhead lights if necessary)
   - Patient preferably sitting up
   - Private setting (close door to patient room)
   - Interviewer should directly face patient
   - Patient should use reading glasses if they use one

4. **Assess patient's respiratory symptoms/morbidity (<u>Forms 4-5-6</u>), which includes the** Borg symptom score, Asthma Symptom Severity Index (ASSI), Chronic Bronchitis Symptom Questionnaire, COPD Severity Score (CSS), Airway Questionnaire (AQ-20), COPD Helplessness Index (CHI) and demographic and other clinical information.

5. **Assess patients' self-efficacy regarding respiratory inhaler use (Form 7)**

6. **Assess lung function. Perform spirometry pre and post-bronchodilator (after 2 puffs albuterol MDI via spacer) using portable KoKo spirometer. (Forms 8a and 8b).** These data will provide an objective measure of disease severity at the time of the hospitalization. We have previously demonstrated that this population has poor perception of airflow obstruction and spirometry is feasible during the inpatient setting.[16]

7. **Assess patient's inhaler technique using Inhaler checklists (Form 1;** for a MDI) by the trained assessor.

8. **Assess health literacy. Check vision (Form 9). Ask brief health literacy screen questions (Form 10a) for all participants and a**dminister STOHFLA questionnaire (Form 10b) if the patient's visual acuity is at least 20/50 in one eye (Check the patient's visual acuity in each eye using the Snellen Pocket Eye Chart (Form 11). If the patient's visual acuity is not 20/50 in at least one eye, administer readers.

   - 4 strengths of glasses: 2.00, 2.25, 2.75, and 3.25
   - Start with 2.00, and repeat the vision screen, with each eye separately
   - If visual acuity of 20/50 is reached in at least one eye, the patient is eligible to complete the STOHFLA HL evaluation using the readers
   - If vision is not corrected after trying all of the strengths of the readers, the patient is ineligible to complete the STOHFLA HL evaluation

a) BHLS

We want to find out what health care information is understood by patients and what is confusing to them, in order to help us understand what patients may find confusing.]

1) How often do you have problems learning about your medical condition because of difficulty understanding written information?
2) How confident are you filling out medical forms by yourself?
3) How often do you have someone help you read hospital material?

b) STOHFLA

Explain the purpose of this survey. Use the following text.

- We want to find out what health care information is understood by patients and what is confusing to them, in order to help us write things more clearly.

- Here are some medical instructions that you or anybody might see around the hospital. These instructions are in sentences that have some of the words missing.

- Where a word is missing, a blank line is drawn, and 4 possible words that could go in the blank appear just below it. I want you to figure out which of those 4 words should go in the blank, which word makes the sentence make sense.

- When you think you know which one it is, circle the letter in front of that word, and go on to the next one.

- When you finish the page, turn the page and keep going until you finish all the pages.

- Provide the patient a pen and a clipboard and ask them to complete the questionnaire (Form 10). If the patients ask for help with completing the questionnaire, instruct them to do the best they can and that you are not allowed to assist them. Only responses completed within 7 minutes are used in calculating a health literacy score. Record the start and stop time and any questions that were answered after 7 minutes had elapsed. Do not grade the STOFHLA until after the study visit is complete.

9. Administer VME Survey

Explain the purpose of this survey. Use the following text.

We are thinking of doing a study that would use interactive video modules to teach patients about skills to manage their health problems. Video modules include answering a few questions, followed by watching a video to teach about a health problem or skill, followed by a few questions to see if the video was helpful.

- D1.a. How likely would you be to use an **interactive** video-module education **program** after discharge to learn about skills to help your health problem?

- D1.b. How much do you agree with this statement: "I would be willing to use the video-module education but do not have access (do not have devices and/or internet) needed to use this education" (circle one)

- D1.c. How much to you agree with this statement: "I would be willing to use the video-module education but I do not think I would know how (be able to) even though I have a device (laptop/smartphone/tablet).

- D1.d1. How much do you agree with this statement: " I have NO interest in using the video-module education after being discharged from the hospital."
    i. d2. If not, please explain:

- D1.e. If you think you might use the video-module education, what device would you use to participate? (check all that apply)

- D1.f. How much do you agree with this statement: "I would be willing to use video-module education after discharge home if I was provided with **access** to (a computer, tablet, cell phone with internet (smartphone), etc))"

- D1.g. g. How much to you agree with this statement: "I would be willing to use a public resource such as a library, drugstore, grocery store, church or temple to be able to use the video-module education after being discharged from the hospital."

10. **Collect patient contact information and provide patient $25 in cash for participating in this study.** Record patient's social security number and obtain patients signature that they received the patient incentive.

As part of Aim 1 procedures, we will ask if subjects may be interested in participating in a secondary study to test the feasibility of using VME at home. The consent form will contain information with a separate line to initial if the subject would like to participate.

5-10 subjects will be included in the home feasibility study. All subjects will be asked to use the VME on their own devices. They will be provided with an account to use the web-based VME, sent via email 3-5 days post-discharge. There will be an email reminder sent 7 days later if the VME has not yet been accessed. At 30-days post-discharge, the subject will receive a follow up phone call. This phone call will assess the following:

- Did you use the VME?
    - Yes
    - No
- How many times did you use the VME?
    - 0
    - 1-2
    - 3-5
    - Weekly
    - Daily
    - more than once per day
- If you did not use the VME, what were some reasons why?
- If you did use the VME:
    - Did the VME help you feel more confident about using your inhaler at home?
    - How long did it take you to use the VME at home?
    - Did you require help from anyone else in order to use the VME? Who helped you?
    - Is there any difference between the instructions in the video and those given by your doctor?
        - Yes
        - No
    - What device did you use to access the VME?
        - Computer
        - Tablet
        - Smartphone

OUTCOMES/MEASURES-AIM 1

The primary and secondary outcomes are listed below. Measures for each are described. Initial measurements (Vo; see Data Collection Schedule below) will be made prior to randomization; at V1, the trained educator will assess respiratory inhaler technique by asking the subject to demonstrate use of the MDI.

Primary outcome

The primary outcome will be inhaler technique. We will define inhaler technique in two ways:

a) Correct Use (i.e., >75% of steps correct)

b) Mastery (i.e., perfect technique, 100% steps correct)

This outcome will be measured at V1. We will test our hypothesis that patients will have improved inhaler technique post-VME compared to pre-VME. We will employ the same checklists used in the E-TRaIN study (IRB Protocol #11-0248, high reliability, kappa >0.94). These checklists will be used to measure the primary outcome: ability to use inhalers (defined as (a) correct use of inhaler: >9/12 steps for MDI; and (b) mastery of inhaler technique, i.e., perfect technique: 12/12 steps for MDI).

The **primary outcome** is the proportion of participants with MDI misuse post VME vs. pre VME. This will provide data on the short-term efficacy of VME.

Secondary outcomes

Secondary outcomes will include:

a) Symptom burden (i.e., BORG, ASSI, and CBSQ) at V1 and V2. Patient reported outcomes will be measured using a modified Borg dyspnea scale (Borg), Asthma Symptom Severity Index (ASSI), Chronic Bronchitis Symptom Questionnaire (CBSQ).
b) quality of life (QOL),[20]
c) and self-efficacy of inhaler technique.[8,10]

The efficacy of VME with regards to these secondary outcomes will employ simple univariate and/or multivariate logistic regression, generalized estimating equations, or generalized non-linear mixed models, as appropriate. For each of these outcomes, we will evaluate whether health literacy level modifies the effectiveness of VME by including an interaction term in the regression model. Model assumptions will be checked. A two-tailed p-value less than 0.05 will define statistical significance.

DATA COLLECTION SCHEDULE-AIM 1

	Inpatient Visit	
**VISIT**	**V0-V1**	
Visit duration (minutes)	60 (total for V0 to V1)	
Visit location	Inpatient	
Visit type	In-person	
Eligibility evaluation	■	
Consent	■	
Self Efficacy: Self-reported inhaler technique	■	
Patient demonstration of inhaler	■	■

technique		
Vision Screen		
Health Literacy	■	
VME Survey	■	
Spirometry before and after bronchodilator	■	
Review of medications	■	
Medication count/weigh		
Questionnaire about symptom burden, respiratory exacerbations, acute care utilization	■	
Discharge Questionnaire		■

Vo: initial assessment V1: after instruction V2: 30 days post-discharge follow-up

This study will require 2-6 months:

1. Pilot test interactive video round 1 (2 months)
2. Pilot test interactive video round 2 (2 months) if changes to VME needed
3. Pilot test interactive video round 3 (2 months) if changes to VME needed
4. Data analyses and preparation of final report (2 months).

The research will take place on the inpatient wards of UCM.

ANALYSES-AIM 1

Analysis plan. We will use means, medians, proportions, scatterplots, and histograms to describe the data. We will test for group differences using the *t*-test, the Wilcoxon rank sum test, Pearson's chi-squared test, or Fisher's exact tests as appropriate. We will adhere to the intention-to-treat principle when testing for differences by intervention type. The **primary outcome** will be correct inhaler technique; chi-squared tests will be used to compare post VME vs. pre VME prevalence of inhaler misuse (<75% of steps correct). **Secondary outcomes** will include self-efficacy for respiratory inhalers, symptom burden (i.e., BORG and CSS), baseline self-reported utilization of health care services for respiratory exacerbations, basic disease knowledge, and QOL. For each of these outcomes, we will evaluate whether patient characteristics (vision, age, and/or health literacy level) modifies the effectiveness of VME by evaluating interactions in the regression model. A two-tailed p-value less than 0.05 will define statistical significance.

SPECIFIC AIM 2

This will be a randomized, controlled, non-inferiority clinical trial conducted of patients admitted to the University of Chicago. Adults admitted with a history of Asthma or COPD will be eligible

to participate in this study. Upon receiving consent, participants will be randomized to either the VME or TTG. Inhaler technique will be measured prior to hospital discharge before and after education (VME or TTG). This will provide data on the short-term effectiveness of the interventions. Retention will be examined at 30 days after hospital discharge.

ELIGIBILITY CRITERIA

The goal of patient selection is to enroll adults admitted with a history of physician-diagnosed Asthma or COPD.

Inclusion criteria

1. Age 18 years and older
2. Admission to the inpatient medical service and surgical service
3. Physician-diagnosed Asthma or COPD. We will enroll patients even if the primary reason for admission is not Asthma or COPD (e.g., patients admitted for heart failure, but with a physician diagnosis of COPD are eligible).
4. Physician plans to discharge patients home a metered dose inhaler (MDI; e.g., albuterol)

Exclusion criteria

1. Currently in an intensive care unit
2. Physician declines to provide consent
3. Patient unable to provide consent (e.g., history of cognitive impairment, unable to understand English) or declines to provide consent
4. Previous participant in this study

RECRUITMENT

We will employ the same recruitment plan as in the protocol for our Effectiveness of interventions to Teach Respiratory Inhaler Technique (E-TRaIN) study (Protocol #11-0248). In summary, admission logs (EPIC) at the University of Chicago will be examined each day to screen for potentially eligible patients. The treating physician will be contacted for verbal assent using standardized text. Among patients whose physician provides assent, medical records will be reviewed to ascertain eligibility and informed consent will be sought from eligible patients. A total of 142 patients will be recruited at the University of Chicago Medical Center over 15 months.

TREATMENT CONDITIONS

1. **Teach-to-Goal (TTG):** During inpatient admission, participants assigned to the TTG conditon will be provided with an intensive, iterative education and evaluation strategy that consists of the following steps: First, participants will undergo an initial assessment of respiratory inhaler technique for MDI (and a Diskus® device if they are also prescribed medication using the device, i.e., fluticasone/salmeterol [Advair Diskus ®]) using standardized device-specific checklists (Form 1) that we have developed and used in the SARI study (Protocol #15729A), in TURI (#16900A), and in E-TRaIN (#11-0248). This will be followed by the educational intervention whereby participants will then be taught using verbal instructions and then by demonstration by a trained educator regarding the appropriate use of the MDI (and, if applicable, fluticasone/salmeterol (Advair Diskus®) using standardized written instructions (Forms 2a and 2b). Next the participants will be asked to demonstrate how they use their respiratory inhalers; their post-TTG technique will be graded using the same checklists as the initial assessment. The participants will receive additional rounds of instruction following this assessment if

needed (e.g., if the participant did not achieve mastery of the MDI or Diskus® after one round of teaching). Although initial pilot work showed that two rounds was sufficient, more recent data indicates that some participants require > 2 rounds; since the essence of this education technique is to teach-to-goal, and this requires only a few additional minutes (maxium) we will attempt to teach-to-goal. The patient's hospital physician (attending physician or their designee) will be provided information about the patients inhaler technique for both inhalers after education; this will provide an opportunity for the patient to receive addition instructions by the clinical team if needed. All instructions will be provided by a dedicated trained educator; all assessments will be performed by a second research assisstant (trained assessor). The trained assessor will be masked to treatment assignment (see below).[15]

2. **Video Module Education (VME):** The RE will provide participants with a tablet device and will demonstrate how to access VME and complete the pre/post e-learning assessments. The RE will provide technical support but will neither participate directly in the education nor help with the self-assessments. The participants will first complete the pre-assessment e-learning tool on the tablet. They will then watch the video instruction that will provide a complete demonstration with verbal instructions on correct inhaler technique. Next the participants will complete the post-assessment e-learning tool. Based on participants' performance, they will be directed to further tailored video-instruction. The cycle of self-assessment and video instruction will continue until sufficient mastery has been achieved.

<u>Other considerations:</u>

1. *Masking:* To minimize risk of biased measurements of inhaler technique, the trained assessor will be masked to the treatment condition. Specifically, the trained assessor will not be informed about the treatment assignment, will remain outside the patients' room during the educational intervention (and the room door will remain closed during the intervention), and the trained educator will be instructed not to inform the assessor about the treatment condition. We have successfully used similar procedures for masking the trained assessor in other IRB-approved studies (see E-TRaIN, IRB # 11-0248).

2. *Patients being discharged home on a MDI and a Diskus will receive VME or TTG interventions for both devices.* If randomized to the TTG intervention, then TTG will be used to teach MDI and Diskus use; likewise if randomized to the VME intervention, then VME will be used to teach MDI and Diskus use. Based on results of screening data from the TURI study (IRB #16900A), we expect that about 10-20% of participants (10 to 20 participants enrolled in this study) will be prescribed both devices. We would like to include this option to teach two different devices (in patients prescribed both devices) to collect preliminary data about the feasibility of VME for MDI vs. TTG for Diskus; these data would be valuable as we prepare for future studies.

PROTOCOL FOR BOTH GROUPS:

Following the screening procedures above, we will use the following steps:

11. **Obtain assent from treating physician prior to approaching patients hospitalized with asthma or COPD.** The treating physician will be contacted by a research assistant for verbal assent using standardized text (Form 3).

12. **Obtain written informed consent from patient using standardized text.** One hundred eighteen participants (N=59 TTG condition; N=59 VME condition) will be enrolled in this study.

13. **Optimize room conditions**
    - Room well lit (turn on overhead lights if necessary)
    - Patient preferably sitting up
    - Private setting (close door to patient room)
    - Interviewer should directly face patient
    - Patient should use reading glasses if they use one

14. **Assess patient's respiratory symptoms/morbidity (Forms 4-5-6), which includes the** Borg symptom score, Asthma Symptom Severity Index (ASSI), Chronic Bronchitis Symptom Questionnaire, COPD Severity Score (CSS), Airway Questionnaire (AQ-20), COPD Helplessness Index (CHI) and demographic and other clinical information.

15. **Assess patients' self-efficacy regarding respiratory inhaler use (Form 7)**

16. **Assess lung function. Perform spirometry pre and post-bronchodilator (after 2 puffs albuterol MDI via spacer) using portable KoKo spirometer. (Forms 8a and 8b).** These data will provide an objective measure of disease severity at the time of the hospitalization. We have previously demonstrated that this population has poor perception of airflow obstruction and spirometry is feasible during the inpatient setting.[16]

17. **Assess patient's inhaler technique using Inhaler checklists (Form 1;** for a MDI and, if applicable, Diskus®) by the trained assessor.

18. **Assess health literacy. Check vision (Form 9). Ask brief health literacy screen questions (Form 10a) for all participants and a**dminister STOHFLA questionnaire (Form 10b) if the patient's visual acuity is at least 20/50 in one eye (Check the patient's visual acuity in each eye using the Snellen Pocket Eye Chart (Form 11), otherwise, the patient is ineligible to complete the STOHFLA HL evaluation (will complete the BHLS questions).

    a) BHLS
    We want to find out what health care information is understood by patients and what is confusing to them, in order to help us understand what patients may find confusing.
    1) How often do you have problems learning about your medical condition because of difficulty understanding written information?
    2) How confident are you filling out medical forms by yourself?
    3) How often do you have someone help you read hospital material?

    b) STOHFLA
    Assess health literacy. Check vision (Form 9). Administer STOHFLA questionnaire (Form 10) if the patient's visual acuity is at least 20/50 in one eye (Check the patient's visual acuity in each eye using the Snellen Pocket Eye Chart (Form 11), otherwise, the

patient is ineligible to complete the HL evaluation. Explain the purpose of this survey. Use the following text.

- We want to find out what health care information is understood by patients and what is confusing to them, in order to help us write things more clearly.

- Here are some medical instructions that you or anybody might see around the hospital. These instructions are in sentences that have some of the words missing.

- Where a word is missing, a blank line is drawn, and 4 possible words that could go in the blank appear just below it. I want you to figure out which of those 4 words should go in the blank, which word makes the sentence make sense.

- When you think you know which one it is, circle the letter in front of that word, and go on to the next one.

- When you finish the page, turn the page and keep going until you finish all the pages.

- Provide the patient a pen and a clipboard and ask them to complete the questionnaire (Form 10). If the patients ask for help with completing the questionnaire, instruct them to do the best they can and that you are not allowed to assist them. Only responses completed within 7 minutes are used in calculating a health literacy score. Record the start and stop time and any questions that were answered after 7 minutes had elapsed. Do not grade the STOFHLA until after the study visit is complete.

19. **Obtain randomization assignment from the study biostatistician.** Randomization will be block-stratified by level of health literacy to ensure an equal number of subjects within each stratum assigned to TTG and VME Patients will then receive either the TTG or VME intervention by the trained educator.

20. **Collect patient contact information and provide patient $25 in cash for participating in this study as well as parking pass/bus pass for their follow-up visit.** Record patient's social security number and obtain patients signature that they received the patient incentive.

    1. **Provide patients an appointment for an in-person interview 30 days after discharge (patients will be given a letter with their appointment time).** At the in-person interview, we will ask patients to complete a short interviewer-administered questionnaire about patient's respiratory symptoms/morbidity/self-reported ability to use MDI and Diskus® inhalers, quality of life (Forms 4 and 12). We will also assess lung function (spirometry) and use of inhalers (MDI and/or Diskus). Upon completing this portion the participant will receive $50 as well as parking pass/bus pass for their next follow-up visit.

OUTCOMES/MEASURES-AIM 2

The primary and secondary outcomes are listed below. Measures for each are described. Initial measurements (Vo; see Data Collection Schedule below) will be made prior to

randomization; at V1, the trained educator will assess respiratory inhaler technique by asking the subject to demonstrate use of the MDI (and Diskus® if to be discharged home on fluticasone/salmeterol). Follow-up assessment, V2, will take place at 30 days (+/-7 days) post-hospital discharge.

Primary outcome

The primary outcome will be inhaler technique. We will define inhaler technique in two ways:

c) Correct Use (i.e., >75% of steps correct)
d) Mastery (i.e., perfect technique, 100% steps correct)

This outcome will be measured at V1 and V2. We will test our hypothesis that patients will have a reduced proportion of respiratory events when taught effective inhaler technique with TTG compared to BI education separately for the MDI and Diskus® devices after hospital discharge. We will employ the same checklists used in the E-TRaIN study (IRB Protocol #11-0248, high reliability, kappa >0.94). These checklists will be used to measure the primary outcome: ability to use inhalers (defined as (a) correct use of inhaler: >9/12 steps for MDI; >8/10 steps for Diskus®; and (b) mastery of inhaler technique, i.e., perfect technique: 12/12 steps for MDI; 10/10 steps for Diskus®).

The **primary outcome** is the proportion of participants with MDI misuse post VME vs. TTG education. This will provide data on the short-term effectiveness of the interventions. Longer-term retention (durability) will be examined 30 days post-hospital discharge using an intention-to-treat analysis. We have powered on this binary variable. [Table 1] However, because we recognize that there is more information about which steps are less critical and those that are more critical, I will also work with Robert Gibbons, a nationally recognized expert in biostatistics, to model a mixed effects ordinal regression model with random intercepts and time effects within correlation of each participant with terms for mode (VME and TTG), as well as interaction with time, to determine the predicted probability in each category for VME and TTG of how prevalence of misuse changes over time.[17] **Mediators include** health literacy[18] and vision level[19] as I have previously identified them as important mediators of baseline inhaler misuse and/or ability to learn correct technique.[8,10]

Secondary outcomes

Secondary outcomes will include:

d) Change in self-efficacy for respiratory inhalers (i.e., V0-V2). We will ask all patients to state how strongly they agree or disagree with the following statement "I am confident that I know how to use [insert inhaler name] correctly" for both MDI and Diskus®. The subject will be shown an example of each inhaler. Each item will be scored on a 5-point Likert scale (strongly disagree to strongly agree); the mean score will be used to measure self-efficacy. Cronbach's alpha will be calculated to evaluate internal consistency.
e) Symptom burden (i.e., BORG, ASSI, and CBSQ) at V1 and V2. Patient reported outcomes will be measured using a modified Borg dyspnea scale (Borg), Asthma Symptom Severity Index (ASSI), Chronic Bronchitis Symptom Questionnaire (CBSQ).
f) Acute care utilization for respiratory exacerbations at V2. Data will be collected using interviewer administrated surveys. *Respiratory events* are defined as: physician, emergency room or hospital visit, or death. This composite endpoint is due to small sample size.

The comparative effectiveness of TTG versus VME with regards to these secondary outcomes will employ simple univariate and/or multivariate logistic regression, generalized estimating equations, or generalized non-linear mixed models, as appropriate. For each of

these outcomes, we will evaluate whether health literacy level modifies the effectiveness of VME (Vs. TTG) by including an interaction term in the regression model. Model assumptions will be checked. Use of two inhalers will also provide the opportunity to evaluate if the comparative effectiveness of VME (vs. TTG) varies by device. A two-tailed p-value less than 0.05 will define statistical significance. The sample size calculations demonstrate that N=59 per group will provide 80% power for an intention-to-treat analysis to test for non-inferiority of VME vs. TTG.

Secondary Outcomes include quality of life (QOL),[20] patient reported outcomes,[20-21] lung function, utilization of health care services (outpatient visits, ED and hospital admissions, deaths), and self-efficacy of inhaler technique.[8,10] We will also evaluate the differential effects of VME vs. TTG by participant characteristics (e.g., age, health literacy). Finally we will evaluate the preliminary comparative durability of VME vs. TTG at 30 days post-hospital discharge. We will perform all tests separately for both MDI and Diskus devices.

Subjects in Aim 2 procedures will also be offered participation in the VME at Home sub-study. We will ask if subjects may be interested in participating in a secondary study to test the feasibility of using VME at home. The consent form will contain information with a separate line to initial if the subject would like to participate. If they initial this line, they will sign the separate VME at Home sub-study consent document. All subjects will be asked to use the VME on their own devices. They will be provided with an account to use the web-based VME, sent via email 3-5 days post-V2. Participants will be provided an appointment for an in-person interview 30 days after V2. A reminder phone call will be made once a week to ensure VME has been accessed. At 30-days post-V2, an in-person interview or phone interview will be completed to assess the following:

1. Assess patient's inhaler technique using Inhaler checklists (From 1; for a MDI and if applicable, Diskus®) by the trained assessor.

2. Acute care utilization for respiratory exacerbations. Data will be collected using interviewer administered surveys. *Respiratory events* are defined as: physician, emergency room or hospital visit, or death. This composite endpoint is due to small sample size.

3. VME at home Questionnaire

- Did you use the VME?
  - Yes
  - No
- How many times did you use the VME?
  - 0
  - 1-2
  - 3-5
  - Weekly
  - Daily
  - more than once per day
- If you did not use the VME, what were some reasons why?
- If you did use the VME:
  - Did the VME help you feel more confident about using your inhaler at home?
  - How long did it take you to use the VME at home?

- Did you require help from anyone else in order to use the VME? Who helped you?
- Is there any difference between the instructions in the video and those given by your doctor?
  - Yes
  - No
- What device did you use to access the VME?
  - Computer
  - Tablet
  - Smartphone

DATA COLLECTION SCHEDULE-AIM 2

VISIT	Inpatient Visit V0-V1		30 day FU V2
Visit duration (minutes)	60 (total for V0 to V1)		60
Visit location	Inpatient		Outpatient
Visit type	In-person		In-person
Eligibility evaluation	■		
Consent	■		
Randomization	■		
Self Efficacy: Self-reported inhaler technique	■		■
Patient demonstration of inhaler technique	■	■	■
Vision Screen	■		
Health Literacy	■		
Spirometry before and after bronchodilator	■		■
Review of medications	■		■
Medication count/weigh			■
Questionnaire about symptom burden, respiratory exacerbations, acute care utilization	■		■
Discharge Questionnaire		■	

Vo: initial assessment V1: after instruction V2: 30 days post-discharge follow-up

This study will require 24 months:

5. Develop/pilot test interactive video (6 month)
6. Training of personnel (1 month)
7. Begin recruitment (1-3 participants/week, for 15 months)
8. Complete follow-up of last enrollee (1 month after last enrolled participant)
9. Data analyses and preparation of final report (2 months).

The research will take place on the inpatient wards of UCM.

ANALYSES-AIM 2

Analysis plan. We will use means, medians, proportions, scatterplots, and histograms to describe the data. We will test for group differences using the *t*-test, the Wilcoxon rank sum test, Pearson's chi-squared test, or Fisher's exact tests as appropriate. We will adhere to the intention-to-treat principle when testing for differences by intervention type. The **primary outcome** will be correct inhaler technique; chi-squared tests will be used to compare post VME vs. post TTG prevalence of inhaler misuse (<75% of steps correct). **Secondary outcomes** will include change in self-efficacy for respiratory inhalers, symptom burden (i.e., BORG and CSS), self-reported utilization of health care services for respiratory exacerbations, basic disease knowledge, and QOL. The comparative effectiveness of VME versus TTG with regards to these secondary outcomes will employ simple univariate and/or multivariate logistic regression, as appropriate. For each of these outcomes, we will evaluate whether patient characteristics (vision, age, and/or health literacy level) modifies the effectiveness of VME versus TTG by evaluating interactions in the regression model. A two-tailed p-value less than 0.05 will define statistical significance. The sample size calculations (see below) demonstrate that n=59 per group will provide 80% power for an intention-to-treat analysis.

Sample size. (Table 1) The main outcome is correct MDI use (>75% steps correct). In my preliminary studies above, the proportion of participants with correct MDI use after TTG was 88-100% (n=98, n=50, respectively).[8,10] These data (expected proportion of correct MDI use of 0.95), along with a noninferiority effect size of -0.10, were used to calculate the sample size.[22] We will enroll 142 participants over 15 months and will have sufficient power (0.8) to determine if VME is non-inferior to TTG, at a significance level of p<0.05. We have experience recruiting participants and have been able to recruit up to 12 participants per month; therefore, we do not foresee any problems with recruiting this number of participants in this timeline.

Table 1: Sample Size per intervention (VME/TTG)

	**Power**				
**EFFECT SIZE**	**0.60**	**0.70**	**0.80**	**0.90**	**0.95**
**-0.05**	137	179	236	326	412
**-0.10**	35	45	59	82	103
**-0.15**	16	20	27	37	46

<u>Interim analyses:</u> After 50% of the target sample size have completed the study (59 participants have completed the study) or after 50% of the study period (~7 months), whichever occurs first, results of an interim analysis will be provided to a Data Safety and Monitoring Board (Drs. Dana Edelson, MD; Steve White, MD; Neda Laiteerapong, MD; physicians who are not study investigators). Investigators and research assistants will remain masked to the results of the interim analyses until the end of the study. The DSMB will be asked to consider issues related to study performance (enrollment rates, study completion rates) and differences in outcomes between groups and provide recommendations to the PI and the IRB whether to continue the study or terminate enrollment.

<u>BENEFITS AND RISKS AIMS1 AND 2</u>

Benefits

We are not aware of any potential benefits to study subjects.

Risks

Potential risks include:

1. The subject may be uncomfortable answering some interview questions. They can refuse to answer any question.

2. The lung function test is safe and is commonly used to measure the severity of COPD but can cause some minor chest soreness or lightheadedness; study test will be done by a trained member of the research staff with the subject in a seated position. To further minimize the risk of adverse events with spirometry, we will not perform spirometry in subjects with SBP>180 or DBP>100 mm Hg.

3. Two puffs of albuterol (for post-bronchodilator spirometry) is generally well tolerated. We have not had adverse events with 2 puffs albuterol after enrolling >170 subjects in the SARI, COAT, and TURI studies (IRBs# 15729A, 14831A, 16900A). In some cases, however, 2 puffs of albuterol can cause jitteriness, anxiety, and tachyarrhythmias. To minimize this risk, we will withhold albuterol in subjects who are hospitalized with tachyarrhythmias or palpitations or report problems after using albuterol.

4. Long acting beta-2-agonists (salmeterol or formoterol). These medications are generally well-tolerated, but can have albuterol-like adverse effects; in addition, these agents have been associated with increase risk of asthma-related deaths.

5. Inhaled corticosteroids (fluticasone/budesonide). These medications are well tolerated, but can cause thrush, dysphonia, and other less common adverse effects.

6. Loss of confidentiality. To help ensure that patients' health information remains private, we will restrict access to data collected for our study to study personnel only (via use of password-protection and locked cabinets for study documents). Study ID numbers will be generated and will be used when discussing and/or reviewing data at study meetings. Also, study reports will report results in aggregate and not contain information that can be used to identify individual patients.

Data Safety Monitoring Plan-AIM 2

The PI will review monthly data reports reviewing recruitment, consent, intervention activities, adverse events, and follow-up. Although few, if any, intervention-related adverse events are anticipated in this education-related study, the study population is at risk for poor outcomes, including life-threatening exacerbations and death. The PI will comply with prompt reporting of any unanticipated events and the Data Safety and Monitoring Board (DSMB) (Dana Edelson, MD, Steve White, MD, Neda Laiteerapong, MD). The DSMB (see interim analysis above) will also review results of the interim and final analyses.

PATIENT INCENTIVES-AIM 1 AND 2

Participants will be provided $25 for completing the initial baseline interview (only interview for AIM 1, first interview for AIM 2) and $50 completing the post-hospital discharge in-person interview. Total possible monetary compensation is $25 for AIM 1 and $75 for AIM 2.

Participants in the VME at home sub-study will be provided $50 for completing the 30-day in-person interview or $10 for completing the interview via phone. For AIM 2, participants will also be provided parking passes or bus passes for their follow-up visits as we recognize that this cost burden should not fall on the participant for participating in our study. Monetary compensation will be reduced, based on the extent to which the participante completes the study. For both AIMs all tests for this study will be done free of charge.

BIBLIOGRAPHY

1. Morbidity and Mortality 2007 Chart Book on Cardiovascular, Lung, and Blood diseases. Available at: http://www.nhlbi.nih.gov/resources/docs/07-chtbk.pdf. Accessed June 06, 2012.

2. Jencks SF, Williams MV, Coleman EA. Rehospitalizations among patients in the Medicare fee-for-service program. N Engl J Med 2009;360(14):1418-1428. PMID: 19339721.

3. Hanania NA, Darken P, Horstman D, Reisner C, Lee B, Davis S, Shah T. The efficacy and safety of Fluticasone Propionate (250ug) /Salmeterol (50ug) combined in the Diskus® Inhaler for the treatment of COPD. Chest 2003;124:834-843. PMID: 12970006.

4. Celli BR, Thomas NE, Anderson JA, Ferguson GT, Jenkins CR, Jones PW, Vestbo J, Knobil K, Yates JC, Calverley PMA. Effect of pharmacotherapy on rate of decline of lung function in chronic obstructive pulmonary disease. Results from the TORCH Study. Am J Resp Crit Care Med 2008;178:332-338. PMID: 18511702.

5. Melani, AS, Bonavia M, Cilenti V, Cinti C, Lodi M, Martucci P, Serra M, Scichilone N, Sestini P, Aliani M, Neri M, on behalf of the Gruppo Educazionale Associazione Italiana Penumologi Ospedalieri (AIPO). Inhaler mishandling remains common in real life and is associated with reduced disease control. Respir Med 2011;105:930-938. PMID: 21367593.

6. Dolovich MB, Ahrens RC, Hess DR, Anderson P, Dhand R, Rau JL, Smaldone GC, Guyatt G. Device selection and outcomes of aerosol therapy: Evidence-based guidelines: American College of Chest Physicians/American College of Asthma, Allergy, and Immunology Chest. 2005;127:335-71. PMID: 21367593.

7. The Global initiative for Chronic Obstructive Lung Disease (GOLD) Guidelines: Available at: http://www.goldcopd.com/. Accessed June 6, 2012.

7.5 Guidelines for the Diagnosis and Management of Asthma (EPR-3). Available at: https://www.nhlbi.nih.gov/guidelines/asthma/. Accessed June 6, 2012.

8. Press VG, Shah LM, Lewis S, Ivy K, Charbeneau J, Mazurek A, Badlani S, Naureckas E, Krishnan JA. Misuse of respiratory inhalers in hospitalized patients with asthma or COPD. J Gen Intern Med 2012;26(6), 635-642. PMID: 21249463 PMCID: PMC3101982.

9. Cohen DJ, Clark EC, Lawson PJ, Casucci BA, Flocke SA. Identifying teachable moments for health behavior counseling in primary care. Patient Educ Couns 2011;85:e8-e15. PMID: 21183305

10. Press VG, Arora VM, Lewis SL, Charbeneau J, Starkey J, Naureckas ET, Krishnan JA. Teaching the use of respiratory inhalers to hospitalized patients with asthma or COPD: A randomized trial (TURI). J Gen Intern Med May 2012. [Epub ahead of print.] PMID: 22592354.

11. Paasche-Orlow MK, Riekert KA, Bilderback A, Chanmugam A, Hill P, Rand CS. Tailored education may reduce health literacy disparities in asthma self-management. Am J Respir Crit Care Med 2005;172(8):980-986. PMID: 16081544 PMCID: PMC2718412.

12. Virchow JC, Crompton GK, Negro RD, Pedesen S, Magnan A, Seidenberg J, Barnes PJ. Importance of inhaler devices in the management of airway disease. Respir Med 2008;102:10-19. PMID: 17923402.

13. Jack BW, Chetty VK, Anthony D, Greenwalk JL, Sanchez GM, Johnson AE, Forsythe SR, O'Donnell JK, Paasche-Orlow MK, Manasseh C, Martin S, Culpepper L. A Reengineered Hospital Discharge Program to Decrease Rehospitalization. A Randomized Trial. Ann Intern Med 2009;150(3):178:187. PMID: 19189907. PMCID: PMC2738592.

14. Gerber BS, Brodsky IG, Lawless KA, Smolin LI, Arozullah AM, Smith EV, Berbaum ML, Heckerling PS, Eiser AR. Implementation and evaluation of a low-literacy diabetes education computer multimedia application. Diabetes Care 2005;28(7):1574-1580. PMID: 15983303.

15. Press VG, Shah LM, Lewis SL, Ivy K, Charbeneau JT, Mazurek A, Krishnan JA. Teach-to-goal intervention to teach respiratory inhaler technique in patients hospitalized for asthma or COPD. Abstract, accepted for poster presentation, Society of General Internal Medicine Annual Meeting May 2009.

16. Davis SQ, Permutt Z, Permutt S, Naureckas ET, Bilderback AL, Rand CS, Stein BD, Krishnan JA. Perception of airflow obstruction in patients hospitalized for acute asthma. *Annals of Asthma, Allergy, and Immunology.* 2009*;102(6)455-61.*

17. Hedeker D, Gibbons RD. Longitudinal Data Analysis. New Jersey: John Wiley & Sons; 2006.

18. Nurss JR, Parker RM, Williams MV, Baker, DW. Short Test of Functional Health Literacy in Adults (STOFHLA). Hartford: Peppercorn Books & Press; 2001.

19. Snellen Eye Chart: A Description and Explanation. Available at: http://precision-vision.com/index.cfm/feature/24_149/snellen-eye-chart---a-description-and-explanation.cfm. Accessed June 6, 2012.

20. Kendrick KR, Baxi SC, Smith RM. Usefulness of the modified 0-10 Borg scale in assessing the degree of dyspnea in patients with COPD and asthma. J Emerg Nurs 2002;26:216-222. PMID: 10839848.

21. Eisner MD, Trupin L, Katz PP, Yelin EH, Earnest G, Balmes J, Blanc PD. Development and validation of a survey-based COPD severity score. Chest 2005;127:1890-1897. PMID: 15947299.

22. Hung HMJ, Wang SJ, O'Neill R. A regulatory perspective on choice of margin and statistical inference issue in non-inferiority trials. Biom J 2005;47(1):28-36. PMID: 16395994.

Version: 15May17

Protocol Title: Video vs. TTG Respiratory inhaler technique Assessment and InstructioN (V-TRaIN)

University of Chicago
PI: Valerie G. Press, MD, MPH

Co-Investigators: David Meltzer, MD, PhD, Vineet Arora, MD, MAPP, Ted Naureckas, MD

Research personnel: John Kim, Nicole Woodrick, Nicole Twu, Ashley Hull, Ashley Snyder

BACKGROUND

Asthma and Chronic obstructive pulmonary disease (COPD) results in over a million hospitalizations in the United States annually[1] and COPD is the third leading cause of 30-day re-hospitalizations.[2] Clinical trials have established the efficacy of treatments primarily dispensed via respiratory inhaler devices that reduce morbidity and health care utilization if they are used correctly.[3-4] Unfortunately, the effectiveness of these medications in real-world settings is limited by the fact that patients often do not use inhalers correctly.[5-6] Current guidelines recommend assessing and teaching inhaler technique at all health care encounters, including hospitalization.[77.5] My work has found that over 75% of hospitalized patients in an urban, predominantly underserved population misuse their respiratory inhalers, highlighting a missed opportunity to educate these patients with high potential to benefit.[8] Hospitalization, therefore, provides a potential ‘teachable moment’ to correct this misuse.[9] My preliminary data indicate that one strategy, in-person teach-to-goal (TTG), is effective in teaching hospitalized patients proper inhaler technique and is more effective than simple verbal instruction.[10]

While TTG is a promising method to improve care for patients who use inhalers, several limitations prevent widespread adoption. First, TTG relies on in-person assessment and education, as well as training and monitoring instructors to ensure fidelity, making it time-consuming and costly. Also, because a single educational session does not ensure long-term retention,[11-12] post-discharge reinforcement may be needed, which may be impractical with in-person TTG. One potential method to surmount TTG's limitations is use of interactive video module education (VME), a method that has been used for health education in other clinical contexts.[13-14] Through iterative self-assessments and video-demonstrations on a tablet computer, VME has the potential to be less costly, maintain fidelity, and be more easily extended into the post-discharge setting than in-person TTG. However, certain questions remain about VME. It is unclear whether VME will yield similar results when compared to TTG, or whether urban, underserved patients will have the ability to, and be willing to use, VME in the post-discharge setting. Therefore, before widespread implementation of VME, it is critical to rigorously develop and test VME for inhaler education in the hospital setting. Ultimately, it will also be important to understand patients’ ability and willingness to use post-discharge VME for educational reinforcement to allow for this strategy to transition patients across care settings from hospital to home. Through these funded projects, I will acquire critical data I need to develop R-01 studies focused on improving self-management for patients with obstructive pulmonary disease across care transitions.

<u>We hypothesize that interactive VME will lead to non-inferior rates of ability to demonstrate correct inhaler use compared to rates with TTG among hospitalized patients with Asthma or COPD.</u>

The specific aims to test this hypothesis are:

1. **To develop and iteratively test VME to teach correct use of metered dose inhaler (MDIs) and dry-powder Diskus® devices to hospitalized patients with Asthma or COPD.**

   We partnered with Click to Play Media™ (C2P) and Smart Sparrow to develop VME modules that (self)-assess and teach respiratory inhaler technique to hospitalized participants. We have tested the VME in focus groups (IRB #13-1139). Now that the VME is developed we will iteratively test (n=30-40) the VME strategy (up to 3 rounds (90-120 total)) to obtain important patient feedback, preferences and preliminary efficacy estimates for the module.

   We hypothesize that participants' post-VME inhaler technique will be significantly better than their pre-VME inhaler technique.

2. **To determine if the proportion of hospitalized patients with Asthma or COPD who demonstrate correct inhaler technique after receiving VME is not significantly less than (non-inferior to) the proportion who demonstrate correct inhaler technique after receiving in-person TTG.**

   To accomplish this aim we will build on our prior work with developing and evaluating the in-person TTG strategy to develop a parallel VME strategy that incorporates guideline-recommended components of assessment and instruction. We will conduct a behavioral randomized clinical trial to evaluate the relative effectiveness and durability of VME versus in-person TTG education on the ability of hospitalized patients to demonstrate correct MDI and Diskus® use. Secondarily, we will assess whether there is variation in comparative effectiveness by patient characteristics including age, gender, and health literacy level, among other important factors.

We hypothesize that interactive VME will lead to non-inferior rates of ability to demonstrate correct inhaler use compared to rates with TTG among hospitalized patients with Asthma or COPD.

SPECIFIC AIM 1

This will be a pre/post study of inpatients admitted to the University of Chicago. Adults admitted with a history of physician diagnosed COPD or asthma will be eligible to participate in this study. Upon receiving consent, participants will enrolled. Inhaler technique will be measured pre and post VME. This will provide data on the short-term efficacy of the VME.

Inclusion criteria:

1. Age 18 years and older
2. Admission to the inpatient medical service and surgical service
3. Physician-diagnosed COPD or asthma. We will enroll patients even if the primary reason for admission is not COPD or asthma (e.g., patients admitted for heart failure, but with a physician diagnosis of COPD are eligible).
4. Physician plans to discharge patients home a metered dose inhaler (MDI; e.g., albuterol)

Exclusion criteria

1. Currently in an intensive care unit
2. Physician declines to provide consent
3. Patient unable to provide consent (e.g., history of cognitive impairment, unable to understand English) or declines to provide consent
4. Less than 20/50 vision in both eyes

RECRUITMENT

Admission logs (EPIC) at the University of Chicago will be examined each day to screen for potentially eligible patients. The treating physician will be contacted for verbal assent using standardized text. Among patients whose physician provides assent, medical records will be reviewed to ascertain eligibility and informed consent will be sought from eligible patients. Sixty to ninety participants (approximately 30 per iterative round of VME up to 3 rounds) will be recruited at the University of Chicago Medicine over 2-6 months.

AIM 1 and AIM 2 Enrollment Numbers:

AIM 1 will enroll between 90-120 subjects and AIM 2 will enroll 142 for a combined enrollment of up to 262.

Video Module Education (VME):

The RE will provide participants with a tablet device and will demonstrate how to access VME and complete the pre/post e-learning assessments. The RE will provide technical support but will neither participate directly in the education nor help with the self-assessments. The participants will first complete the pre-assessment e-learning tool on the tablet. They will then watch the video instruction that will provide a complete demonstration with verbal instructions on correct inhaler technique. Next the participants will complete the post-assessment e-learning tool. Based on participants' performance, they will be directed to further tailored video-instruction. The cycle of self-assessment and video instruction will continue until sufficient mastery has been achieved.

Following the screening procedures above, we will use the following steps:

1. **Obtain assent from treating physician prior to approaching patients hospitalized with asthma or COPD.** The treating physician will be contacted by a research assistant for verbal assent using standardized text (Form 3).

2. **Obtain written informed consent from patient using standardized text.**

3. **Optimize room conditions**
   - Room well lit (turn on overhead lights if necessary)
   - Patient preferably sitting up
   - Private setting (close door to patient room)
   - Interviewer should directly face patient
   - Patient should use reading glasses if they use one

4. **Assess patient's respiratory symptoms/morbidity (<u>Forms 4-5-6</u>), which includes the** Borg symptom score, Asthma Symptom Severity Index (ASSI), Chronic Bronchitis

Symptom Questionnaire, COPD Severity Score (CSS), Airway Questionnaire (AQ-20), COPD Helplessness Index (CHI) and demographic and other clinical information.

5. **Assess patients' self-efficacy regarding respiratory inhaler use (Form 7)**

6. **Assess lung function. Perform spirometry pre and post-bronchodilator (after 2 puffs albuterol MDI via spacer) using portable KoKo spirometer. (Forms 8a and 8b).** These data will provide an objective measure of disease severity at the time of the hospitalization. We have previously demonstrated that this population has poor perception of airflow obstruction and spirometry is feasible during the inpatient setting.[16]

7. **Assess patient's inhaler technique using Inhaler checklists (Form 1;** for a MDI) by the trained assessor.

8. **Assess health literacy. Check vision (Form 9). Ask brief health literacy screen questions (Form 10a) for all participants and a**dminister STOHFLA questionnaire (Form 10b) if the patient's visual acuity is at least 20/50 in one eye (Check the patient's visual acuity in each eye using the Snellen Pocket Eye Chart (Form 11). If the patient's visual acuity is not 20/50 in at least one eye, administer readers.

   - 4 strengths of glasses: 2.00, 2.25, 2.75, and 3.25
   - Start with 2.00, and repeat the vision screen, with each eye separately
   - If visual acuity of 20/50 is reached in at least one eye, the patient is eligible to complete the STOHFLA HL evaluation using the readers
   - If vision is not corrected after trying all of the strengths of the readers, the patient is ineligible to complete the STOHFLA HL evaluation

a) BHLS

We want to find out what health care information is understood by patients and what is confusing to them, in order to help us understand what patients may find confusing.]

1) How often do you have problems learning about your medical condition because of difficulty understanding written information?
2) How confident are you filling out medical forms by yourself?
3) How often do you have someone help you read hospital material?

b) STOHFLA

Explain the purpose of this survey. Use the following text.

- We want to find out what health care information is understood by patients and what is confusing to them, in order to help us write things more clearly.

- Here are some medical instructions that <u>you or anybody might see</u> around the hospital. These instructions are in sentences that have some of the words missing.

- Where a word is missing, a blank line is drawn, and 4 possible words that could go in the blank appear just below it. I want you to figure out which of those 4 words should go in the blank, which word makes the sentence make sense.

- When you think you know which one it is, circle the letter in front of that word, and go on to the next one.
- When you finish the page, turn the page and keep going until you finish all the pages.
- Provide the patient a pen and a clipboard and ask them to complete the questionnaire (Form 10). If the patients ask for help with completing the questionnaire, instruct them to do the best they can and that you are not allowed to assist them. Only responses completed within 7 minutes are used in calculating a health literacy score. Record the start and stop time and any questions that were answered after 7 minutes had elapsed. Do not grade the STOFHLA until after the study visit is complete.

9. Administer VME Survey

Explain the purpose of this survey. Use the following text.

We are thinking of doing a study that would use interactive video modules to teach patients about skills to manage their health problems. Video modules include answering a few questions, followed by watching a video to teach about a health problem or skill, followed by a few questions to see if the video was helpful.

- D1.a. How likely would you be to use an **interactive** video-module education **program** after discharge to learn about skills to help your health problem?
- D1.b. How much do you agree with this statement: "I would be willing to use the video-module education but do not have access (do not have devices and/or internet) needed to use this education" (circle one)
- D1.c. How much to you agree with this statement: "I would be willing to use the video-module education but I do not think I would know how (be able to) even though I have a device (laptop/smartphone/tablet).
- D1.d1. How much do you agree with this statement: " I have NO interest in using the video-module education after being discharged from the hospital."
    i. d2. If not, please explain:
- D1.e. If you think you might use the video-module education, what device would you use to participate? (check all that apply)
- D1.f. How much do you agree with this statement: "I would be willing to use video-module education after discharge home if I was provided with **access** to (a computer, tablet, cell phone with internet (smartphone), etc))"

- D1.g. g. How much to you agree with this statement: "I would be willing to use a public resource such as a library, drugstore, grocery store, church or temple to be able to use the video-module education after being discharged from the hospital."

10. **Collect patient contact information and provide patient $25 in cash for participating in this study.** Record patient's social security number and obtain patients signature that they received the patient incentive.

As part of Aim 1 procedures, we will ask if subjects may be interested in participating in a secondary study to test the feasibility of using VME at home. The consent form will contain information with a separate line to initial if the subject would like to participate.

5-10 subjects will be included in the home feasibility study. All subjects will be asked to use the VME on their own devices. They will be provided with an account to use the web-based VME, sent via email 3-5 days post-discharge. There will be an email reminder sent 7 days later if the VME has not yet been accessed. At 30-days post-discharge, the subject will receive a follow up phone call. This phone call will assess the following:

- Did you use the VME?
  - Yes
  - No
- How many times did you use the VME?
  - 0
  - 1-2
  - 3-5
  - Weekly
  - Daily
  - more than once per day
- If you did not use the VME, what were some reasons why?
- If you did use the VME:
  - Did the VME help you feel more confident about using your inhaler at home?
  - How long did it take you to use the VME at home?
  - Did you require help from anyone else in order to use the VME? Who helped you?
  - Is there any difference between the instructions in the video and those given by your doctor?
    - Yes
    - No
  - What device did you use to access the VME?
    - Computer
    - Tablet
    - Smartphone

OUTCOMES/MEASURES-AIM 1

The primary and secondary outcomes are listed below. Measures for each are described. Initial measurements (Vo; see Data Collection Schedule below) will be made prior to randomization; at V1, the trained educator will assess respiratory inhaler technique by asking the subject to demonstrate use of the MDI.

Primary outcome

The primary outcome will be inhaler technique. We will define inhaler technique in two ways:

a) Correct Use (i.e., >75% of steps correct)
b) Mastery (i.e., perfect technique, 100% steps correct)

This outcome will be measured at V1. We will test our hypothesis that patients will have improved inhaler technique post-VME compared to pre-VME. We will employ the same checklists used in the E-TRaIN study (IRB Protocol #11-0248, high reliability, kappa >0.94). These checklists will be used to measure the primary outcome: ability to use inhalers (defined as (a) correct use of inhaler: >9/12 steps for MDI; and (b) mastery of inhaler technique, i.e., perfect technique: 12/12 steps for MDI).

The **primary outcome** is the proportion of participants with MDI misuse post VME vs. pre VME. This will provide data on the short-term efficacy of VME.

Secondary outcomes

Secondary outcomes will include:

a) Symptom burden (i.e., BORG, ASSI, and CBSQ) at V1 and V2. Patient reported outcomes will be measured using a modified Borg dyspnea scale (Borg), Asthma Symptom Severity Index (ASSI), Chronic Bronchitis Symptom Questionnaire (CBSQ).
b) quality of life (QOL),[20]
c) and self-efficacy of inhaler technique.[8,10]

The efficacy of VME with regards to these secondary outcomes will employ simple univariate and/or multivariate logistic regression, generalized estimating equations, or generalized non-linear mixed models, as appropriate. For each of these outcomes, we will evaluate whether health literacy level modifies the effectiveness of VME by including an interaction term in the regression model. Model assumptions will be checked. A two-tailed p-value less than 0.05 will define statistical significance.

DATA COLLECTION SCHEDULE-AIM 1

	Inpatient Visit
**VISIT**	**V0-V1**
Visit duration (minutes)	60 (total for V0 to V1)
Visit location	Inpatient
Visit type	In-person

Eligibility evaluation	■	
Consent	■	
Self Efficacy: Self-reported inhaler technique	■	
Patient demonstration of inhaler technique	■	■
Vision Screen		
Health Literacy	■	
VME Survey	■	
Spirometry before and after bronchodilator	■	
Review of medications	■	
Medication count/weigh		
Questionnaire about symptom burden, respiratory exacerbations, acute care utilization	■	
Discharge Questionnaire		■

Vo: initial assessment V1: after instruction V2: 30 days post-discharge follow-up

This study will require 2-6 months:

1. Pilot test interactive video round 1 (2 months)
2. Pilot test interactive video round 2 (2 months) if changes to VME needed
3. Pilot test interactive video round 3 (2 months) if changes to VME needed
4. Data analyses and preparation of final report (2 months).

The research will take place on the inpatient wards of UCM.

ANALYSES-AIM 1

Analysis plan. We will use means, medians, proportions, scatterplots, and histograms to describe the data. We will test for group differences using the *t*-test, the Wilcoxon rank sum test, Pearson's chi-squared test, or Fisher's exact tests as appropriate. We will adhere to the intention-to-treat principle when testing for differences by intervention type. The **primary outcome** will be correct inhaler technique; chi-squared tests will be used to compare post VME vs. pre VME prevalence of inhaler misuse (<75% of steps correct). **Secondary outcomes** will include self-efficacy for respiratory inhalers, symptom burden (i.e., BORG and CSS), baseline self-reported utilization of health care services for respiratory exacerbations, basic disease knowledge, and QOL. For each of these outcomes, we will evaluate whether patient characteristics (vision, age, and/or health literacy level) modifies the effectiveness of VME by evaluating interactions in the regression model. A two-tailed p-value less than 0.05 will define statistical significance.

SPECIFIC AIM 2

This will be a randomized, controlled, non-inferiority clinical trial conducted of patients admitted to the University of Chicago. Adults admitted with a history of Asthma or COPD will be eligible to participate in this study. Upon receiving consent, participants will be randomized to either the VME or TTG. Inhaler technique will be measured prior to hospital discharge before and after education (VME or TTG). This will provide data on the short-term effectiveness of the interventions. Retention will be examined at 30 days after hospital discharge.

ELIGIBILITY CRITERIA

The goal of patient selection is to enroll adults admitted with a history of physician-diagnosed Asthma or COPD.

Inclusion criteria
1. Age 18 years and older
2. Admission to the inpatient medical service and surgical service
3. Physician-diagnosed Asthma or COPD. We will enroll patients even if the primary reason for admission is not Asthma or COPD (e.g., patients admitted for heart failure, but with a physician diagnosis of COPD are eligible).
4. Physician plans to discharge patients home a metered dose inhaler (MDI; e.g., albuterol)

Exclusion criteria
1. Currently in an intensive care unit
2. Physician declines to provide consent
3. Patient unable to provide consent (e.g., history of cognitive impairment, unable to understand English) or declines to provide consent
4. Previous participant in this study

RECRUITMENT
We will employ the same recruitment plan as in the protocol for our Effectiveness of interventions to Teach Respiratory Inhaler Technique (E-TRaIN) study (Protocol #11-0248). In summary, admission logs (EPIC) at the University of Chicago will be examined each day to screen for potentially eligible patients. The treating physician will be contacted for verbal assent using standardized text. Among patients whose physician provides assent, medical records will be reviewed to ascertain eligibility and informed consent will be sought from eligible patients. A total of 142 patients will be recruited at the University of Chicago Medical Center over 15 months.

TREATMENT CONDITIONS

1. **Teach-to-Goal (TTG):** During inpatient admission, participants assigned to the TTG conditon will be provided with an intensive, iterative education and evaluation strategy that consists of the following steps: First, participants will undergo an initial assessment of respiratory inhaler technique for MDI (and a Diskus® device if they are also prescribed medication using the device, i.e., fluticasone/salmeterol [Advair Diskus ®]) using standardized device-specific checklists (Form 1) that we have developed and used in the SARI study (Protocol #15729A), in TURI (#16900A), and in E-TRaIN (#11-0248). This will be followed by the educational intervention whereby participants will then be

taught using verbal instructions and then by demonstration by a trained educator regarding the appropriate use of the MDI (and, if applicable, fluticasone/salmeterol (Advair Diskus®) using standardized written instructions (Forms 2a and 2b). Next the participants will be asked to demonstrate how they use their respiratory inhalers; their post-TTG technique will be graded using the same checklists as the initial assessment. The participants will receive additional rounds of instruction following this assessment if needed (e.g., if the participant did not achieve mastery of the MDI or Diskus® after one round of teaching). Although initial pilot work showed that two rounds was sufficient, more recent data indicates that some participants require > 2 rounds; since the essence of this education technique is to teach-to-goal, and this requires only a few additional minutes (maxium) we will attempt to teach-to-goal. The patient's hospital physician (attending physician or their designee) will be provided information about the patients inhaler technique for both inhalers after education; this will provide an opportunity for the patient to receive addition instructions by the clinical team if needed. All instructions will be provided by a dedicated trained educator; all assessments will be performed by a second research assisstant (trained assessor). The trained assessor will be masked to treatment assignment (see below).[15]

2. **Video Module Education (VME):** The RE will provide participants with a tablet device and will demonstrate how to access VME and complete the pre/post e-learning assessments. The RE will provide technical support but will neither participate directly in the education nor help with the self-assessments. The participants will first complete the pre-assessment e-learning tool on the tablet. They will then watch the video instruction that will provide a complete demonstration with verbal instructions on correct inhaler technique. Next the participants will complete the post-assessment e-learning tool. Based on participants' performance, they will be directed to further tailored video-instruction. The cycle of self-assessment and video instruction will continue until sufficient mastery has been achieved.

<u>Other considerations:</u>

1. *Masking:* To minimize risk of biased measurements of inhaler technique, the trained assessor will be masked to the treatment condition. Specifically, the trained assessor will not be informed about the treatment assignment, will remain outside the patients' room during the educational intervention (and the room door will remain closed during the intervention), and the trained educator will be instructed not to inform the assessor about the treatment condition. We have successfully used similar procedures for masking the trained assessor in other IRB-approved studies (see E-TRaIN, IRB # 11-0248).

2. *Patients being discharged home on a MDI and a Diskus will receive VME or TTG interventions for both devices.* If randomized to the TTG intervention, then TTG will be used to teach MDI and Diskus use; likewise if randomized to the VME intervention, then VME will be used to teach MDI and Diskus use. Based on results of screening data from the TURI study (IRB #16900A), we expect that about 10-20% of participants (10 to 20 participants enrolled in this study) will be prescribed both devices. We would like to include this option to teach two different devices (in patients prescribed both devices) to collect preliminary data about the feasibility of VME for MDI vs. TTG for Diskus; these data would be valuable as we prepare for future studies.

PROTOCOL FOR BOTH GROUPS:

Following the screening procedures above, we will use the following steps:

11. **Obtain assent from treating physician prior to approaching patients hospitalized with asthma or COPD.** The treating physician will be contacted by a research assistant for verbal assent using standardized text (Form 3).

12. **Obtain written informed consent from patient using standardized text.** One hundred eighteen participants (N=59 TTG condition; N=59 VME condition) will be enrolled in this study.

13. **Optimize room conditions**
    - Room well lit (turn on overhead lights if necessary)
    - Patient preferably sitting up
    - Private setting (close door to patient room)
    - Interviewer should directly face patient
    - Patient should use reading glasses if they use one

14. **Assess patient's respiratory symptoms/morbidity (Forms 4-5-6), which includes the** Borg symptom score, Asthma Symptom Severity Index (ASSI), Chronic Bronchitis Symptom Questionnaire, COPD Severity Score (CSS), Airway Questionnaire (AQ-20), COPD Helplessness Index (CHI) and demographic and other clinical information.

15. **Assess patients' self-efficacy regarding respiratory inhaler use (Form 7)**

16. **Assess lung function. Perform spirometry pre and post-bronchodilator (after 2 puffs albuterol MDI via spacer) using portable KoKo spirometer. (Forms 8a and 8b).** These data will provide an objective measure of disease severity at the time of the hospitalization. We have previously demonstrated that this population has poor perception of airflow obstruction and spirometry is feasible during the inpatient setting.[16]

17. **Assess patient's inhaler technique using Inhaler checklists (Form 1;** for a MDI and, if applicable, Diskus®) by the trained assessor.

18. **Assess health literacy. Check vision (Form 9). Ask brief health literacy screen questions (Form 10a) for all participants and a**dminister STOHFLA questionnaire (Form 10b) if the patient's visual acuity is at least 20/50 in one eye (Check the patient's visual acuity in each eye using the Snellen Pocket Eye Chart (Form 11), otherwise, the patient is ineligible to complete the STOHFLA HL evaluation (will complete the BHLS questions).

    a) BHLS
    We want to find out what health care information is understood by patients and what is confusing to them, in order to help us understand what patients may find confusing.
    1) How often do you have problems learning about your medical condition because of difficulty understanding written information?
    2) How confident are you filling out medical forms by yourself?
    3) How often do you have someone help you read hospital material?

b) STOHFLA

Assess health literacy. Check vision (Form 9). Administer STOHFLA questionnaire (Form 10) if the patient's visual acuity is at least 20/50 in one eye (Check the patient's visual acuity in each eye using the Snellen Pocket Eye Chart (Form 11), otherwise, the patient is ineligible to complete the HL evaluation. Explain the purpose of this survey. Use the following text.

- We want to find out what health care information is understood by patients and what is confusing to them, in order to help us write things more clearly.
- Here are some medical instructions that you or anybody might see around the hospital. These instructions are in sentences that have some of the words missing.
- Where a word is missing, a blank line is drawn, and 4 possible words that could go in the blank appear just below it. I want you to figure out which of those 4 words should go in the blank, which word makes the sentence make sense.
- When you think you know which one it is, circle the letter in front of that word, and go on to the next one.
- When you finish the page, turn the page and keep going until you finish all the pages.
- Provide the patient a pen and a clipboard and ask them to complete the questionnaire (Form 10). If the patients ask for help with completing the questionnaire, instruct them to do the best they can and that you are not allowed to assist them. Only responses completed within 7 minutes are used in calculating a health literacy score. Record the start and stop time and any questions that were answered after 7 minutes had elapsed. Do not grade the STOFHLA until after the study visit is complete.

19. **Obtain randomization assignment from the study biostatistician.** Randomization will be block-stratified by level of health literacy to ensure an equal number of subjects within each stratum assigned to TTG and VME Patients will then receive either the TTG or VME intervention by the trained educator.

20. **Collect patient contact information and provide patient $25 in cash for participating in this study as well as parking pass/bus pass for their follow-up visit.** Record patient's social security number and obtain patients signature that they received the patient incentive.

    1. **Provide patients an appointment for an in-person interview 30 days after discharge (patients will be given a letter with their appointment time).** At the in-person interview, we will ask patients to complete a short interviewer-administered questionnaire about patient's respiratory symptoms/morbidity/self-reported ability to use MDI and Diskus® inhalers, quality of life (Forms 4 and 12). We will also assess lung function (spirometry) and use of inhalers (MDI and/or Diskus). Upon completing this portion the participant will receive $50 as well as parking pass/bus pass for their next follow-up visit.

OUTCOMES/MEASURES-AIM 2

The primary and secondary outcomes are listed below. Measures for each are described. Initial measurements (Vo; see Data Collection Schedule below) will be made prior to randomization; at V1, the trained educator will assess respiratory inhaler technique by asking the subject to demonstrate use of the MDI (and Diskus® if to be discharged home on fluticasone/salmeterol). Follow-up assessment, V2, will take place at 30 days (+/-7 days) post-hospital discharge.

Primary outcome

The primary outcome will be inhaler technique. We will define inhaler technique in two ways:

c) Correct Use (i.e., >75% of steps correct)
d) Mastery (i.e., perfect technique, 100% steps correct)

This outcome will be measured at V1 and V2. We will test our hypothesis that patients will have a reduced proportion of respiratory events when taught effective inhaler technique with TTG compared to BI education separately for the MDI and Diskus® devices after hospital discharge. We will employ the same checklists used in the E-TRaIN study (IRB Protocol #11-0248, high reliability, kappa >0.94). These checklists will be used to measure the primary outcome: ability to use inhalers (defined as (a) correct use of inhaler: >9/12 steps for MDI; >8/10 steps for Diskus®; and (b) mastery of inhaler technique, i.e., perfect technique: 12/12 steps for MDI; 10/10 steps for Diskus®).

The **primary outcome** is the proportion of participants with MDI misuse post VME vs. TTG education. This will provide data on the short-term effectiveness of the interventions. Longer-term retention (durability) will be examined 30 days post-hospital discharge using an intention-to-treat analysis. We have powered on this binary variable. [Table 1] However, because we recognize that there is more information about which steps are less critical and those that are more critical, I will also work with Robert Gibbons, a nationally recognized expert in biostatistics, to model a mixed effects ordinal regression model with random intercepts and time effects within correlation of each participant with terms for mode (VME and TTG), as well as interaction with time, to determine the predicted probability in each category for VME and TTG of how prevalence of misuse changes over time.[17] **Mediators include** health literacy[18] and vision level[19] as I have previously identified them as important mediators of baseline inhaler misuse and/or ability to learn correct technique.[8,10]

Secondary outcomes

Secondary outcomes will include:

d) Change in self-efficacy for respiratory inhalers (i.e., V0-V2). We will ask all patients to state how strongly they agree or disagree with the following statement "I am confident that I know how to use [insert inhaler name] correctly" for both MDI and Diskus®. The subject will be shown an example of each inhaler. Each item will be scored on a 5-point Likert scale (strongly disagree to strongly agree); the mean score will be used to measure self-efficacy. Cronbach's alpha will be calculated to evaluate internal consistency.
e) Symptom burden (i.e., BORG, ASSI, and CBSQ) at V1 and V2. Patient reported outcomes will be measured using a modified Borg dyspnea scale (Borg), Asthma Symptom Severity Index (ASSI), Chronic Bronchitis Symptom Questionnaire (CBSQ).
f) Acute care utilization for respiratory exacerbations at V2. Data will be collected using interviewer administrated surveys. *Respiratory events* are defined as: physician,

emergency room or hospital visit, or death. This composite endpoint is due to small sample size.

The comparative effectiveness of TTG versus VME with regards to these secondary outcomes will employ simple univariate and/or multivariate logistic regression, generalized estimating equations, or generalized non-linear mixed models, as appropriate. For each of these outcomes, we will evaluate whether health literacy level modifies the effectiveness of VME (Vs. TTG) by including an interaction term in the regression model. Model assumptions will be checked. Use of two inhalers will also provide the opportunity to evaluate if the comparative effectiveness of VME (vs. TTG) varies by device. A two-tailed p-value less than 0.05 will define statistical significance. The sample size calculations demonstrate that N=59 per group will provide 80% power for an intention-to-treat analysis to test for non-inferiority of VME vs. TTG.

Secondary Outcomes include quality of life (QOL),[20] patient reported outcomes,[20-21] lung function**,** utilization of health care services (outpatient visits, ED and hospital admissions, deaths), and self-efficacy of inhaler technique.[8,10] We will also evaluate the differential effects of VME vs. TTG by participant characteristics (e.g., age, health literacy). Finally we will evaluate the preliminary comparative durability of VME vs. TTG at 30 days post-hospital discharge. We will perform all tests separately for both MDI and Diskus devices.

Subjects in Aim 2 procedures will also be offered participation in the VME at Home sub-study. We will ask if subjects may be interested in participating in a secondary study to test the feasibility of using VME at home. The consent form will contain information with a separate line to initial if the subject would like to participate. If they initial this line, they will sign the separate VME at Home sub-study consent document. All subjects will be asked to use the VME on their own devices. They will be provided with an account to use the web-based VME, sent via email 3-5 days post-V2. Participants will be provided an appointment for an in-person interview 30 days after V2. A reminder phone call will be made once a week to ensure VME has been accessed. At 30-days post-V2, an in-person interview or phone interview will be completed to assess the following:

1. Assess patient's inhaler technique using Inhaler checklists (From 1; for a MDI and if applicable, Diskus®) by the trained assessor.

2. Acute care utilization for respiratory exacerbations. Data will be collected using interviewer administered surveys. *Respiratory events* are defined as: physician, emergency room or hospital visit, or death. This composite endpoint is due to small sample size.

3. VME at home Questionnaire

- Did you use the VME?
  - Yes
  - No
- How many times did you use the VME?
  - 0
  - 1-2
  - 3-5
  - Weekly
  - Daily
  - more than once per day

- If you did not use the VME, what were some reasons why?
- If you did use the VME:
  - Did the VME help you feel more confident about using your inhaler at home?
  - How long did it take you to use the VME at home?
  - Did you require help from anyone else in order to use the VME? Who helped you?
  - Is there any difference between the instructions in the video and those given by your doctor?
    - Yes
    - No
  - What device did you use to access the VME?
    - Computer
    - Tablet
    - Smartphone

DATA COLLECTION SCHEDULE-AIM 2

VISIT	Inpatient Visit<br>V0-V1		30 day FU<br>V2
Visit duration (minutes)	60 (total for V0 to V1)		60
Visit location	Inpatient		Outpatient
Visit type	In-person		In-person
Eligibility evaluation	■		
Consent	■		
Randomization	■		
Self Efficacy: Self-reported inhaler technique	■		■
Patient demonstration of inhaler technique	■	■	■
Vision Screen	■		
Health Literacy	■		
Spirometry before and after bronchodilator	■		■
Review of medications	■		■
Medication count/weigh			■
Questionnaire about symptom burden, respiratory exacerbations, acute care utilization	■		■
Discharge Questionnaire		■	

Vo: initial assessment V1: after instruction V2: 30 days post-discharge follow-up

This study will require 24 months:

5. Develop/pilot test interactive video (6 month)
6. Training of personnel (1 month)

7. Begin recruitment (1-3 participants/week, for 15 months)
8. Complete follow-up of last enrollee (1 month after last enrolled participant)
9. Data analyses and preparation of final report (2 months).

The research will take place on the inpatient wards of UCM.

ANALYSES-AIM 2

Analysis plan. We will use means, medians, proportions, scatterplots, and histograms to describe the data. We will test for group differences using the *t*-test, the Wilcoxon rank sum test, Pearson's chi-squared test, or Fisher's exact tests as appropriate. We will adhere to the intention-to-treat principle when testing for differences by intervention type. The **primary outcome** will be correct inhaler technique; chi-squared tests will be used to compare post VME vs. post TTG prevalence of inhaler misuse (<75% of steps correct). **Secondary outcomes** will include change in self-efficacy for respiratory inhalers, symptom burden (i.e., BORG and CSS), self-reported utilization of health care services for respiratory exacerbations, basic disease knowledge, and QOL. The comparative effectiveness of VME versus TTG with regards to these secondary outcomes will employ simple univariate and/or multivariate logistic regression, as appropriate. For each of these outcomes, we will evaluate whether patient characteristics (vision, age, and/or health literacy level) modifies the effectiveness of VME versus TTG by evaluating interactions in the regression model. A two-tailed p-value less than 0.05 will define statistical significance. The sample size calculations (see below) demonstrate that n=59 per group will provide 80% power for an intention-to-treat analysis.

Sample size. (Table 1) The main outcome is correct MDI use (>75% steps correct). In my preliminary studies above, the proportion of participants with correct MDI use after TTG was 88-100% (n=98, n=50, respectively).[8,10] These data (expected proportion of correct MDI use of 0.95), along with a noninferiority effect size of -0.10, were used to calculate the sample size.[22] We will enroll 142 participants over 15 months and will have sufficient power (0.8) to determine if VME is non-inferior to TTG, at a significance level of p<0.05. We have experience recruiting participants and have been able to recruit up to 12 participants per month; therefore, we do not foresee any problems with recruiting this number of participants in this timeline.

Table 1: Sample Size per intervention (VME/TTG)

	**Power**				
**EFFECT SIZE**	**0.60**	**0.70**	**0.80**	**0.90**	**0.95**
**-0.05**	137	179	236	326	412
**-0.10**	35	45	59	82	103
**-0.15**	16	20	27	37	46

<u>Interim analyses:</u> After 50% of the target sample size have completed the study (59 participants have completed the study) or after 50% of the study period (~7 months), whichever occurs first, results of an interim analysis will be provided to a Data Safety and Monitoring Board (Drs. Dana Edelson, MD; Steve White, MD; Neda Laiteerapong, MD; physicians who are not study investigators). Investigators and research assistants will remain masked to the results of the interim analyses until the end of the study. The DSMB will be asked to consider issues related to study performance (enrollment rates, study completion rates) and differences in outcomes between groups and provide recommendations to the PI and the IRB whether to continue the study or terminate enrollment.

<u>BENEFITS AND RISKS AIMS1 AND 2</u>

Benefits

We are not aware of any potential benefits to study subjects.

Risks

Potential risks include:

1. The subject may be uncomfortable answering some interview questions. They can refuse to answer any question.

2. The lung function test is safe and is commonly used to measure the severity of COPD but can cause some minor chest soreness or lightheadedness; study test will be done by a trained member of the research staff with the subject in a seated position. To further minimize the risk of adverse events with spirometry, we will not perform spirometry in subjects with SBP>180 or DBP>100 mm Hg.

3. Two puffs of albuterol (for post-bronchodilator spirometry) is generally well tolerated. We have not had adverse events with 2 puffs albuterol after enrolling >170 subjects in the SARI, COAT, and TURI studies (IRBs# 15729A, 14831A, 16900A). In some cases, however, 2 puffs of albuterol can cause jitteriness, anxiety, and tachyarrhythmias. To minimize this risk, we will withhold albuterol in subjects who are hospitalized with tachyarrhythmias or palpitations or report problems after using albuterol.

4. Long acting beta-2-agonists (salmeterol or formoterol). These medications are generally well-tolerated, but can have albuterol-like adverse effects; in addition, these agents have been associated with increase risk of asthma-related deaths.

5. Inhaled corticosteroids (fluticasone/budesonide). These medications are well tolerated, but can cause thrush, dysphonia, and other less common adverse effects.

6. Loss of confidentiality. To help ensure that patients' health information remains private, we will restrict access to data collected for our study to study personnel only (via use of password-protection and locked cabinets for study documents). Study ID numbers will be generated and will be used when discussing and/or reviewing data at study meetings. Also, study reports will report results in aggregate and not contain information that can be used to identify individual patients.

Data Safety Monitoring Plan-AIM 2

The PI will review monthly data reports reviewing recruitment, consent, intervention activities, adverse events, and follow-up. Although few, if any, intervention-related adverse events are anticipated in this education-related study, the study population is at risk for poor outcomes, including life-threatening exacerbations and death. The PI will comply with prompt reporting of any unanticipated events and the Data Safety and Monitoring Board (DSMB) (Dana Edelson, MD, Steve White, MD, Neda Laiteerapong, MD). The DSMB (see interim analysis above) will also review results of the interim and final analyses.

PATIENT INCENTIVES-AIM 1 AND 2

Participants will be provided $25 for completing the initial baseline interview (only interview for AIM 1, first interview for AIM 2) and $50 completing the post-hospital discharge in-person interview. Total possible monetary compensation is $25 for AIM 1 and $75 for AIM 2. Participants in the VME at home sub-study will be provided $50 for completing the 30-day in-person interview or $10 for completing the interview via phone. For AIM 2, participants will also be provided parking passes or bus passes for their follow-up visits as we recognize that this cost burden should not fall on the participant for participating in our study. Monetary compensation will be reduced, based on the extent to which the participante completes the study. For both AIMs all tests for this study will be done free of charge.

BIBLIOGRAPHY

1. Morbidity and Mortality 2007 Chart Book on Cardiovascular, Lung, and Blood diseases. Available at: http://www.nhlbi.nih.gov/resources/docs/07-chtbk.pdf. Accessed June 06, 2012.

2. Jencks SF, Williams MV, Coleman EA. Rehospitalizations among patients in the Medicare fee-for-service program. N Engl J Med 2009;360(14):1418-1428. PMID: 19339721.

3. Hanania NA, Darken P, Horstman D, Reisner C, Lee B, Davis S, Shah T. The efficacy and safety of Fluticasone Propionate (250ug) /Salmeterol (50ug) combined in the Diskus® Inhaler for the treatment of COPD. Chest 2003;124:834-843. PMID: 12970006.

4. Celli BR, Thomas NE, Anderson JA, Ferguson GT, Jenkins CR, Jones PW, Vestbo J, Knobil K, Yates JC, Calverley PMA. Effect of pharmacotherapy on rate of decline of lung function in chronic obstructive pulmonary disease. Results from the TORCH Study. Am J Resp Crit Care Med 2008;178:332-338. PMID: 18511702.

5. Melani, AS, Bonavia M, Cilenti V, Cinti C, Lodi M, Martucci P, Serra M, Scichilone N, Sestini P, Aliani M, Neri M, on behalf of the Gruppo Educazionale Associazione Italiana Penumologi Ospedalieri (AIPO). Inhaler mishandling remains common in real life and is associated with reduced disease control. Respir Med 2011;105:930-938. PMID: 21367593.

6. Dolovich MB, Ahrens RC, Hess DR, Anderson P, Dhand R, Rau JL, Smaldone GC, Guyatt G. Device selection and outcomes of aerosol therapy: Evidence-based guidelines: American College of Chest Physicians/American College of Asthma, Allergy, and Immunology Chest. 2005;127:335-71. PMID: 21367593.

7. The Global initiative for Chronic Obstructive Lung Disease (GOLD) Guidelines: Available at: http://www.goldcopd.com/. Accessed June 6, 2012.

7.5 Guidelines for the Diagnosis and Management of Asthma (EPR-3). Available at: https://www.nhlbi.nih.gov/guidelines/asthma/. Accessed June 6, 2012.

8. Press VG, Shah LM, Lewis S, Ivy K, Charbeneau J, Mazurek A, Badlani S, Naureckas E, Krishnan JA. Misuse of respiratory inhalers in hospitalized patients with asthma or COPD. J Gen Intern Med 2012;26(6), 635-642. PMID: 21249463 PMCID: PMC3101982.

9. Cohen DJ, Clark EC, Lawson PJ, Casucci BA, Flocke SA. Identifying teachable moments for health behavior counseling in primary care. Patient Educ Couns 2011;85:e8-e15. PMID: 21183305

10. Press VG, Arora VM, Lewis SL, Charbeneau J, Starkey J, Naureckas ET, Krishnan JA. Teaching the use of respiratory inhalers to hospitalized patients with asthma or COPD: A randomized trial (TURI). J Gen Intern Med May 2012. [Epub ahead of print.] PMID: 22592354.

11. Paasche-Orlow MK, Riekert KA, Bilderback A, Chanmugam A, Hill P, Rand CS. Tailored education may reduce health literacy disparities in asthma self-management. Am J Respir Crit Care Med 2005;172(8):980-986. PMID: 16081544 PMCID: PMC2718412.

12. Virchow JC, Crompton GK, Negro RD, Pedesen S, Magnan A, Seidenberg J, Barnes PJ. Importance of inhaler devices in the management of airway disease. Respir Med 2008;102:10-19. PMID: 17923402.

13. Jack BW, Chetty VK, Anthony D, Greenwalk JL, Sanchez GM, Johnson AE, Forsythe SR, O'Donnell JK, Paasche-Orlow MK, Manasseh C, Martin S, Culpepper L. A Reengineered Hospital Discharge Program to Decrease Rehospitalization. A Randomized Trial. Ann Intern Med 2009;150(3):178:187. PMID: 19189907. PMCID: PMC2738592.

14. Gerber BS, Brodsky IG, Lawless KA, Smolin LI, Arozullah AM, Smith EV, Berbaum ML, Heckerling PS, Eiser AR. Implementation and evaluation of a low-literacy diabetes education computer multimedia application. Diabetes Care 2005;28(7):1574-1580. PMID: 15983303.

15. Press VG, Shah LM, Lewis SL, Ivy K, Charbeneau JT, Mazurek A, Krishnan JA. Teach-to-goal intervention to teach respiratory inhaler technique in patients hospitalized for asthma or COPD. Abstract, accepted for poster presentation, Society of General Internal Medicine Annual Meeting May 2009.

16. Davis SQ, Permutt Z, Permutt S, Naureckas ET, Bilderback AL, Rand CS, Stein BD, Krishnan JA. Perception of airflow obstruction in patients hospitalized for acute asthma. *Annals of Asthma, Allergy, and Immunology.* 2009*;102(6)455-61.*

17. Hedeker D, Gibbons RD. Longitudinal Data Analysis. New Jersey: John Wiley & Sons; 2006.

18. Nurss JR, Parker RM, Williams MV, Baker, DW. Short Test of Functional Health Literacy in Adults (STOFHLA). Hartford: Peppercorn Books & Press; 2001.

19. Snellen Eye Chart: A Description and Explanation. Available at: http://precision-vision.com/index.cfm/feature/24_149/snellen-eye-chart---a-description-and-explanation.cfm. Accessed June 6, 2012.

20. Kendrick KR, Baxi SC, Smith RM. Usefulness of the modified 0-10 Borg scale in assessing the degree of dyspnea in patients with COPD and asthma. J Emerg Nurs 2002;26:216-222. PMID: 10839848.

21. Eisner MD, Trupin L, Katz PP, Yelin EH, Earnest G, Balmes J, Blanc PD. Development and validation of a survey-based COPD severity score. Chest 2005;127:1890-1897. PMID: 15947299.

22. Hung HMJ, Wang SJ, O'Neill R. A regulatory perspective on choice of margin and statistical inference issue in non-inferiority trials. Biom J 2005;47(1):28-36. PMID: 16395994.

The UNIVERSITY OF CHICAGO
The Division of the Biological Sciences • The University of Chicago Medical Center

CONSENT/AUTHORIZATION BY SUBJECT FOR PARTICIPATION IN A RESEARCH PROTOCOL

Protocol Number: IRB12-1844
Name of Subject: ________________________________
Medical History Number: __________________________

STUDY TITLE: Video vs. TTG Respiratory inhaler technique Assessment and InstructioN (V-TRaIN)

Doctor Directing Research: Dr. Valerie Press
Address: 5841 South Maryland Ave.
MC 5000
Chicago, IL 60637
Telephone Number: 773-702-5170

You are being asked to participate in a research study. A member of the research team will explain what is involved in this study and how it will affect you. This consent form describes the study procedures, the risks and benefits of participation, as well as how your confidentiality will be maintained. Please take your time to ask questions and feel comfortable making a decision whether to participate or not. This process is called informed consent. If you decide to participate in this study, you will be asked to sign this form.

WHY IS THIS STUDY BEING DONE?

Asthma and Chronic Obstructive Pulmonary Disease (COPD) are common lung conditions. Respiratory inhalers are commonly prescribed to patients with these conditions. Many patients have difficulty using inhalers.

You are being asked to join a research study because you have asthma or COPD, are hospitalized, and will be prescribed respiratory inhalers such as albuterol through a metered dose inhaler (MDI). There are no experimental drugs or experimental procedures being done during this study.

The purpose of this study is to evaluate the effectiveness of one or two different ways to teach subjects while hospitalized how to use respiratory inhalers and to follow-up after discharge home from the hospital to determine durability of the education.

HOW MANY PEOPLE WILL TAKE PART IN THE STUDY?

About 262 people will take part in this study at the University of Chicago.

WHAT IS INVOLVED IN THE STUDY?

There will be one visit in the hospital and one visit after you are discharged from the hospital. Each visit will last about 1 hour. The second visit will take place 30 days following discharge from your initial hospitalization.

During your initial hospital visit we will do the following:

- An interviewer will ask you a few questions about your health and complete a survey.
- You will have a spirometry. You will be asked to blow into a tube so that we can measure the severity of your lung disease.
- Your vision will be assessed using a standard assessment tool called a Snellen Card (eye chart).
- We will ask a few verbal questions and then use a brief reading test called a STOFHLA. This will help us get an understanding of how difficult it is for patients to read and understand medical information.
- You will be asked to take 2 puffs of albuterol, and then the spirometry will be repeated. The use of albuterol for the spirometry is part of the research study. It will help us measure the severity of your lung disease. Albuterol is used to prevent breathing difficulties by relaxing and opening air passages to the lungs to make breathing easier and spirometry is used to check lung function. Albuterol is approved by the Food and Drug Administration (FDA) for this purpose.

You will then receive one of two types of education about using respiratory inhalers. You will be randomized (like the flipping of a coin) to receive either a teach-to-goal educational intervention or the video module educational intervention. Both educational interventions will provide you information about how to use respiratory inhalers. An aerochamber ("spacer") will be provided for your use during the assessment of the inhaler (MDI) technique before and after education

<u>Video Module Education Group</u>

- If you are in this group, an educator will show you how to use the tablet to access the video education modules to complete the surveys; the video will show you how to use the albuterol inhaler and if applicable, the fluticasone/salmeterol (Advair Diskus®) inhaler (or the albuterol and budesonide/formoterol (Symbicort ®) inhaler) and will give you written instructions.
- You will receive teaching for the inhalers your doctors have prescribed through the video. Then there will be questions that you answer on the tablet to assess how well you understand how to use the inhaler(s).
- You will receive instructions by video one or multiple times, depending on how much you understand after getting one round of instructions.
- A second research assistant will ask you to show them how you use each respiratory inhaler after the first and other rounds of instructions if needed. This second research

assistant will notify your doctor in the hospital about how well you use your respiratory inhalers after these round(s) of teaching.

You will also receive a brief education about your asthma or COPD. This will be provided to you by the educator.

You will get standard of care for the medical treatment of asthma or COPD. This study will not change how your asthma or COPD is treated with medications.

Teach-to-Goal Group

- If you are in this group, an educator will show you how to use the albuterol inhaler and if applicable, the fluticasone/salmeterol (Advair Diskus®) inhaler (or the albuterol and budesonide/formoterol (Symbicort ®) inhaler) and will give you written instructions.
- You will receive teaching for the inhalers your doctors have prescribed. Then the educator will ask you to show them how you use each inhaler. The educator will correct any mistakes you make and ask you to show them again how to use each inhaler.
- You will receive instructions one or multiple times, depending on how much you understand after getting one round of instructions.
- A second research assistant will ask you to show them how you use each respiratory inhaler after the first and other rounds of instructions if needed. This second research assistant will notify your doctor in the hospital about how well you use your respiratory inhalers after these round(s) of teaching.

Follow-Up Visits

You will be asked to return to the hospital 30 days after your initial visit. The following will happen at these visits:

- An interviewer will ask you a few questions about your health and complete a survey.
- You will have spirometry. You will be asked to blow into a tube so that we can measure the severity of your lung disease.
- You will be asked to take 2 puffs of albuterol, then spirometry will be repeated. The use of albuterol for the spirometry is part of the research study. It will help us measure the severity of your lung disease.

We would like to contact you in the future and ask if you wanted to participate in other research studies. You will have the opportunity, at that time, to decide whether or not to participate in any study for which you may qualify. You will be asked to consider a separate informed consent document for the individual study you are being considered for at that time.

_______ (initial) I agree to be contacted in the future regarding participation in other research studies.
_______ (initial) I **do not** agree to be contacted in the future regarding participation in other research studies

We would like to ask some patients to try using the VME at home. You would be given an account to access a website from your own computer, tablet, or phone so that you could watch and use the VME at home. 5-10 people will be asked to try using VME at home. You do not have to participate at home if you do not want to, you can still participate in the hospital without using VME at home.

_______ (initial) I agree to be contacted about using VME at home. My email address to receive access to the VME is:
____@_____________________________

_______ (initial) I **do not** agree to be contacted about using VME at home.

INFORMATION TO BE COLLECTED

During this study, Dr. Press and her research team will collect information about you for the purposes of this research. This includes your name, relative's names and addresses, phone number, address, social security number, medical record number, initials, date of birth, height, weight, ethnicity, medical history, pharmacy and hospital records, the results of physical exams, questionnaires and date of study entry and dates of study procedures. We collect your relative's information to reach you. If you prefer, we do not need to obtain this from you.

HOW LONG WILL I BE IN THE STUDY?

You will be in this study for about 1 month.

Dr. Press may decide to take you off of the study without your consent if:

- You are unable to meet the requirements of the study;
- Your medical condition changes;
- New information becomes available that indicates that participation in this study is not in your best interest; or
- If the study is stopped.

WHAT ARE THE RISKS OF THE STUDY?

You may be uncomfortable answering some questions about your health.

Spirometry can cause some chest soreness or dizziness.

Side effects of Albuterol can include:

- Feeling nervous
- Shakiness

Side effects of Advair or Symbicort can include:

- Diarrhea
- Dizziness
- Headache
- Anxiety
- Fast heart beat
- High blood sugar
- High blood pressure
- Yeast infections
- Difficulty falling asleep
- Advair includes Salmeterol and Fluticasone. In rare cases, in patients with asthma, Salmeterol can cause **life-threatening** asthma attacks and **death**.
- Symbicort includes Budesonide and Formoterol. In rare cases, in patients with asthma, Formeterol can cause **life-threatening** asthma attacks and **death**.

ARE THERE ANY BENEFITS TO TAKING PART IN THE STUDY?

If you agree to take part in this study, there may or may not be direct medical benefit to you. We hope the information learned from this study will aid in our understanding of how best to train patients to use respiratory inhalers correctly.

WHAT OTHER OPTIONS ARE THERE?

You may choose not to participate. You do not need to join this study to receive medical care. You can speak to your health care provider about appropriate use of respiratory inhalers.

The decision whether or not you wish to participate in this study will <u>not</u> affect your care at the University of Chicago Medical Center.

WHAT ARE THE COSTS?

Clinical services provided during a clinical trial are either research-related or related to usual medical care. Research-related services are done to complete the research and the costs are not the responsibility of you or your insurance.

The costs that are considered research-related for this study include the following**:** Spirometry, the 2 puffs of albuterol before the second spirometry measurements, spacer for MDI, vision assessment (if required) and subject education provided by the research staff.

Usual medical care costs include any and all services that are considered medically necessary for your disease. These costs include hospital stay, prescribed respiratory inhaler (albuterol inhaler, Advair discus inhaler or symbicort inhaler) and any other medications you may be taking to control your asthma or COPD. The cost of this usual, ongoing medical care will be the responsibility of you or your insurance, and may include deductibles and co-payments. Similarly, this care will be subject to all the same requirements and restrictions of your insurance.

If you suffer an unanticipated injury as a direct result of this research and require emergency medical treatment, the University of Chicago Medical Center will provide such treatment at the University of Chicago Medical Center at no cost to you. You must notify Dr. Press as promptly as possible after your injury in order to receive this care. An injury is "unanticipated" if it is not one of the known effects of a study drug, medical device or procedure, and is not the result of your disease or condition. The costs of any non-emergency care for such an injury will be billed to you or your insurance company in the ordinary manner. If you think that you have suffered a research related injury, you must let Dr. Press know right away.

WILL I BE PAID FOR MY PARTICIPATION?

For participation in this study you will receive $25 for completing the interview in the hospital and another $50 for completing the in-person interview 1 month after hospital discharge (give or or take one week). You will be paid up to $75 total for completing this study. You will be paid in cash or gift card. You will also receive bus passes or parking passes for your follow-up visit.

WHAT ABOUT CONFIDENTIALITY?

Study records that identify you will be kept confidential. Data will be stored in a locked office, or in password protected computers. Only research staff involved in the study will have access to the data except as specified below. The data collected in this study will be used for the purpose described in this form. By signing this form, you are allowing the research team access to your medical records, which include Protected Health Information. Protected Health Information (PHI) consists of any health information that is collected about you, which could include your medical history and new information collected as a result of this study. The research team includes all of the individuals indicated on this consent form and other personnel involved in this study at the University of Chicago.

As part of the study, Dr. Press and her research team will report the results of your study-related procedures and tests explained above to the data safety monitoring board (a group of reviewers who monitor safety data during the course of the study) and the medical monitor (a physician who reviews subject safety during the course of the study). To protect your confidentiality, all names will be deleted from study records. You will be identified by a code and study number. The information shared could include date of birth, ethnicity, results of physical exams, height, weight, vital signs, blood tests, medical history, and the results of your questionnaires. This information is being looked at to review the results of the study, to monitor the safety of participants, to verify the accuracy of the data and compliance with the protocol.

Your records may be reviewed by federal agencies whose responsibility is to protect human subjects in research including the Food and Drug Administration (FDA) and Office of Human Research Protections (OHRP). In addition, representatives of the University of Chicago may also view the records of the research. These representatives could include quality assurance and billing personnel in addition to the Institutional Review Board, a committee that oversees the research at the University of Chicago. If your research record is reviewed by any of these

groups, they may also need to review your entire medical record. The results from tests and/or procedures performed as part of this study may become part of your medical record.

If health information is shared outside the University of Chicago, the same laws that the University of Chicago must obey may not protect your health information.

During your participation in this study, you will have access to your medical record. Dr. Press is not required to release to you information that is not part of your medical record.

The University of Chicago's Comptroller's office will have access to your name, address and social security number when processing your payment.

This consent form will be kept by the research team for at least 6 years. The study results will be kept in your research record and be used by the research team indefinitely. At the time of study completion, either the research information not already in your medical record will be destroyed or information identifying you will be removed from study results. Any research information in your medical record will be kept indefinitely.

Data from this study may be used in medical publications or presentations. Your name and other identifying information will be removed before this data is used. If we wish to use identifying information in publications, we will ask for your approval at that time.

A description of this clinical trial will be available on http://www.ClinicalTrials.gov, as required by U.S. Law. This Web site will not include information that can identify you. At most, the Web site will include a summary of the results. You can search this Web site at any time.

WHAT ARE MY RIGHTS AS A PARTICIPANT?

Taking part in this study is voluntary. If you choose not to participate in this study, your care at the University of Chicago/University of Chicago Medical Center will not be affected. You may choose not to participate at any time during the study. Leaving the study will not affect your care at the University of Chicago/University of Chicago Medical Center.

If you choose to no longer be in the study and you do not want any of your future health information to be used, you must inform Dr. Press in writing at the address on the first page. Dr. Press and the study sponsor may still use your information that was collected prior to your written notice.

You will be informed of any new significant information that may affect your willingness to continue participation.

You will be given a signed and dated copy of this document. This consent form document does not have an expiration date.

WHOM DO I CALL IF I HAVE QUESTIONS OR PROBLEMS?

You have talked with ________________________a member of the research staff at the University of Chicago about this study and you had the opportunity to ask questions concerning any and all aspects of the research. Dr. Press or her study staff will continue to be available to answer any questions you have about this research study or your participation in the study.

If you have further questions about the study, you may call Dr. Press at 773-702-5170.

If you have a research related injury, illness, or side effect you should immediately contact Dr. Press at 773-702-5170. In case of emergency call 773-702-6800 and ask for page # 2346.

If you have any questions concerning your rights as a research study participant, you may contact the Institutional Review Board (IRB), which is concerned with the protection of subjects in research projects. You can contact the IRB in writing or by phone.

Institutional Review Board (IRB)
University of Chicago
McGiffert Hall, 2nd floor
5751 S. Woodlawn Avenue
Chicago, Illinois 60637.
(773) 702-6505, office hours are 8:30 am - 5:00 pm, Monday through Friday.

CONSENT

1. SUBJECT

The research project and the procedures associated with it have been explained to me. The experimental procedures have been identified and no guarantee has been given about the possible results. I will receive a signed copy of this consent form for my records.

I agree to participate in this study. My participation is voluntary and I do not have to sign this form if I do not want to be part of this research study.

____________________________________ Date: _____________

Signature of Subject Time: ________ AM/PM (*circle*)

2. PERSON OBTAINING CONSENT

I have explained to _________________________________ the nature and purpose of the study and the risks involved. I have answered and will answer all questions to the best of my ability. I will give a signed copy of the consent form to the subject.

____________________________________ Date: _____________

Signature of Person Obtaining Consent Time: ________ AM/PM (*circle*)

3. INVESTIGATOR/PHYSICIAN

___________________________________ Date: _____________

Signature of Investigator/Physician Time: ________ AM/PM (*circle*)
