# Supplementary material for: Effectiveness of Virtual vs In-Person Inhaler Education for Hospitalized Patients With Obstructive Lung Disease: A Randomized Clinical Trial
Source: JAMA Netw Open. 2020 Jan 3;3(1):e1918205. doi: 10.1001/jamanetworkopen.2019.18205 (PMC6991242; doi:10.1001/jamanetworkopen.2019.18205)
Supplement: Supplement 2. — eFigure 1. A Conceptual Model of In-person TTG and V-TTG eFigure 2. Virtual Teach-to-Goal Screenshots eFigure 3. Metered Dose Inhaler Checklist eTable. V-TTG versus TTG Results [file jamanetwopen-3-e1918205-s002.pdf]

Supplementary Online Content

Press VG, Arora VM, Kelly CA, Carey KA, White SR, Wan W. Effectiveness of virtual vs in-person inhaler education for hospitalized patients with obstructive lung disease: a randomized clinical trial. *JAMA Netw Open*. 2020;3(1):e1918205. doi:10.1001/jamanetworkopen.2019.18205

eFigure 1. A Conceptual Model of In-person TTG and V-TTG

eFigure 2. Virtual Teach-to-Goal Screenshots

eFigure 3. Metered Dose Inhaler Checklist

eTable. V-TTG versus TTG Results

This supplementary material has been provided by the authors to give readers additional information about their work.

© 2020 Press VG et al. *JAMA Network Open.*

eFigure 1. A Conceptual Model of In-person TTG and V-TTG

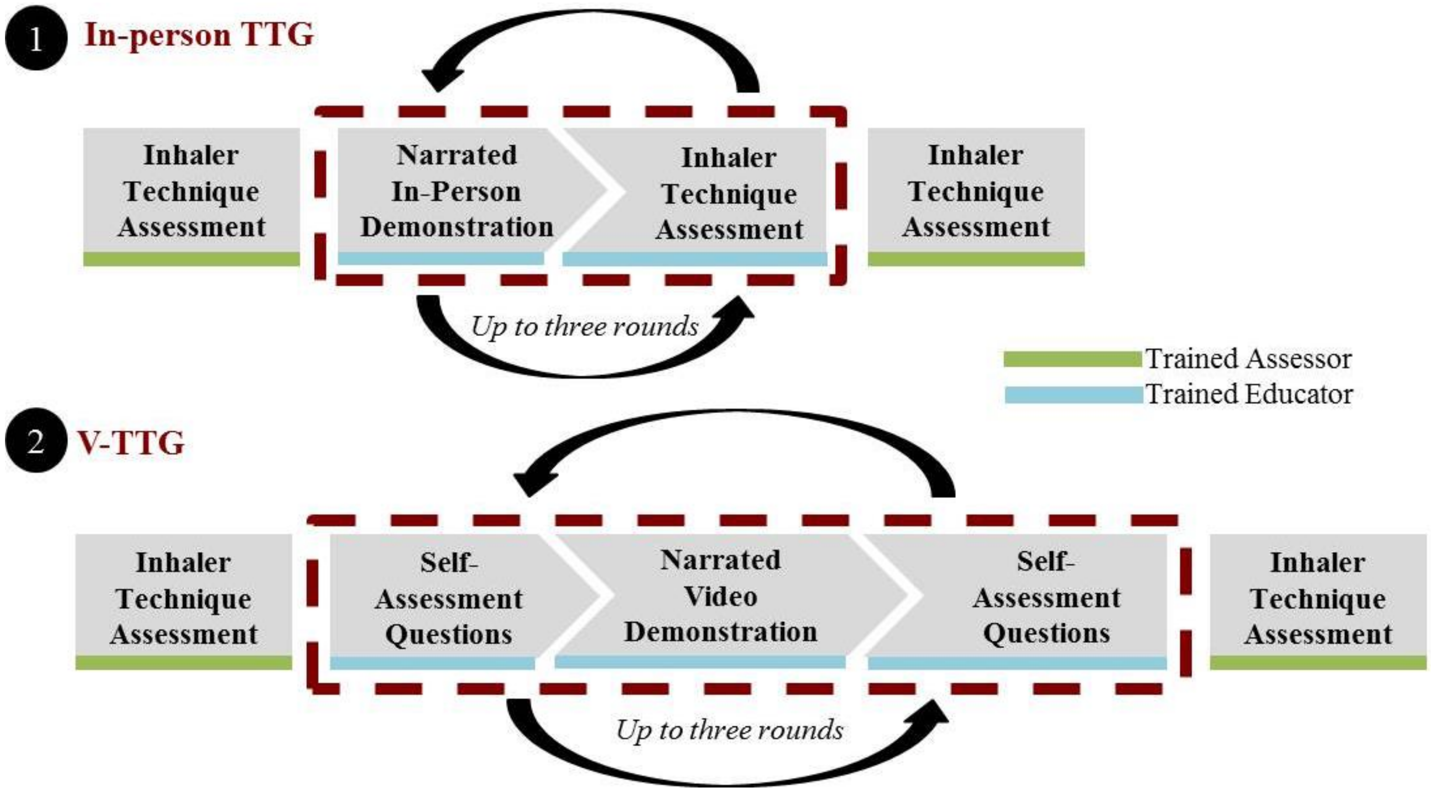

Both models include rounds of assessment and education. The in-person teach-go-goal (TTG: "1") assessments are conducted with a trained assessor (blue); the virtual TTG (V-TTG: "2") assessments are self-assessment questions embedded within the learning module. The in-person TTG education uses live demonstration and verbal instruction provided by the trained educator; the V-TTG education uses narrated demonstration with the handheld table provided to the participant by the trained educator. For the purpose of the study, trained assessors (research assistants; green) who were blinded to the assigned intervention observed all participants, including those in the in-person TTG and V-TTG cohorts before and after the educational session to collect data on inhaler technique performance.

© 2020 Press VG et al. *JAMA Network Open.*

eFigure 2. Virtual Teach-to-Goal Screenshots

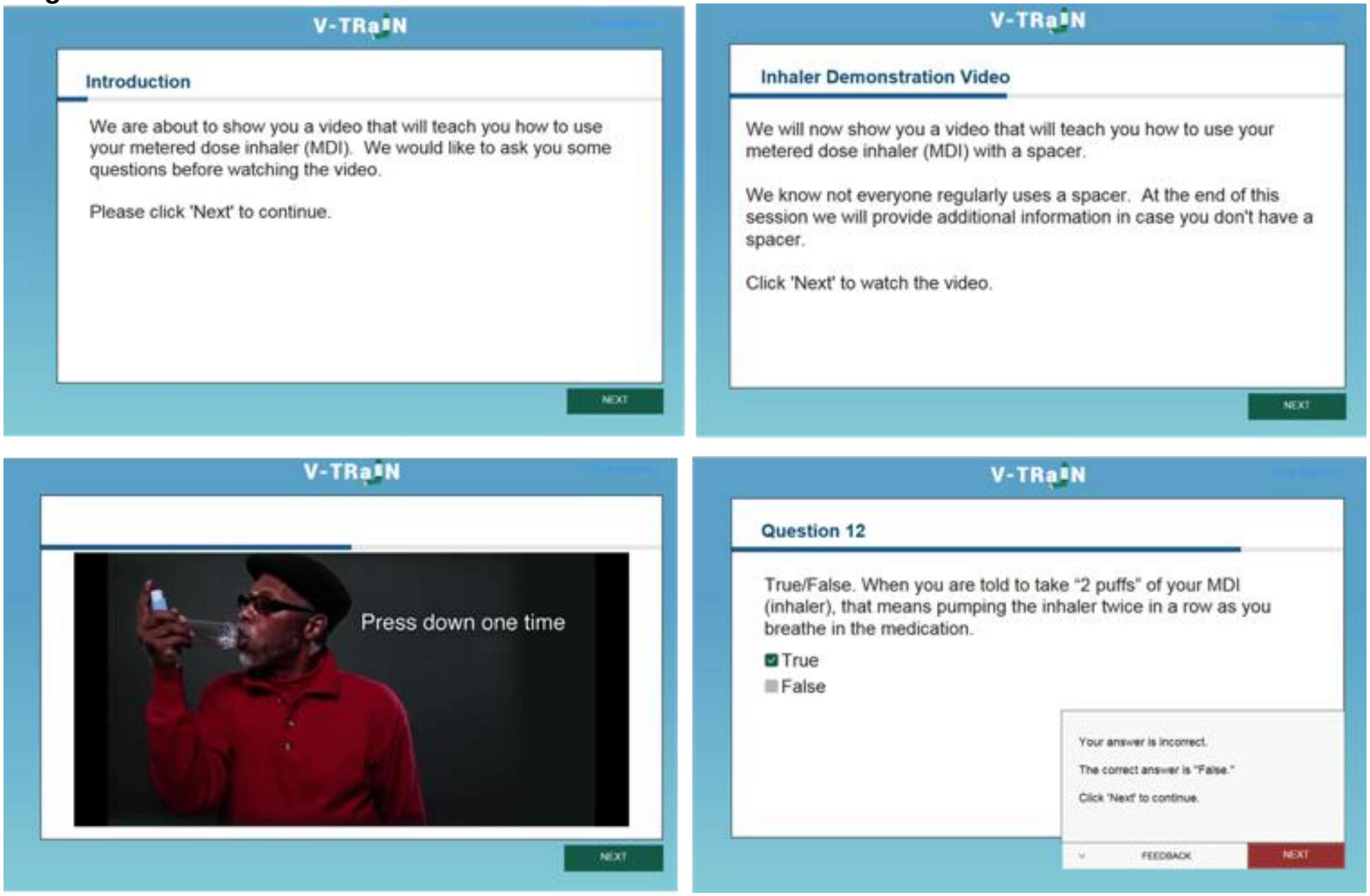

A series of screenshots to demonstrate the Virtual Teach-To-Goal "VTRAIN" intervention. A: The introductory slide presented after a brief narrated introduction; B: An example of a pre-video demonstration assessment item (one of 6 pre-questions); C: An example of the demonstration with text overlay (there is also narration); and D: An example of a post-video demonstration assessment item (one of 7 post-questions); of note, this example shows how upon getting a question incorrect on the third (final) round, the participant is provided with the correct answer.

eFigure 3. Metered Dose Inhaler Checklist

Step #	Step Description	Pre-education assessment		Post round 1		Post round 2		Post round 3	
		0=wrong	*1=correct*	*0=wrong*	*1=correct*	*0=wrong*	*1=correct*	*0=wrong*	*1=correct*
1	Removes caps from the inhaler and spacer	☐0	☐1	☐0	☐1	☐0	☐1	☐0	☐1
2	Shakes the inhaler up and down	☐0	☐1	☐0	☐1	☐0	☐1	☐0	☐1
3	Attaches inhaler to the spacer	☐0	☐1	☐0	☐1	☐0	☐1	☐0	☐1
4	Breathes out (empties lungs)	☐0	☐1	☐0	☐1	☐0	☐1	☐0	☐1
5	When breathing out, does so away from the inhaler/spacer	☐0	☐1	☐0	☐1	☐0	☐1	☐0	☐1
6	Puts mouthpiece of spacer (attached to inhaler) into mouth with lips closed around mouthpiece	☐0	☐1	☐0	☐1	☐0	☐1	☐0	☐1
7	Activates the inhaler one time by pressing down on canister	☐0	☐1	☐0	☐1	☐0	☐1	☐0	☐1
8	Breathes in SLOWLY	☐0	☐1	☐0	☐1	☐0	☐1	☐0	☐1
9	Holds breath for at least 5 seconds	☐0	☐1	☐0	☐1	☐0	☐1	☐0	☐1
10	Removes inhaler/spacer from mouth	☐0	☐1	☐0	☐1	☐0	☐1	☐0	☐1
11	Breathes normally for 30-60 seconds	☐0	☐1	☐0	☐1	☐0	☐1	☐0	☐1
12	Repeats for second puff if directed to do so	☐0	☐1	☐0	☐1	☐0	☐1	☐0	☐1
			Score ___/12		*Score ___/12*		*Score ___/12*		*Score ___/12*

The 12 step checklist to evaluate pre-education and post education (rounds 1 through 3) metered dose inhaler technique. Each correct step receives one-point, each incorrect step receives zero points. The total score is assessed for each assessment round (pre and post [round 1, round 2, round 3]) out of 12 maximum points. This checklist was previously validated and published with strong internal validity (kappa = 0.94).[11]

© 2020 Press VG et al. *JAMA Network Open.*

eTable 2. V-TTG versus TTG Results

Inhaler technique, n(%)	TTG (n=59)	V-TTG (n=59)
Post correct	49 (83)	41 (70)
30-day correct	32 (63)	26 (53)
Post mastery	28 (48)	17 (29)
30-day mastery	8 (14)	3 (5)
**Subgroup analyses**		
Post correct: adequate HL	39 (89)	35 (81)
30-day correct: adequate HL	28 (72)	22 (63)
Post correct: inadequate HL	10 (67)	6 (36)
30-day correct: inadequate HL	4 (33)	4 (29)
Post correct: COPD[a]	28 (82)	23 (66)
30-day correct: COPD[a]	16 (55)	14 (48)
Post correct: Asthma[a]	21 (84)	18 (75)
30-day correct: Asthma[a]	16 (73)	12 (60)

Abbreviations: HL = health literacy; COPD = chronic obstructive pulmonary disease; TTG = teach-to-goal; V-TTG = virtual teach-to-goal; n/a= not applicable.
[a] Denominator for COPD patients is 69 and for asthma patients is 49 at baseline and post-education.

© 2020 Press VG et al. *JAMA Network Open.*
