# Supplementary material for: Effectiveness of Virtual vs In-Person Inhaler Education for Hospitalized Patients With Obstructive Lung Disease: A Randomized Clinical Trial
Source: JAMA Netw Open. 2020 Jan 3;3(1):e1918205. doi: 10.1001/jamanetworkopen.2019.18205 (PMC6991242; doi:10.1001/jamanetworkopen.2019.18205)
Supplement: Supplement 3. — Data Sharing Statement [file jamanetwopen-3-e1918205-s003.pdf]

Data Sharing Statement

Press. Effectiveness of Virtual vs In-person Inhaler Education for Hospitalized Patients With Obstructive Lung Disease. *JAMA Netw Open*. Published January 03, 2020. 10.1001/jamanetworkopen.2019.18205

Data
Data available: Yes
Data types: Deidentified participant data, Data dictionary
How to access data: Those interested in this information should contact the corresponding author, Valerie Press (vpress@bsd.uchicago.edu).
When available: With publication

Supporting Documents
Document types: Statistical/analytic code, Informed consent form
How to access documents: Those interested in this information should contact the corresponding author, Valerie Press (vpress@bsd.uchicago.edu).
When available: With publication

Additional Information
Who can access the data: This data will be made available to anyone requesting this information for specific purpose(s) and analyses after a signed data access agreement is completed between the two parties and the IRB has provided approval.
Types of analyses: This data will be made available to anyone requesting this information for specific purpose(s) and analyses.
Mechanisms of data availability: This data will be made available after contact with the corresponding author has been made, a signed data access agreement is completed between the two parties, and the IRB has provided approval.
Any additional restrictions: N/A
